# The multi-peak adaptive landscape of crocodylomorph body size evolution

**DOI:** 10.1186/s12862-019-1466-4

**Published:** 2019-08-07

**Authors:** Pedro L. Godoy, Roger B. J. Benson, Mario Bronzati, Richard J. Butler

**Affiliations:** 1 School of Geography, Earth and Environmental Sciences, University of Birmingham, Birmingham, UK; 2 Department of Earth Sciences, University of Oxford, Oxford, UK; 3 Laboratório de Paleontologia de Ribeirão Preto, FFCLRP, Universidade de São Paulo, Ribeirão Preto, Brazil; 4 Present Address: Department of Anatomical Sciences, Stony Brook University, Stony Brook, NY 11794 USA

**Keywords:** Crocodylomorpha, Crocodyliformes, Body size evolution, Adaptive landscape, Phylogenetic comparative methods, Ornstein–Uhlenbeck models

## Abstract

**Background:**

Little is known about the long-term patterns of body size evolution in Crocodylomorpha, the > 200-million-year-old group that includes living crocodylians and their extinct relatives. Extant crocodylians are mostly large-bodied (3–7 m) predators. However, extinct crocodylomorphs exhibit a wider range of phenotypes, and many of the earliest taxa were much smaller (< 1.2 m). This suggests a pattern of size increase through time that could be caused by multi-lineage evolutionary trends of size increase or by selective extinction of small-bodied species. Here, we characterise patterns of crocodylomorph body size evolution using a model fitting-approach (with cranial measurements serving as proxies). We also estimate body size disparity through time and quantitatively test hypotheses of biotic and abiotic factors as potential drivers of crocodylomorph body size evolution.

**Results:**

Crocodylomorphs reached an early peak in body size disparity during the Late Jurassic, and underwent an essentially continual decline since then. A multi-peak Ornstein-Uhlenbeck model outperforms all other evolutionary models fitted to our data (including both uniform and non-uniform), indicating that the macroevolutionary dynamics of crocodylomorph body size are better described within the concept of an adaptive landscape, with most body size variation emerging after shifts to new macroevolutionary regimes (analogous to adaptive zones). We did not find support for a consistent evolutionary trend towards larger sizes among lineages (i.e., Cope’s rule), or strong correlations of body size with climate. Instead, the intermediate to large body sizes of some crocodylomorphs are better explained by group-specific adaptations. In particular, the evolution of a more aquatic lifestyle (especially marine) correlates with increases in average body size, though not without exceptions.

**Conclusions:**

Shifts between macroevolutionary regimes provide a better explanation of crocodylomorph body size evolution on large phylogenetic and temporal scales, suggesting a central role for lineage-specific adaptations rather than climatic forcing. Shifts leading to larger body sizes occurred in most aquatic and semi-aquatic groups. This, combined with extinctions of groups occupying smaller body size regimes (particularly during the Late Cretaceous and Cenozoic), gave rise to the upward-shifted body size distribution of extant crocodylomorphs compared to their smaller-bodied terrestrial ancestors.

**Electronic supplementary material:**

The online version of this article (10.1186/s12862-019-1466-4) contains supplementary material, which is available to authorized users.

## Background

Body size is related to many aspects of ecology, physiology and evolutionary history [1–6], and patterns of animal body size evolution are a long-standing subject of macroevolutionary investigation (e.g., [7–11]). As a major focus of natural selection, it is expected that significant variation should occur in the body size of animals, although confined within biological constraints, such as skeletal structure, thermoregulation and resource availability [4, 5, 12]. Furthermore, body size can often be easily measured or estimated from both fossil and modern specimens, and has therefore been widely used in phenotypic macroevolutionary studies [5, 7–9, 11, 13–17].

With few exceptions (e.g., [18, 19]), previous studies of tetrapod body size evolution have focused on mammals (e.g., [14–16, 20–24]) and dinosaurs or birds (e.g., [25–33]). Little is known, however, about other diverse and morphologically disparate clades. Among those, Crocodylomorpha represents an excellent group for studying large-scale evolutionary patterns, with a rich and well-studied fossil record covering more than 200 million years (i.e., oldest fossils from the Carnian, Late Triassic [34, 35]), as well as living representatives [36–38]. Previous works have investigated multiple aspects of crocodylomorph macroevolution, including spatial and temporal patterns of diversity [37–40], as well as morphological variation, disparity, and evolution, with a particular focus on the skull [41–48].

Nevertheless, studies quantitatively investigating macroevolutionary patterns of body size in crocodylomorphs have been restricted to particular time periods (e.g., Triassic-Jurassic body size disparity [49, 50]) or clades (e.g., metriorhynchids [51]), limiting broader interpretations. For instance, the impact of environmental temperature on the growth and adult body size of animals has long been acknowledged as an important phenomenon [4] and has been considered to have a significant influence on the physiology and distribution of extant crocodylians [52, 53]. There is also strong evidence for climate-driven biodiversity patterns in the group (e.g., [38, 39]). Nevertheless, it remains unclear whether extrinsic factors, such as temperature and geographic distribution, have impacted long-term patterns of crocodylomorph body size evolution [54].

Most of the earliest crocodylomorphs, such as *Litargosuchus* (Early Jurassic) and *Hesperosuchus* (Late Triassic), were small-bodied animals (with estimated total lengths of less than 1 m [55, 56]), contrasting with some giant forms that appeared later, such as the Late Cretaceous forms *Sarcosuchus* and *Deinosuchus* (possibly more than 10 m long [57, 58]), as well as with the intermediate to large sizes of extant crocodylians (1.5–7 m [59, 60]). The body size of extant species raises questions about what long-term macroevolutionary process (or processes) gave rise to the prevalence of larger body sizes observed in the present. This could be explained by directional trends of increasing body size through time (see [61]), differential extinction of small bodied taxa, or other factors, such as climate- or environment-driven evolutionary change (such as those related to ecological transitions between terrestrial and aquatic lifestyles). However, because patterns of body size evolution along phylogenetic lineages of crocodylomorphs have not been characterised, its causes are unaddressed.

### Model-fitting approach

Since the end of the last century, palaeontologists have more frequently used quantitative comparative methods to investigate the tempo and mode of evolution along phylogenetic lineages [62–64], including studies of body size evolution [5, 14, 15, 27, 29, 65]. More recently, numerous studies have employed a phylogeny-based model-fitting approach, using a maximum-likelihood or Bayesian framework to identify the best-fitting statistical macroevolutionary model for a given phylogenetic comparative dataset [31, 33, 66–70]. Many of those works have tested the fit of a uniform macroevolutionary model, with a single set of parameters applied across all branches of a phylogeny (e.g., [51, 69, 71, 72]). Uniform models are important for describing many aspects of phenotypic evolution and are often the null hypothesis in such studies. However, if the dynamics of evolutionary changes vary in more complex ways through time and space and among clades and environments (e.g., [73–77]) then uniform models might not be adequate to characterise this variation. For example, non-uniform models might be best supported when more restricted temporal and/or taxonomical scenarios are analysed, providing evidence of short-lived trends, adaptive peaks, and early bursts, However, this local scale variation in evolutionary dynamics are often “averaged” to more straightforward uniform models on large scales [75]. We sought to test this hypothesis with our analyses.

Incorporating biological realism into statistical models of evolution is challenging [78]. Many existing models are based on a Brownian motion (BM) process resulting from random walks of trait values along independent phylogenetic lineages [62, 79, 80]. Uniform Brownian motion has many interpretations. For example, it can be used as a model of drift, or of adaptive evolution towards lineage-specific selective optima that undergo random walks through time, and seems reasonable for describing undirected and unconstrained stochastic change [62]. Elaborations of BM models include the “trend” model, which incorporates a tendency for directional evolution by adding a parameter μ [81]. Furthermore, multi-regime “trend-like” models have also been proposed, in which the trend parameter (μ) undergoes clade-specific or time-specific shifts (G. Hunt in [33]).

The Ornstein–Uhlenbeck (OU) process [63, 66, 69, 82, 83] is a modification of Brownian motion that incorporates attraction (α) to a trait ‘optimum’ (θ). OU models describe the evolution of a trait towards or around a stationary peak or optimum value, at a given evolutionary rate. Thus, multi-regime OU models can account for the existence of multiple macroevolutionary regimes, which is consistent with the concept of a Simpsonian Adaptive Landscape [84, 85]. This conceptual framework has proved to be fruitful for characterizing macroevolutionary changes, encompassing ideas such as adaptive zone invasion (which are similar to the multiple macroevolutionary regimes of non-uniform OU models) and quantum evolution [76, 80, 86]. Macroevolutionary landscapes provide a conceptual bridge for dialogues between studies of micro- and macroevolution, and have benefitted from the subsequent advancements of molecular biology and genetics [87]. Within this paradigm, uniform models would primarily represent static macroevolutionary landscapes, with unchanged peaks (or maximum adaptive zones [11]) persisting through time and across the phylogeny [76, 80, 85], although still able to provide suitable explanations for the observed evolutionary patterns [75].

Many OU-based models typically require a priori adaptive hypotheses for inferring the trait optima of regimes [66, 83]. However, more recent methods attempt to solve this problem by estimating location, values and magnitudes of regime shifts without a priori designation of selective regimes [78, 88]. In particular, the SURFACE method [88] aims to identify shifts in macroevolutionary regimes, identified using AICc (Akaike’s information criterion for finite sample sizes [89]). Originally designated to identify “convergent” trait evolution across phylogenetic lineages, the SURFACE algorithm makes use of a multi-peak OU-model and can be a tool to determine heterogeneity of macroevolutionary landscapes [33, 90, 91].

In this work, we approach the study of crocodylomorph body size evolution by fitting a set of different uniform and non-uniform evolutionary models, aiming to characterise the changes in body size among many subgroups inhabiting different environments and encompassing substantial variation in morphology. This represents the first comprehensive investigation of large-scale patterns of body size evolution across the entire evolutionary history of crocodylomorphs.

## Methods

### Proxy for body size

Extinct Crocodylomorpha are morphologically diverse and frequently known from incomplete remains. Therefore, precise estimation of their body sizes, and those of comparable fossil groups, can be challenging (see [92, 93] for related considerations). There are many methods and equations for estimating crocodylomorph body size (either body mass or length) available in the literature. The most frequently used equations are derived from linear regressions based on specimens of modern species, using both cranial [57, 94–98] and postcranial [99, 100] measurements as proxies, even though some inaccuracy is expected (see Additional file 1 for further discussion).

We sought an appropriate proxy for studying body size across all crocodylomorph evolutionary history that also maximised available sample size, to allow as comprehensive a study of evolutionary history as possible. Thus, we decided to use two cranial measurements as proxies for total body length: total dorsal cranial length (DCL) and dorsal orbito-cranial length (ODCL), which is measured from the anterior margin of the orbit to the posterior margin of the skull (measurements were taken following [96]). By using cranial measurements instead of estimated total body length, we are ultimately analysing patterns of cranial size evolution in crocodylomorphs. Nevertheless, by doing this we also avoid the addition of errors to our model-fitting analyses, since previous works have reported problems when estimating total body length from cranial measurements, particularly skull length (e.g., [51, 93, 101, 102]), as the equations were formulated using modern species and different crocodylomorph clades are likely to have body proportions distinct from those of living taxa (see Additional file 1 for further discussion). Furthermore, the ranges of body sizes among living and extinct crocodylomorphs is considerably greater than the variation (i.e. error) among size estimates for a single species. Therefore, we expect to recover the most important macroevolutionary body size changes in our analyses even when using only cranial measurements. The use of ODCL, in addition to DCL, is justified as it allows us to examine the sensitivity of our results to changes in proportional snout length, as a major aspect of length change in crocodylomorph skulls results from proportional elongation or shortening of the snout [103–105]. Also, more taxa could be included in our analyses when doing so, because ODCL can be measured from some incomplete skulls.

The DCL dataset includes 219 specimens (representing 178 taxa), whereas the ODCL dataset includes 240 specimens (195 taxa). In total, measurements from 118 specimens (83 taxa) were collected via first-hand examination from specimens, using callipers and measuring tape. The remaining information was collected from the literature (98 specimens) or photographs (21 specimens) supplied by other researchers, and measurements were estimated using the software ImageJ (see Additional file 2 for the complete list of sampled specimens). We used mean values in those cases where we had cranial measurements for multiple specimens of the same taxon. For both the model-fitting and correlation analyses, we used log-transformed skull measurements in millimetres. However, to help us further interpret and discuss our results, total body length was subsequently estimated using the equations presented by [96].

### Phylogenetic framework

For the phylogenetic framework of Crocodylomorpha, our aim was to maximise taxon inclusion and to use a phylogenetic hypothesis that best represents the current consensus. We primarily used an informally modified version of the supertree presented by Bronzati et al. [37], which originally contained 245 taxa. We added recently published species, and removed taxa that have not yet received a formal description and designation. Also, species not previously included in phylogenetic studies but for which we had body size data were included based on the phylogenetic positions of closely related taxa (see Additional file 1 for more information on the construction of the informal supertree). Thus, our updated version of the supertree contains 296 crocodylomorph species, as well as nine closely related taxa used as outgroups for time-scaling the trees (see below).

**Fig. 1:**
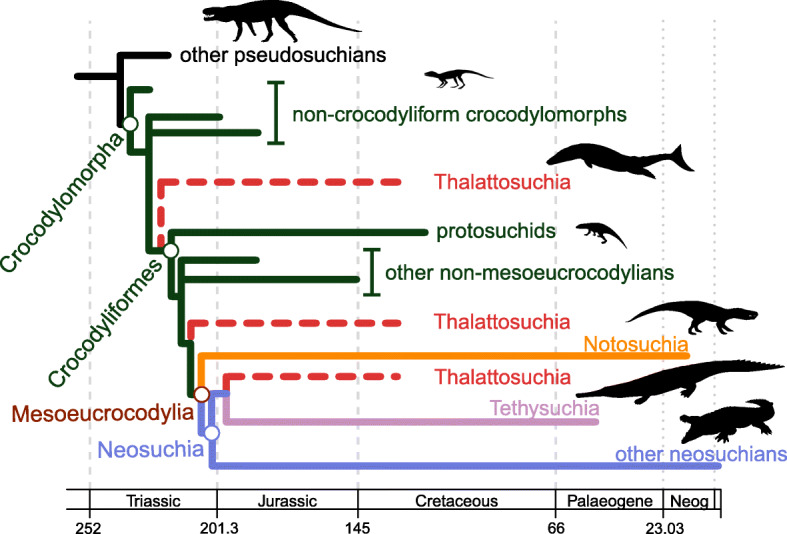
Simplified cladogram showing the phylogenetic relationships among crocodylomorphs and the alternative positions of Thalattosuchia (dashed red lines), following hypotheses proposed by [36, 37, 105, 109, 111]. Silhouettes are from phylopic.org

To accommodate major uncertainties in crocodylomorph phylogeny, we also conducted analyses using two alternative topologies, varying the position of Thalattosuchia. Thalattosuchians are Jurassic–Early Cretaceous aquatic crocodylomorphs, some of which were probably fully marine [106]. They have classically been placed within Neosuchia, as the sister taxon of Tethysuchia [103, 104]. Nevertheless, some authors have argued that this close relationship may result from the convergent acquisition of longirostrine snouts in both groups [103, 107], and some recent works have suggested multiple alternative positions for Thalattosuchia, within or as the sister group of Crocodyliformes (i.e., only distantly related to Neosuchia [105, 108–110]). Accordingly, to test the influence of uncertainty over the phylogenetic position of Thalattosuchia, we performed our macroevolutionary analyses using three distinct phylogenetic scenarios of Crocodylomorpha (Fig. 1). In the first, the more classic position of Thalattosuchia was maintained (Thalattosuchia as the sister taxon of Tethysuchia and within Neosuchia; as in the original supertrees of Bronzati et al. [36, 37]). In the two alternative phylogenetic scenarios, Thalattosuchia was placed as the sister group of either Crocodyliformes (as non-crocodyliform crocodylomorphs, following the position proposed by Wilberg [105]) or Mesoeucrocodylia (as the sister group of the clade formed by Neosuchia + Notosuchia in our topologies, following Larsson & Sues [111] and Montefeltro et al. [109]). Discrepancies among competing phylogenetic hypotheses do not concern only the “thalattosuchian problem” mentioned here. However, our decision to further investigate only the impact of the different positions of Thalattosuchia is based on its high taxic diversity and the impact that its phylogenetic position has on branch lengths across multiple parts of the tree, factors that can substantially alter macroevolutionary patterns detected by our analyses.

### Time-scaling method

Calibration of the phylogeny to time is a crucial step in comparative analyses of trait evolution [112], and the use of different methods may impact upon the inference of evolutionary models and the interpretation of results [113, 114]. As such, we decided to use a tip-dating approach using the fossilised birth-death (FBD) model [115]. The FBD method is a Bayesian total-evidence dating approach which uses a birth-death process that includes the probability of fossilization and sampling to model the occurrence of fossil species in the phylogeny and estimate divergence times (=node ages) [116–119]. Information on occurrence times of all species in the supertree (=tip ages) were initially obtained from the Paleobiology Database (PBDB) but were then checked using primary sources in the literature. Fossil ages were represented by uncertainty bounds of their occurrences. We then generated an “empty” morphological matrix for performing Bayesian Markov chain Monte Carlo (MCMC) analyses in MrBayes version 3.2.6 [120], following the protocol within the R package *paleotree* version 3.1.3 [121]. We used our supertree topologies (with alternative positions of Thalattosuchia) as topological constraints and set uniform priors on the age of tips based on the occurrence dates information. We used a uniform prior for the root of the tree (for all three topologies/phylogenetic scenarios), constrained between 245 and 260 Myr ago. This constraint was used because the fossil record indicates that a crocodylomorph origin older than the Early Triassic is unlikely [122–124]. For each topology, 10,000,000 generations were used, after which the parameters indicated that both MCMC runs seemed to converge (i.e., the Potential Scale Reduction Factor approached 1.0 and average standard deviation of split frequencies was below 0.01).

For each topology, we randomly sampled 20 trees (henceforth: FBD trees) from the posterior distribution after a burn-in of 25%. This resulted in 60 time-scaled, completely resolved crocodylomorph trees that were used in our macroevolutionary model comparisons. Similar numbers of trees were used in previous work on dinosaurs [33], mammals [24] and early sauropsids [92]. Analyses across these 60 trees allowed us to characterise the influence of topological and time-scale uncertainties on our results.

Previous studies have demonstrated that time-calibration approaches can impact phylogenetic comparative methods (e.g., [125]). Therefore, we also used other three time-scaling methods (*minimum branch length*, *cal3* and *Hedman* methods [18, 113, 126]). Differently from the FBD tip-dating method, these three methods belong to the category of a posteriori time-scaling (APT) approaches (sensu Lloyd et al. [126]), and were used as a sensitivity analysis (see Additional file 1 for further details on the employment of these methods). These additional time-scaling approaches were used only for our initial model comparisons (see below). APT methods were performed in R version 3.5.1 [127], using package *paleotree* [121] (*mbl* and *cal3* methods) and the protocol published by Lloyd et al. [126] (*Hedman* method). Results from macroevolutionary analyses using these APT methods were similar to those using the FBD trees (see the “Results” section) and are therefore not discussed further in the main text (but are included in Additional file 1).

### Macroevolutionary models

We applied a model-fitting approach to characterize patterns of body size evolution in Crocodylomorpha, using a set of uniform and non-uniform evolutionary models. Four uniform models were selected. First, a uniform Brownian motion (BM model), which describes diffusive, unconstrained evolution via random walks along independent phylogenetic lineages, resulting in no directional trend in trait mean, but with increasing trait variance (=disparity) through time [62, 67–69]. Second, the “early burst” (EB model; also known as “ACDC model” [128]), in which the lineages experience an initial maximum in evolutionary rate of change, that decreases exponentially through time according to the parameter *r* [129]. This results in a rapid early increase in trait variance followed by deceleration [128, 129]. Third, a uniform “trend” model, in which the parameter μ is incorporated into the BM model to describe directional multi-lineage increase or decrease in trait values through time in the entire clade [67, 68, 81].

The fourth uniform model used was the Ornstein-Uhlenbeck (OU) model, which assumes evolution under an OU process [33, 63, 66, 69]. The first formulation of an OU-based model was proposed by Hansen [63], based on Felsenstein’s [82] suggestion of using the Ornstein-Uhlenbeck (OU) process as a basis for comparative studies [66, 83]. OU-based models (also known as “Hansen” models) express the dynamics of a quantitative trait evolving along the branches of a phylogeny as the result of stochastic variation around a trait “optimum” (expressed as theta: θ), towards which trait values are deterministically attracted (the strength of attraction is given by alpha: α). The constant σ^2^, describes the stochastic spread of the trait values over time (i.e., under a Brownian motion process). Accordingly, the OU model can be formulated as:

$$ dX(t)=\upalpha\ \left[\uptheta -X(t)\right]\  dt+\upsigma dB(t) $$

This equation expresses the amount of change in trait *X* during the infinitesimal time interval from *t* to *t* + *dt*. As expressed above, the formulation includes a term describing trait attraction towards θ, which is the product of α and the difference between *X*(*t*) and θ. The term σ*dB*(*t*) describes stochastic evolution in the form of Brownian motion (BM), with random variables of mean zero and variance of *d*t (thus, σ^2^ is the rate of stochastic evolution). In this sense, if α is zero, the attraction term becomes zero, and the result is evolution by BM as a special case of OU [33, 66, 69]. The OU model can also simulate trait evolution patterns similar to that observed under other evolutionary models, such as BM with a trend incorporated, and “white noise” or stasis [33, 63, 69]. Thus, examination of the fitted parameters of the OU model is crucial for interpreting the mode of evolution [58, 61]. For example, the estimated ancestral trait value (i.e., the value of θ at the root of the tree) is given by the parameter Z_0_. Also, by obtaining ln (2)/α, we are calculating the time taken for a new macroevolutionary regime to become more influential than the ancestral regime (i.e., how long it takes to θ to be more influential than Z_0_). This parameter is often called the phylogenetic half-life (or t_0.5_) [63].

Apart from these four uniform models (i.e., BM, EB, trend and OU), we also fitted non-uniform models to our data and phylogeny. The first one is SURFACE, a non-uniform OU-based algorithm/model that allows shifts in trait optima (θ) among macroevolutionary regimes. Following the proposition of a uniform OU model, other methods attempted to model adaptive evolution under the framework of a non-uniform OU process (e.g., [78, 83, 130]). The SURFACE algorithm [88] has the advantage of automatically detecting regime shifts, which does not require a priori assumptions on where those shifts are located in the phylogeny. SURFACE identifies regime shifts using stepwise AICc (Akaike’s information criterion for finite sample sizes [89, 131, 132]), with a forward phase (that searches for all regime shifts in the phylogeny) and a backward phase (in which improvements of AICc scores merge similar regimes, detecting “convergent” evolution). Although it allows θ to vary among regimes, SURFACE assumes fixed whole-tree values of σ^2^ and α [88].

We also fitted non-uniform (multi-regime) trend-like models. Non-uniform “trend” models allow for shifts in the parameter μ, which can be explored in two different ways according to the non-uniform trend models formulated by G. Hunt and presented in Benson et al. [33]: temporal shifts in μ across all contemporaneous lineage (“time-shift trend models”), or shifts at specific nodes of the tree, modifying μ in the descendent clade (“node-shift trend models”). In time-shift trend models, shifts to a new value of μ occurs at time-horizons and are applied to all lineages alive at that time. In node-shift trend models, values of μ can vary among contemporaneous lineages. In a similar approach to the forward phase of SURFACE, the shifts in these non-uniform trend-like models are detected via stepwise AICc. In both time-shift and node-shift models, the Brownian variance (σ^2^) is constant across all regimes [33]. For our macroevolutionary analyses with the entire crocodylomorph phylogeny, we fitted trend-like models that allowed up to three time-shifts and 10 node-shifts to occur, given that analyses with more shifts are computationally intensive and often receive significantly weaker support (following results presented by Benson et al. [33]).

### Initial model comparison

Our initial model comparison involved a set of exploratory analyses to test which evolutionary models (BM, EB, OU, SURFACE and trend-like models) offered the best explanation to our data, using log-transformed cranial measurements (for both DCL and ODCL). To reduce computational demands, we used only one position of Thalattosuchia (i.e., with the group positioned within Neosuchia). The aim here was to compare the performance of uniform and non-uniform models, but also to evaluate possible influences of the different time-scaling methods (we used four different approaches as sensitivity analyses) and body size proxies. Maximum-likelihood was employed to fit these models to our body size data and the phylogeny of Crocodylomorpha, and we compared the AICc scores of each model.

### Appraisal of spurious model support

Previous works suggested caution when fitting OU models in comparative analyses, since intrinsic difficulties during maximum-likelihood fits can lead to false positives and spurious support to overly complex models (e.g., [133, 134]). This issue may be reduced when using non-ultrametric trees (as done here), as it improves identifiability of the parameters of OU models [69, 133]. We also addressed this by using the phylogenetic Bayesian information criterion (pBIC: proposed by Khabbazian et al. [77]) during the backward-phase of model implementation in all our SURFACE analyses (using the R codes from Benson et al. [33]). The pBIC criterion is more conservative than AICc, in principle favouring simpler models with fewer regimes with lower rates of false positive identification of regime shifts. Although SURFACE models were fit using pBIC, the initial model comparison described above (i.e. comparison between BM, EB, OU, SURFACE and trend-like models) used AICc scores instead, since pBIC is not yet implemented for these other models of trait evolution.

Furthermore, to evaluate the influence of spurious support for complex OU models, we simulated data under BM, once on each of our 20 phylogenies, using the parameter estimates obtained from the BM model fits to those phylogenies. We then fitted both BM and SURFACE models to the data simulated under BM, and compared several aspects of the results to those obtained from analysis of our empirical body size data (using the ODCL dataset). Specifically, we compared delta-AICc (i.e., the difference between AICc scores received by BM and SURFACE models for each tree), the number of regime shifts obtained by SURFACE, and the values of α obtained by SURFACE. This allowed us to assess whether the results of SURFACE analyses with our empirical data could be explained by overfitting of a highly-parameterised non-uniform model to data that could equally be explained by an essentially uniform process.

### Further SURFACE analyses

We initially considered both uniform and non-uniform models as equally-viable explanations of the data. However, our initial model comparisons provided strong support for the SURFACE model (see the “Results” section). Subsequent analyses therefore focussed on SURFACE, which is particularly useful because it identifies macroevolutionary regimes that provide a simplified map of the major patterns of body size evolution in crocodylomorphs. This second phase of analyses made use of all three alternative phylogenetic scenarios (varying the position of Thalattosuchia) to test the influence of phylogeny in interpretations of evolutionary regimes for body size in Crocodylomorpha. We fitted SURFACE to 20 FBD trees of each alternative topology, using body size data from the ODCL dataset (our initial model comparisons indicated that both our size indices yielded essentially identical results, and ODCL is available for more taxa; see the “Results” section). As mentioned, we performed our SURFACE analyses using pBIC [77] during the backward-phase of the algorithm.

### Clade-specific analyses with Notosuchia and Crocodylia

Two well-recognized crocodylomorph subclades, Notosuchia and Crocodylia, returned a relatively high frequency of internal macroevolutionary regime shifts, suggesting an apparently more complex evolutionary history in terms of body size. However, the SURFACE algorithm fits a single value of α to all regimes, and therefore could overestimate the strength of evolutionary constraint within regimes, and consequently miscalculate the number of distinct regimes within clades showing more relaxed patterns of trait evolution. We investigated this possibility by fitting the initial set of evolutionary models (BM, EB, OU, SURFACE and trend-like models) to the phylogenies of these two subclades (using 50 FBD trees for each clade, sampled from the posterior distribution of trees time-scaled with the FBD method) and their body size data (using only the ODCL dataset, since it includes more species). Differently from what was done for the entire crocodylomorph phylogeny, for Notosuchia we fitted trend-like models with up to 2 time-shifts and 5 node-shifts, whereas for Crocodylia we allowed up to 3 time-shifts and 7 node-shifts to occur, given that these two clades include fewer species (70 crocodylians and 34 notosuchians were sampled in our ODCL dataset) and fewer shifts are expected.

In addition, for these same clades, we also employed the OUwie algorithm [83], fitting different BM and OU-based models, which allow all key parameters to vary freely. However, differently from SURFACE, OUwie needs a priori information on the location of regime shifts in order to be implemented. Thus, we incorporated the regime shifts identified by SURFACE into our phylogenetic and body size data (by extracting, for each tree, the regime shifts from previous SURFACE results) to fit four additional evolutionary models using the OUwie algorithm: BMS, which is a multi-regime BM model that allows the rate parameter σ^2^ to vary; OUMV, a multi-regime OU-based model that allows σ^2^ and the trait optimum θ to vary; OUMA, also a multi-regime OU model, in which θ and the constraint parameter α can vary; and OUMVA, in which all three parameters (θ, α and σ^2^) can vary. Since computing all these parameter estimates can be an intensively demanding task [83], some of the model fits returned nonsensical values and were, therefore, discarded. Nonsensical values were identified by searching for extremely disparate parameter estimates, among all 50 model fits (e.g., some model fits found σ^2^ values higher than 100,000,000 and α lower than 0.00000001).

All macroevolutionary analyses were performed in R version 3.5.1 [127]. Macroevolutionary models BM, trend, EB, and OU with a single regime were fitted using the R package *geiger* [130]. The SURFACE model fits were performed with package *surface* [88]. Implementation of pBIC functions in the backward-phase of SURFACE model fits, as well as the functions for fitting non-uniform trend-like models, were possible with scripts presented by Benson et al. [33]. Simulated data under BM (for assessing the possibility of spurious support to the SURFACE model) was obtained with package *mvMORPH* [135]. The additional clade-specific model-fitting analyses, using the OUwie algorithm, were implemented with the package *OUwie* [136].

### Correlation with abiotic and biotic factors

To test whether abiotic environmental factors could be driving the evolution and distribution of body sizes in crocodylomorphs, we extracted environmental information from the literature. As a proxy for palaeotemperature, we used δ^18^O data from two different sources. The dataset from Zachos et al. [137] assembles benthic foraminifera isotopic values from the Late Cretaceous (Maastrichtian) to the Recent. The work of Prokoph et al. [138] compiled sea surface isotopic values from a range of marine organisms. Their dataset is divided into subsets representing palaeolatitudinal bands. For our analyses, we used the temperate palaeolatitudinal subset, which extends from the Jurassic to the Recent, but also the tropical palaeolatitudinal subset, which extends back to the Cambrian. For the correlation analyses, we used 10 Myr time bins (see Additional file 1 for information on time bins), by taking the time-weighted mean δ^18^O for data points that fall within each time bin. For the body size data used in the correlation tests, we calculated maximum and mean size values for each time bin, using both DCL and ODCL datasets. Correlations between our body size data and the proxies for palaeotemperature were first assessed using ordinary least squares (OLS) regressions. Then, to avoid potential inflation of correlation coefficients created by temporal autocorrelation (the correlation of a variable with itself through successive data points), we used generalised least squares (GLS) regressions with a first-order autoregressive model incorporated (see e.g., [38, 139–141]). Furthermore, to test the possible differential influence of temperature on marine versus continental (terrestrial and freshwater) animals, we also created two additional subsets of our data, one with only marine and another with only non-marine crocodylomorphs (ecological information for each taxon was obtained primarily from the literature (e.g., [38, 142]), but also from the PBDB).

We also collected palaeolatitudinal data for every specimen in our dataset from the Paleobiology Database (PBDB) and the literature, and tested the correlation between these and our body size data (DCL and ODCL datasets). To test whether our body size data is correlated with palaeolatitudinal data, we first applied OLS regressions to untransformed data. Then, to deal with possible biases generated by phylogenetic dependency, we used phylogenetic generalized least squares regressions (PGLS [143]), incorporating the phylogenetic information from the maximum clade credibility (MMC) tree, with Thalattosuchia placed within Neosuchia, obtained from our MCMC tip-dating results. For this, branch length transformations were optimised between bounds using maximum-likelihood using Pagel’s λ [144] (i.e., argument λ = “ML” within in the function pgls() of the R package *caper* [145]). As for the correlation analyses between our body size data and palaeotemperature, we also analysed marine and only non-marine taxa separately. To explore the effects of these two abiotic factors on the distribution of body sizes at more restricted levels (temporal and phylogenetically), we repeated our correlation analyses with abiotic factors (both palaeotemperature and palaeolatitude) using subsets of both ODCL and DCL datasets, including body size data only for species of Crocodylia, Notosuchia, Thalattosuchia, and Tethysuchia. For crocodylians, correlations with paleotemperature were restricted to the Maastrichtian until the Recent (i.e., data from [137]).

We also explored the possible impact of clade-specific evolutionary transitions between the environments on crocodylomorph body size evolution. For that, we assigned each taxon to a different lifestyle/ecological category using primarily the literature (e.g., [38, 142]), but further inspecting this information with the PBDB. This allowed us to subdivide our body size data (from the ODCL dataset, since it included more taxa) into three discrete categories to represent different generalised ecological lifestyles: terrestrial, semi-aquatic/freshwater, and aquatic/marine. We then used analysis of variance (ANOVA) for pairwise comparisons between different lifestyles. We also accounted for phylogenetic dependency by applying a phylogenetic ANOVA [146], incorporating information from the MCC tree with Thalattosuchia placed within Neosuchia. For both ANOVA and phylogenetic ANOVA, Bonferroni-corrected *p*-values (*q*-values) for *post-hoc* pairwise comparisons were calculated. Phylogenetic ANOVA was performed with 100,000 simulations.

All correlation analyses (with abiotic and biotic factors) used log-transformed cranial measurements (DCL or ODCL) in millimetres and were performed in R version 3.5.1 [127]. GLS regressions with an autoregressive model were carried out using the package *nlme* [147], PGLS regressions used the package *caper* [145], whereas phylogenetic ANOVA was performed using the package *phytools* [148].

### Disparity estimation

Important aspects of crocodylomorph body size evolution can be revealed by calculating body size disparity through time. There are different methods and metrics for quantifying morphological disparity (e.g., [148–152]), and in the present study disparity is represented by the standard deviation of log-transformed body size values included in each time bin. We also plotted minimum and maximum sizes for comparison. Our time series of disparity used the same time bins as for the correlation analyses (with palaeotemperature), with the difference that only time bins with more than three taxa were used for calculating disparity (time bins containing three or fewer taxa were lumped to adjacent time bins; see Additional file 1 for information on time bins). Disparity through time was estimated in R version 3.5.1 [127], based on the ODCL dataset (since it includes more taxa).

## Results

### Initial model comparison

**Fig. 2:**
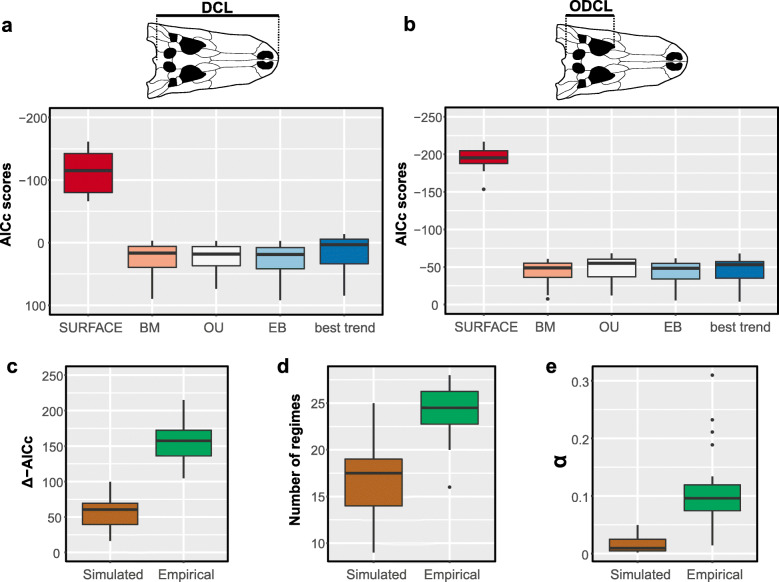
**a** and **b** Boxplots showing AICc scores of the evolutionary models fitted to crocodylomorph phylogeny and body size data (using 20 trees time-calibrated with the FBD method). Results shown for two cranial measurements datasets: ODCL (**a**) and DCL (**b**), with silhouettes of crocodylomorph skulls to illustrate the respective measurement (following [96]). For the trend-like models, only the AICc of the best model (“best trend”) is shown. See Additional files 1 and 3 for further results. **c-e** Comparative results of evolutionary models fitted to simulated data (under Brownian Motion) and our empirical body size data (using the ODCL dataset). Data was simulated for 20 crocodylomorph time-scaled trees, and the same trees were used for fitting the evolutionary models. **c** Δ-AICc is the difference between AICc scores received by BM and SURFACE models. **d** Number of regime shifts detected by the SURFACE algorithm. **e** Values of α estimated by the SURFACE algorithm. Results shown for simulated and empirical data

Comparisons between the AICc scores for all the evolutionary models fitted to our crocodylomorph body size data (BM, EB, OU, SURFACE and trend-like models) show extremely strong support (i.e. lower AICc values) for the SURFACE model (Fig. 2a and b; see Additional file 1: Figure. S5 for results of the sensitivity analyses using different time-scaling methods). This is observed for both body size proxies (DCL and ODCL) and independently of the time-scaling method used. All uniform models exhibit relatively similar AICc scores, including the OU model with a single macroevolutionary regime, and all of these are poorly supported compared to the SURFACE model. For trees calibrated with the FBD methods, all trend-like models (i.e., either uniform or multi-trend models) received very similar support, using both size proxies, and have AICc values that are more comparable to the set of uniform models than those of the SURFACE model. Even the best trend-like model (usually the models with two or three node-shifts, which are shown as the “best trend” model in Fig. 1a and b) have significantly weaker support than SURFACE, regardless of the time-calibration method used (see Additional file 3 for a complete list of AICc scores, including for all trend-like models).

### Appraising spurious support to the SURFACE model

We simulated data under a BM to assess the possibility of spurious support for our SURFACE model fits. SURFACE models were generally favoured by AICc compared to the single-regime BM model under which the data were simulated, indicating the possibility of spurious support. This is consistent with previous observations of spurious support and high false positive rates for SURFACE models based on stepwise AICc methods [133, 134] even though pBIC was used to select among SURFACE models in our study. Nevertheless, substantially stronger support was found for SURFACE model fits on our empirical data when compared to those on simulated data (Fig. 2c–e). Median delta-AICc (i.e. the difference between AICc scores received by BM and SURFACE models for each tree) for the simulated data was 60.38, compared to 157.93 for the empirical data, and the distributions of delta-AICc values are significantly different according to a Wilcoxon–Mann–Whitney test (*p* <  0.001). Furthermore, the number of regime shifts detected and the values of α estimated are significantly higher (*p* <  0.001) when using the empirical data (Fig. 1c–e). The median value of α was 0.009 for the simulated data, indicating a phylogenetic half-life of 77 Myr, compared to 0.09 for our empirical data (phylogenetic half-life of 7.7 Myr). Therefore, regimes in our empirical data converge to their body size optima much more rapidly than expected under Brownian motion. Median number of regimes detected was of 17.5 for simulated data, compared to 24.5 for the empirical data.

These results suggest that the support found for SURFACE models when using our empirical data goes beyond what was anticipated if they were simply due to false positives expected for these complex, multi-regime models [133]. Furthermore, the SURFACE model fits represent a useful simplification of major patterns of body size evolution in a group, and particularly the shifts of average body sizes among clades on the phylogeny. Thus, although we acknowledge that some model fits might be suboptimal (such as those demonstrated by Benson et al. [33]) or could be returning some unrealistic parameter estimates, we use our SURFACE results to provide an overview of crocodylomorph body size evolution that is otherwise lacking from current literature.

### Describing the body size macroevolutionary patterns in Crocodylomorpha

**Fig. 3:**
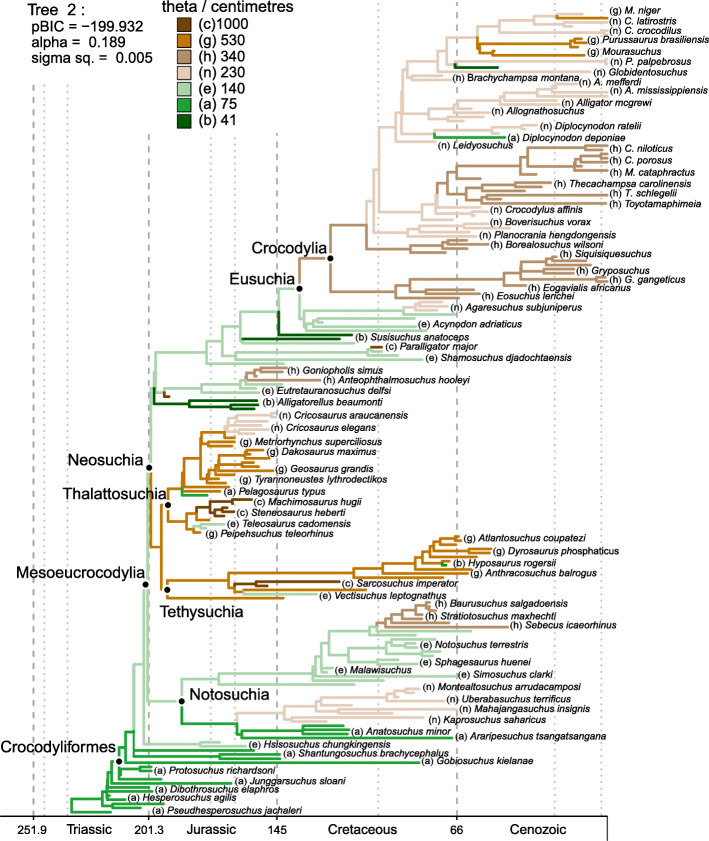
SURFACE model fit (using pBIC searches in the backward-phase) on tree number 2 among crocodylomorph topologies with Thalattosuchia placed within Neosuchia, using the ODCL dataset and time-calibrated with the FBD method. Attraction to unrealized low or high trait optima are highlighted in blue and red, respectively. Model fits of trees sharing the same position of Thalattosuchia show very similar regime configurations

**Fig. 4:**
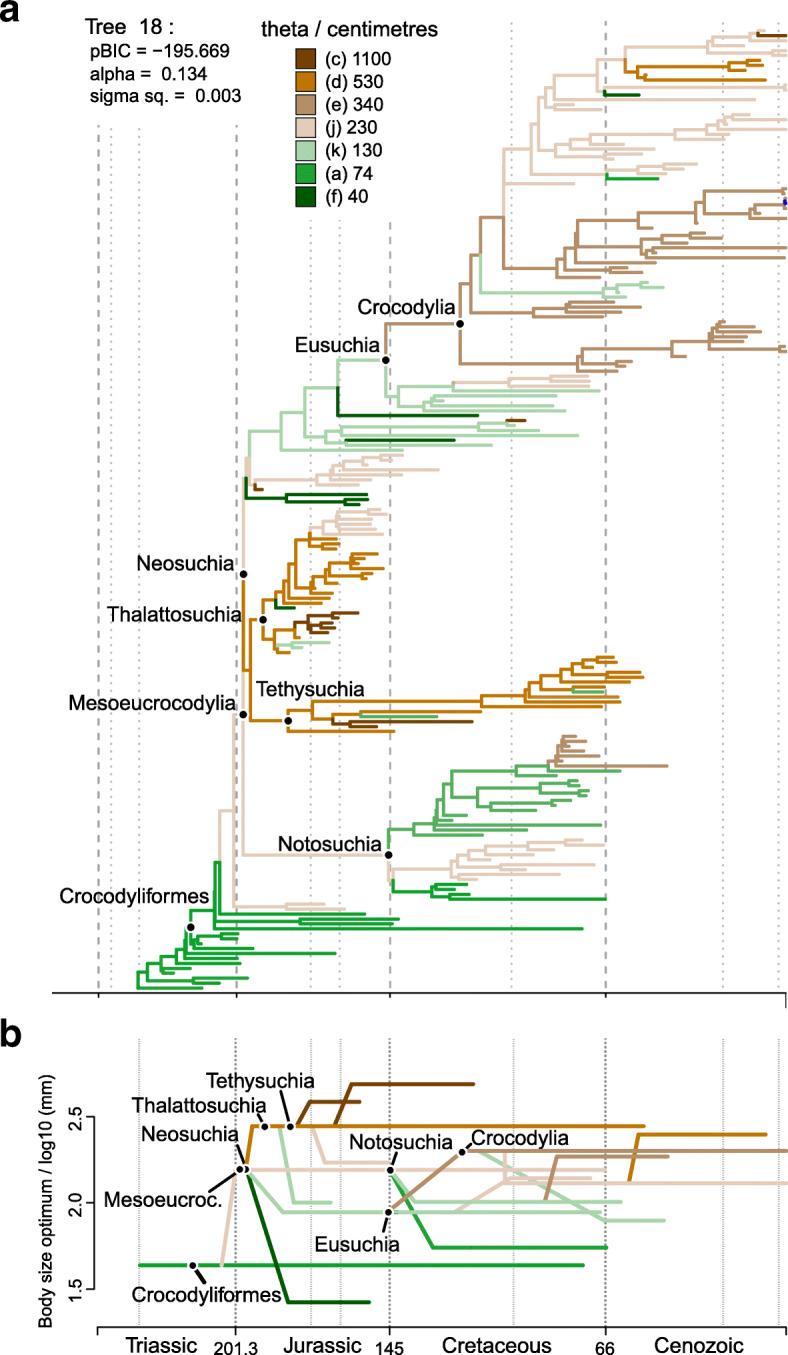
**a** SURFACE model fit (using pBIC searches in the backward-phase) on tree number 18 among crocodylomorph topologies with Thalattosuchia placed within Neosuchia, using the ODCL dataset and time-calibrated with the FBD method. Attraction to unrealized low or high trait optima are highlighted in blue and red, respectively. **b** Simplified version of **a**, with independent multi-taxon regimes collapsed to single branches

**Fig. 5:**
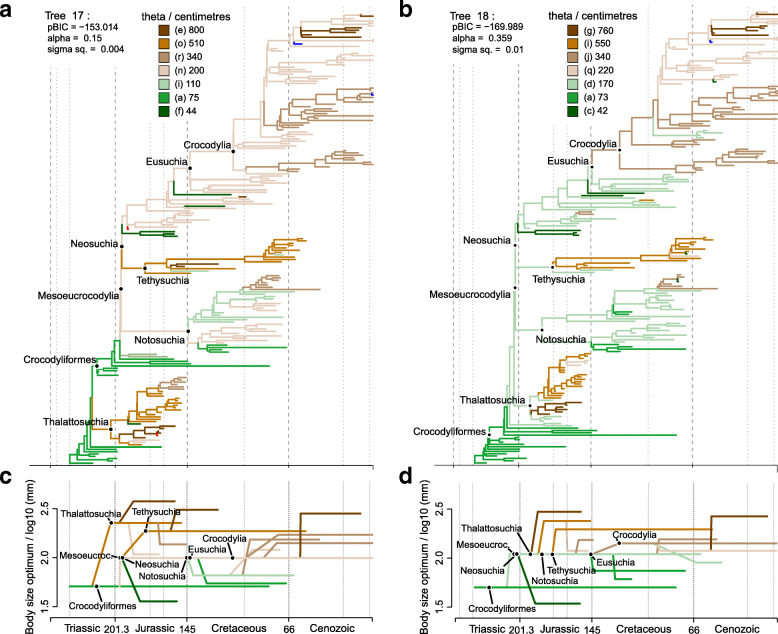
SURFACE model fits of trees time-calibrated with the FBD method, using the ODCL dataset. Attraction to unrealized low or high trait optima are highlighted in blue and red, respectively. **a** Model fit on tree number 17 with Thalattosuchia as the sister group of Crocodyliformes. Some model fits of trees sharing this same position of Thalattosuchia show simpler model configurations, with significantly fewer regimes (see text for details and Additional file 4 for all SURFACE plots). **b** Model fit on tree number 18 with Thalattosuchia as the sister group of Mesoeucrocodylia. **c** and **d** Simplified versions of **a** and **b**, respectively, with independent multi-taxon regimes collapsed to single branches

The use of alternative positions of Thalattosuchia (see the “Methods” section) allowed us to further examine the impact of more significant changes to tree topologies on our SURFACE results. In general, similar model configurations were found for all tree topologies (Figs. 3, 4, and 5; see Additional file 4 for all SURFACE plots), with numerous regime shifts detected along crocodylomorph phylogeny. However, simpler model fits (i.e., with significantly less regime shifts) are relatively more frequent when Thalattosuchia is placed as the sister group of Crocodyliformes. To further investigate this, we recalibrated the same tree topologies with other time-scaling methods (i.e., *mbl* and *cal3* methods), and applied SURFACE to those recalibrated trees. Some of these trees returned more complex models, with a greater number of regime shifts and better pBIC scores. This indicates that some of the simpler model configurations might be suboptimal, given that AIC procedures might face difficulties [153], which have previously demonstrated for other datasets (e.g., in dinosaurs [33]).

Overall, most SURFACE model fits identified more than five main macroevolutionary regimes (i.e., “convergent” regimes, identified during the backward-phase of SURFACE), independently of the position of Thalattosuchia (Figs. 3, 4, and 5). Those are distributed along crocodylomorph phylogeny by means of numerous regime shifts, usually more than 20. Trait optima values for these regimes varied significantly among different crocodylomorph subclades and are described in detail below. Overall, regime shifts are frequently detected at the bases of well-recognised clades, such as Thalattosuchia, Notosuchia and Crocodylia. Nevertheless, shifts to new regimes are not restricted to the origins of these diverse clades, since many other regime shifts are observed across crocodylomorph phylogeny, including regimes containing only a single species.

Our SURFACE results indicate an ancestral regime of small body sizes for Crocodylomorpha, regardless of the position of Thalattosuchia (Figs. 3, 4, and 5). This is consistent with the small body sizes of most non-crocodyliform crocodylomorphs such as *Litargosuchus leptorhynchus* and *Hesperosuchus agilis* [55, 56]*.* The vast majority of the model fits show trait optima for this initial regime (Z_0_) ranging from 60 to 80 cm (total body length was estimated only after the SURFACE model fits, based on the equation from [96]; see the “Methods” section). Very few or no regime shifts are observed among non-crocodyliform crocodylomorphs (Figs. 3, 4, and 5b). The possible exception to this is when Thalattosuchia is placed outside Crocodyliformes, since members of this group which occupy large body sized regimes (θ = 500–1000 cm; Fig. 5a). Regardless of the position of Thalattosuchia however, the ancestral regime of all crocodylomorphs (Z_0_) was inherited by protosuchids (such as *Protosuchus*, *Orthosuchus*, and *Edentosuchus*) and some other non-mesoeucrocodylian crocodyliforms (e.g., *Shantungosuchus*, *Fruitachampsa*, *Sichuanosuchus* and *Gobiosuchus*).

Mesoeucrocodylia and *Hsisosuchus* share a new evolutionary regime of slightly larger body sizes (θ = 130–230 cm) in most model fits. This is usually situated at the end of the Late Triassic (Rhaetian), and the recovery of this shift is independent of the phylogenetic position of Thalattosuchia (Figs. 3, 4, and 5). This regime is often inherited by Notosuchia and Neosuchia, even though many regime shifts are observed later on during the evolution of these two clades. Within Notosuchia, regime shifts to smaller sizes (θ = 60–100 cm) are often seen in uruguaysuchids (including all *Araripesuchus* species), *Anatosuchus*, *Pakasuchus* and *Malawisuchus*. Shifts towards larger sizes are seen among peirosaurids (θ = 210–230 cm) and, more conspicuously, in sebecosuchids and sometimes in the armoured sphagesaurid *Armadillosuchus arrudai* (θ = 330–350 cm).

Independent regime shifts to much smaller sizes (θ = 40–60 cm) are present among non-eusuchian neosuchians (excluding Thalattosuchia and Tethysuchia), particularly in atoposaurids, *Susisuchus*, and *Pietraroiasuchus*, whereas shifts to larger sizes (θ = 300–850 cm) are also detected, often in *Paralligator major* and in some goniopholidids. Within both Tethysuchia and Thalattosuchia, most taxa occupy a regime of relatively large body sizes (θ = 500–1000 cm). When these two clades are sister taxa to one another (Figs. 3 and 4) they usually inherit a same body size regime (θ = 500–550 cm), which originated during the Early Jurassic (Hettangian). In contrast, when Thalattosuchia is placed as sister to Crocodyliformes or Mesoeucrocodylia (Fig. 5), the regime shifts to larger sizes are often independent, and occur at the base of each clade (also with θ values around 500 cm) or later on during their evolutionary history (e.g., some model fits show Tethysuchia with regime shifts to larger sizes only at the base of Dyrosauridae [θ ≈ 500 cm] and the clade formed by *Chalawan* and *Sarcosuchus* [θ = 800–1000 cm]). Both groups also exhibit regime shifts to smaller sizes (θ = 100–150 cm) in some lineages, such as those leading to *Pelagosaurus typus* and *Teleosaurus cadomensis* within Thalattosuchia, and *Vectisuchus* within Tethysuchia. Among thalattosuchians, a conspicuous shift towards larger body sizes (θ = 800–1000 cm) is frequently observed in the teleosaurid clade formed by *Machimosaurus* and *Steneosaurus*, whereas within Metriorhynchidae, a shift to smaller sizes (θ = 230–350 cm) is often detected in Rhacheosaurini*.*

Similar to Thalattosuchia and Tethysuchia, Crocodylia is another group characterized by a predominance of macroevolutionary regimes of relatively large sizes. Indeed, regimes of larges sizes are frequently associated with clades of predominantly aquatic or semi-aquatic crocodylomorphs, although not strictly restricted to them. Regarding Crocodylia, a Cretaceous regime shift is usually detected at the base of the clade (Figs. 3, 4, and 5), changing from the macroevolutionary regime of smaller sizes (θ = 130–180 cm) found for closely related non-crocodylian eusuchians (such as hylaeochampsids and some allodaposuchids) to a regime of larger trait optimum (θ = 280–340 cm). When this is the case, this same ancestral regime to all crocodylians is inherited by many members of the clade, particularly within Crocodyloidea and Gavialoidea. However, some model fits show Crocodylia inheriting the same regime as closely related non-crocodylian eusuchians, more frequently when Thalattosuchia is placed outside Neosuchia. In these cases, shifts towards larger body sizes are still seen in members of Crocodyloidea and Gavialoidea, but they only occur later in time and arise independently (Fig. 5a). In comparison to the other two main lineages of Crocodylia, Alligatoroidea is characterized by a regime of lower trait optima values (θ = 210–230 cm), which frequently occurs as a Late Cretaceous shift at the base of the clade. But Alligatoroidea is also distinct from the other two clades by exhibiting more regime shifts, reflecting its great ecological diversity and body size disparity (ranging from very small taxa, such as the caimanine *Tsoabichi greenriverensis*, to the huge *Purussaurus* and *Mourasuchus*).

### Modes of body size evolution within Notosuchia and Crocodylia

The significant number of regime shifts that occur within both Notosuchia and Crocodylia led us to more deeply scrutinise the modes of body size evolution in these two clades. We therefore conducted another round of model-fitting analyses, initially fitting the same evolutionary models (SURFACE, OU, BM, EB and trend-like models) to subtrees representing both groups. In addition, we used the same regime shifts identified by the SURFACE algorithm to fit four additional models using the OUwie algorithm (BMS, OUMV, OUMA and OUMVA), which allow more parameters to vary, but need regime shifts to be set a priori.

**Fig. 6:**
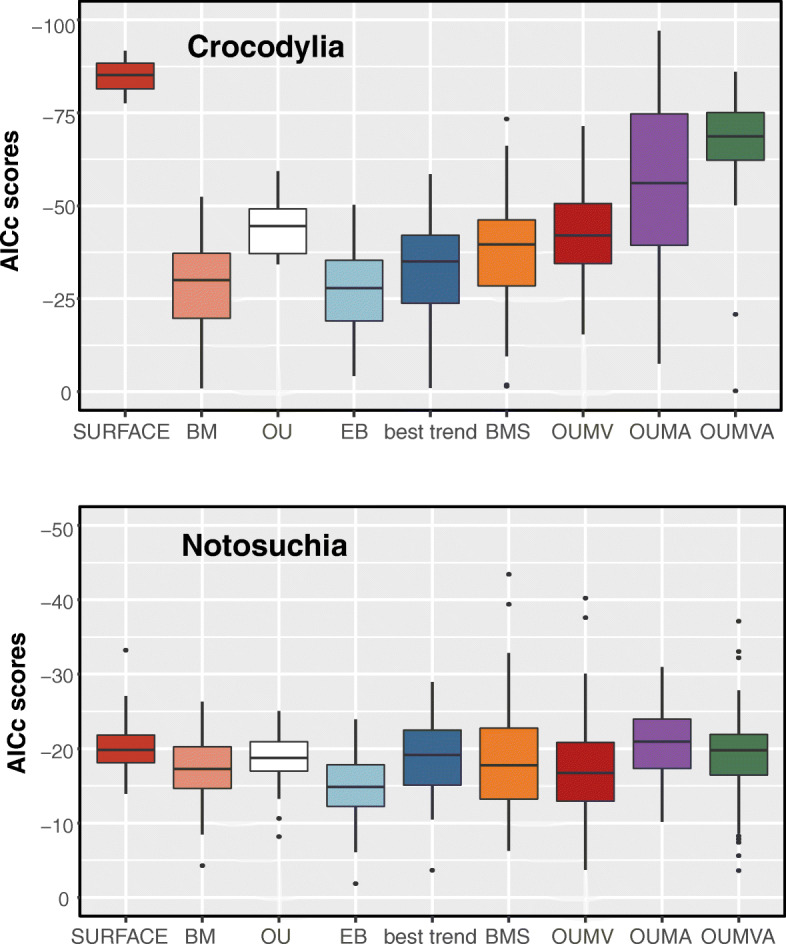
AICc scores of all evolutionary models fitted to the phylogenies and body size data of Crocodylia (top) and Notosuchia (bottom). For the trend-like models, only the AICc of the best model (“best trend”) is shown

The results of these analyses indicate different modes of body size evolution during the evolutionary histories of these two groups. In Crocodylia (Fig. 6; see Additional file 3 for a complete list of AICc scores), AICc scores indicate a clear preference for OU-based models, with highest support found for the SURFACE model, but also strong support for the uniform OU model, as well as OUMA and OUMVA models. The SURFACE algorithm frequently identified at least three main (i.e. “convergent”) macroevolutionary regimes for crocodylians (with θ values around 200, 350 and 750 cm), usually with α ranging from 0.02 to 0.2 and σ^2^ between 0.0007 and 0.02. When allowed to vary among regimes (i.e., in models OUMA and OUMVA), ranges of both parameters increase significantly, with some model fits displaying extremely unrealistic parameter values, which might explain the stronger support found for SURFACE compared to these latter models. Even though the relatively small number of taxa included in these analyses (i.e. *N* = 70) suggests caution when interpreting the higher support for OU-based models [134], BM-based models received consistently worse support than any of the four OU-based models mentioned above, even the best trend-like model (usually the one with the best AICc scores among BM-based models).

Our results show a different scenario for Notosuchia, for which we found comparable support for all evolutionary models analysed (Fig. 6). Among OU-based models, slightly better AICc scores were found for the SURFACE model. However, this model received virtually the same support as the BMS model, the best of the BM-based models. BMS is a multi-regime BM model that allows the rate parameter (σ^2^) to vary, and, as α is effectively set to zero, represents diffusive model of evolution. The support found for this model might suggest a more relaxed mode of body size evolution in notosuchians, which is consistent with the wide range of body sizes observed in the group, even among closely-related taxa. Although OU-based models (including SURFACE) are not favoured over other evolutionary models, we can use some SURFACE model to further explore body size evolutionary patterns among Notosuchia. For example, even though we sampled twice as many crocodylians (*N* = 70) as notosuchians (*N* = 34), many SURFACE model fits found three main macroevolutionary regimes for notosuchians, similar to what was found for Crocodylia (although model fits with less regimes were more frequent for Notosuchia than Crocodylia). For these, θ values were usually around 80, 150 and 320 cm, with α usually ranging from 0.008 to 0.05 and σ^2^ between 0.0007 and 0.005. When the same regimes detected by the SURFACE algorithm were used by the OUwie algorithm to fit the BMS model, values of σ^2^ rarely varied significantly from the range of whole-tree σ^2^ estimated for the SURFACE model fits. The few exceptions were usually related to regimes with unrealised θ values, as in the case of the armoured sphagesaurid *Armadillosuchus arrudai* (probably with more than 2 m in total length*,* whereas other sampled sphagesaurids would reach no more than 1.2 m [154]), and sebecosuchians (top predators of usually more than 2.5 m [102]), even though these values might still be realistic when simulating trend-like dynamics (i.e., in a single lineage with extremely disparate trait values [19, 62]).

It is worth mentioning that alternative phylogenetic scenarios proposed for Crocodylia (such as the position of gavialids in relation to tomistomines and “thoracosaurs” [155]) and Notosuchia (such as the position of sebecids in relation to baurusuchids and peirosaurids [109, 111, 156]) could potentially have an influence on the regime shift detection performed by SURFACE, given the algorithm sensitivity to changes in branch lengths. Nevertheless, we do not have enough evidence to conclude that this would imply in significant changes in model support, given that we did not sample a substantial number of taxa for these groups (i.e., 8 gavialids, 3 “thoracosaurs”, and only one sebecid). An example R script with the model-fitting macroevolutionary analyses performed here, as well as the (unscaled) phylogenetic trees, can be found within Additional files 5 and 6.

### The influence of palaeolatitude and palaeotemperature

**Fig. 7:**
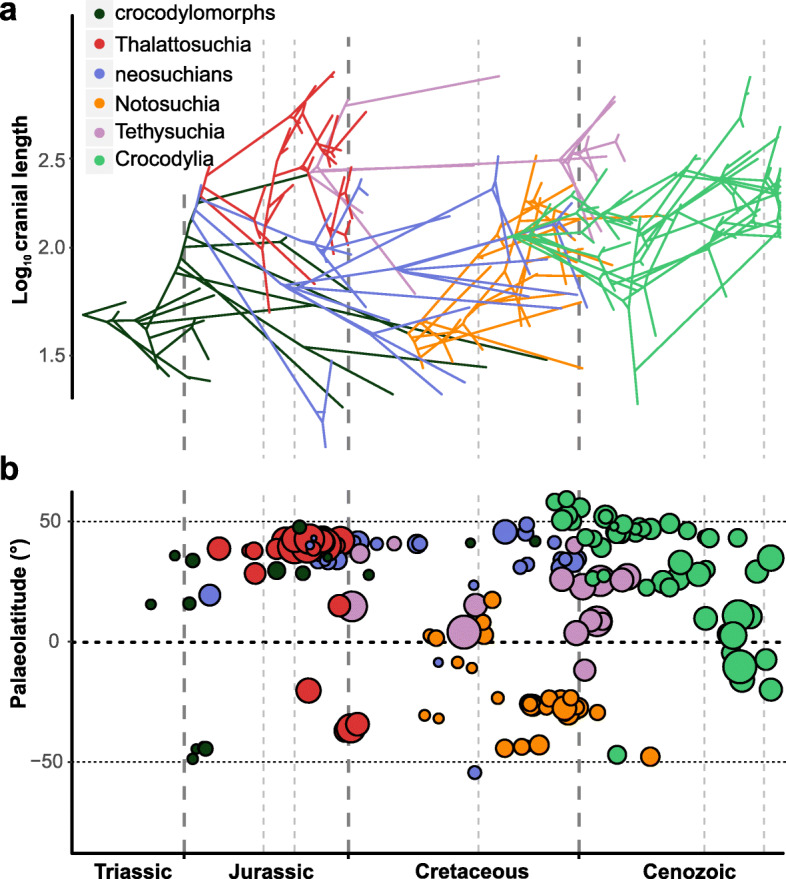
Crocodylomorph body size through time, with colours representing different mono- or paraphyletic (i.e., crocodylomorphs = non-mesoeucrocodylian crocodylomorphs, excluding Thalattosuchia; neosuchians = non-crocodylian neosuchians) crocodylomorph groups. Body size represented by log_10_ ODCL (orbito-cranial dorsal length) in millimetres. **a** Phenogram with body size incorporated into crocodylomorph phylogeny. **b** Palaeolatitudinal distribution of extinct crocodylomorphs through time, incorporating body size information (i.e., different-sized circles represent variation in body size)

Most of the correlation analyses between our body size data and the different datasets of the abiotic factors palaeotemperature and palaeolatitude yielded weak (coefficient of determination R^2^ usually smaller than 0.2) or non-significant correlations (see Additional file 1 for all regressions and further results). This is consistent with the distribution of crocodylomorph body size through time (Fig. 7), as well as with the results from our macroevolutionary analyses, which found strong support for a multi-regime OU model (SURFACE). This suggests that shifts between macroevolutionary regimes (which we interpret as “maximum adaptive zones” sensu Stanley [11]) are more important in determining large-scale macroevolutionary patterns of crocodylomorph body size evolution than these abiotic factors, at least when analysed separately.

However, one important exception was found: a correlation between mean body size values and palaeotemperatures from the Late Cretaceous (Maastrichtian) to the Recent (data from [137]). Using either all taxa in the datasets or only non-marine species, we found moderately strong correlations (R^2^ ranging from 0.376 to 0.635), with higher mean body size values found in time intervals with lower temperatures (i.e., positive slopes, given that the δ^18^O proxy is inversely proportional to temperature). The correlation was present even when we applied GLS regressions with an autoregressive model (Table 1), which returned near-zero or low autocorrelation coefficients (phi ranging from 0.157 to 0.014). This suggests that temperature might have had an influence in determining the body size distribution of crocodylomorphs at smaller temporal and phylogenetic scales. For this reason, we decided to further scrutinise the relationships between the distribution of body sizes and these abiotic factors at these smaller scales, repeating our regression analyses using only data for Crocodylia, Notosuchia, Thalattosuchia, and Tethysuchia (see the “Methods” section).

**Table 1:** Regression results of mean values of body size values on palaeotemperature

Dataset	GLS				OLS (untransformed)			
	Phi	Intercept	Slope	AIC	R^2^	Intercept	Slope	AIC
ODCL with all taxa	−0.046	2.022	0.055 (0.002)	−31.576	0.635	2.023	0.054 (0.003)	−33.557
DCL with all taxa	0.014	2.433	0.081 (0.011)	−19.577	0.527	2.433	0.081 (0.01)	−21.575
ODCL non-marine	−0.157	1.964	0.06 (0.007)	−24.96	0.502	1.965	0.06 (0.013)	−26.706
DCL non-marine	−0.089	2.345	0.07 (0.027)	−16.045	0.376	2.346	0.07 (0.034)	−18.272

Results of GLS (with an autoregressive model) and OLS (untransformed data) regressions. Mean body size represented by mean values of log-transformed cranial measurements (DCL and ODCL), in millimetres. Data from both ODCL and DCL datasets was divided into subsets with all crocodylomorphs or only non-marine species. *N* = 10 in all four subsets (number of time bins analysed). Palaeotemperature data from [137], represented by δ^18^O data from the Late Cretaceous to Recent. Only significant correlations (*p* <  0.05) are shown

To some extent, these additional regressions give further support to the hypothesis that at least some crocodylomorph subclades show a correspondence between body size and global palaeotemperature. Although most of the regressions provided non-significant or weak/very weak correlations (see Additional file 1 for all regression results), including all regressions of body size on palaeolatitudinal data, both maximum and mean body size values of Crocodylia at least are moderately correlated to palaeotemperature through time (Table 2). The positive slopes and coefficients of determination (R^2^ ranging from 0.554 to 0.698) indicate that the lowest temperatures are associated with the highest body size values in the crown-group. However, correlations with data from other subclades (Notosuchia, Thalattosuchia and Tethysuchia) were mostly non-significant, suggesting that this relationship between body size and temperature was not a widespread pattern among all groups.

**Table 2:** Regression results of maximum and mean crocodylian body size values on palaeotemperature

Dataset	GLS				OLS (untransformed)			
	Phi	Intercept	Slope	AIC	R^2^	Intercept	Slope	AIC
ODCL maximum size	0.19	2.133	0.121 (0.017)	−11.989	0.554	2.124	0.127 (0.008)	−13.662
ODCL mean size	−0.297	1.98	0.075 (0.0003)	−29.953	0.698	1.987	0.07 (0.001)	−31.137
DCL maximum size	−0.215	2.618	0.165 (0.001)	−10.724	0.632	2.627	0.157 (0.003)	−12.355
DCL mean size	−0.235	2.386	0.105 (0.0007)	−20.748	0.647	2.395	0.098 (0.003)	−22.325

Results of GLS (with an autoregressive model) and OLS (untransformed data) regressions. Mean and maximum body size only for members of the crown-group Crocodylia, represented by mean and maximum values of log-transformed cranial measurements (DCL and ODCL), in millimetres. *N* = 10 in all four datasets (number of time bins analysed). Palaeotemperature data from [137], represented by δ^18^O data from the Late Cretaceous to Recent. Only significant correlations (*p* <  0.05) are shown

### Correlation between body size and habitat choice

We initially found a relationship between lifestyle (i.e., terrestrial, semi-aquatic/freshwater, and aquatic/marine) and body size using ANOVA. However, a phylogenetic ANOVA [146] returned non-significant results (Table 3). Phylogenetic ANOVA asks specifically whether evolutionary habitat transitions are consistently associated with particular body size shifts as optimised on the phylogeny. This indicates that, although crocodylomorphs with more aquatic lifestyles (particularly marine species) tend to be large-bodied, the evolutionary transitions between these lifestyle categories were probably not accompanied by immediate non-random size changes. Furthermore, the smaller body sizes of some aquatic or semi-aquatic lineages (e.g., atoposaurids, *Tsoabichi* and *Pelagosaurus*) show that adaptive peaks of smaller sizes are also viable among aquatic and semi-aquatic species. This suggests that, even though there seems to be an ecological advantage for larger-sized freshwater and marine crocodylomorphs, the body size lower limit of species that belong to these lifestyle categories was comparable to that of terrestrial taxa.

**Table 3:** Pairwise comparison between body size of crocodylomorphs subdivided into three lifestyle categories

Category	Mean	Std. Deviation	Std. Error	Pairwise comparisons	*t*-value	ANOVA *q*-value	Phylo ANOVA *q*-value
Terrestrial	1.854	0.223	0.0333	Terrestrial – Freshwater	4.196	< 0.001*	1
Semi-aquatic/freshwater	2.026	0.249	0.0249	Terrestrial – Marine	8.721	< 0.001*	0.085
Aquatic/marine	2.263	0.185	0.0261	Freshwater – Marine	5.997	< 0.001*	0.412

Body size data from the ODCL dataset (log-transformed cranial measurement, in millimetres). Number of species in each category: 45 (terrestrial), 100 (semi-aquatic/freshwater), and 50 (aquatic/marine). Results from ANOVA, without accounting for phylogenetic dependency, and phylogenetic ANOVA [146] with 100,000 simulations

*Bonferroni-corrected *p*-values (*q*-values) significant at alpha = 0.05

## Discussion

### The adaptive landscape of crocodylomorph body size evolution

**Fig. 8:**
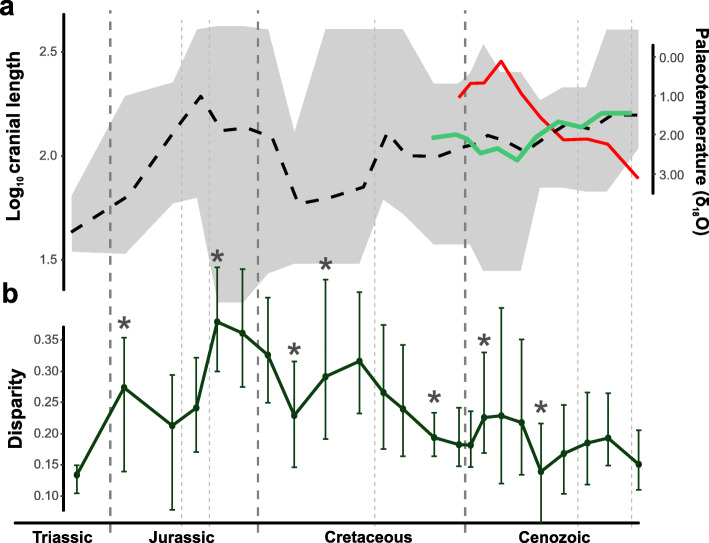
**a** Crocodylomorph body size and palaeotemperature through time. Mean log_10_ ODCL represented by dashed black line, shaded polygon shows maximum and minimum values for each time bin. Continuous light green line displays mean log_10_ ODCL values only for Crocodylia. Palaeotemperature (δ^18^O) illustrated by red line (data from [137]). **b** Body size disparity through time. Disparity is represented by the standard deviation of log_10_ ODCL values for each time bin (only time bins with more than 3 taxa were used for calculating disparity). Error bars are accelerated bias-corrected percentile limits (BCa) of disparity from 1000 bootstrapping replicates. Asterisks mark the events of largest interval-to-interval changes in disparity

Crocodylomorph body size disparity increased rapidly during the early evolution of the group, from the Late Triassic to the Early Jurassic (Hettangian–Sinemurian), which is mostly a result of the appearance of the large-bodied thalattosuchians (Fig. 8b). After a decline in the Middle Jurassic, body size disparity reaches its maximum peak in the Late Jurassic, with the appearance of atoposaurids, some of the smallest crocodylomorphs, as well as large teleosaurids (such as *Machimosaurus* [157]). This increase in disparity, which reflects skull sizes (dorsal cranial length) ranging from 106.5 to 2.3 cm (in Late Jurassic time bins), may have occurred earlier than our results suggest, given that Middle Jurassic records of atoposaurids [158] could not be included in our analyses due to their highly incomplete preservation.

Since this peak in the Middle/Late Jurassic, crocodylomorphs underwent an essentially continuous decline in body size disparity, with some short-term fluctuations related to the extinction or diversification of particular lineages (Fig. 8b). The Early Cretaceous witnessed the extinction of thalattosuchians, and a sharp decrease in disparity is seen from the Berriasian to the Barremian (although this time interval is also relatively poorly sampled in our dataset). A subsequent increase in disparity is seen in the Aptian, probably reflecting the appearance of small-bodied crocodylomorphs (such as susisuchid eusuchians). Nevertheless, this is followed by a continuing decline for the remainder of the Cretaceous (in spite of the occurrence of highly disparate notosuchians). The Cenozoic is also characterised by an overall decrease in disparity, even though some short-term increases in disparity do occur, mostly related to the presence of smaller-bodied crocodylians in the Palaeogene (such as *Tsoabichi* [159]).

We characterised the macroevolutionary patterns that gave rise to these patterns of body size disparity through time, by performing comparative model-fitting analyses. Our results indicate a strong support found for a multi-peak OU model (i.e., the SURFACE model; Fig. 2a and b). Within the concept of adaptive landscape [80, 84, 85], we can interpret the SURFACE regimes, with different trait optima, as similar to shifts to new macroevolutionary adaptive zones [11, 160]. Thus, the support found for the SURFACE model indicates that lineage-specific adaptations related to body size play an important role in determining the patterns of crocodylomorph body size evolution. Our comparative model-fitting analyses also indicate that uniform OU models, BM models, and both uniform and multi-regime trend models provide poor explanations for the overall patterns of crocodylomorph body size evolution.

Our findings reject the hypothesis of long-term, multi-lineage trends during the evolution of crocodylomorph body size. This is true even for Crocodylia, which shows increases in maximum, minimum and mean body sizes during the past 70 million years (Fig. 8a), a pattern that is classically taken as evidence for trend-like dynamics [61]. In fact, explicitly phylogenetic models of the dynamics along evolving lineages reject this.

We can also reject diffusive, unconstrained Brownian-motion like dynamics for most of Crocodylomorpha, although Notosuchia might be characterised by relatively unconstrained dynamics (Fig. 6). Single-regime (=uniform) models received poor support in general, which might be expected for long-lived and disparate clades such as Crocodylomorpha, which show complex and non-uniform patterns of body size evolution (see [5, 11, 63, 66]). Although multi-regime trend-like models received stronger support than uniform models for most phylogenies (Fig. 2a and b), multi-peak OU models (SURFACE) received overwhelmingly still greater support. This suggests that the macroevolutionary landscape of crocodylomorph body size evolution is best described by shifts between phylogenetically defined regimes that experience constrained evolution around distinct trait optima [66, 76, 80, 88].

The success of a multi-peak OU model indicates that, in general, a significant amount of crocodylomorph body size variance emerged through pulses of body size variation, and not from a gradual, BM-based dispersal of lineages through trait (body size) space. These pulses, represented by regime shifts, represent excursions of single phylogenetic lineages through body size space, resulting in the founding of new clades with distinct body size from their ancestors. This indicates that lineage-specific adaptations (such as those related to ecological diversification; see below) are an important aspect of the large-scale patterns of crocodylomorph body size evolution.

This can also explain the weak support found for the early burst (EB) model in our analyses. The early burst model attempts to simulate Simpson’s [84] idea of diversification through “invasion” of new adaptive zones (niche-filling). It focuses on a particular pattern of adaptive radiation, with evolutionary rates higher in the early evolution of a clade and decelerating through time [129]. Other models have also been proposed to better represent the concept of pulsed Simpsonian evolution (e.g., [161]). Our results show that, overall, the EB model offers a poor explanation for the evolution of body size in crocodylomorphs, in agreement with previous works that suggested that early bursts of animal body size receive little support from phylogenetic comparative methods ([129], but see [162] for intrinsic issues for detecting early bursts from extant-only datasets). However, rejection of an early burst model does not reject Simpson’s hypothesis that abrupt phenotypic shifts along evolving lineages (“quantum evolution”) results from the distribution of opportunities (adaptive zones, or unfilled niches). Patterns of crocodylomorph body size evolution could still be explained by this “niche-filling” process if opportunities were distributed through time rather than being concentrated early on the evolution of the clade. This is one possible explanation of the pattern of regime shifts returned by our analyses, and might be particularly relevant for clades with long evolutionary histories spanning many geological intervals and undergoing many episodes of radiation.

**Fig. 9:**
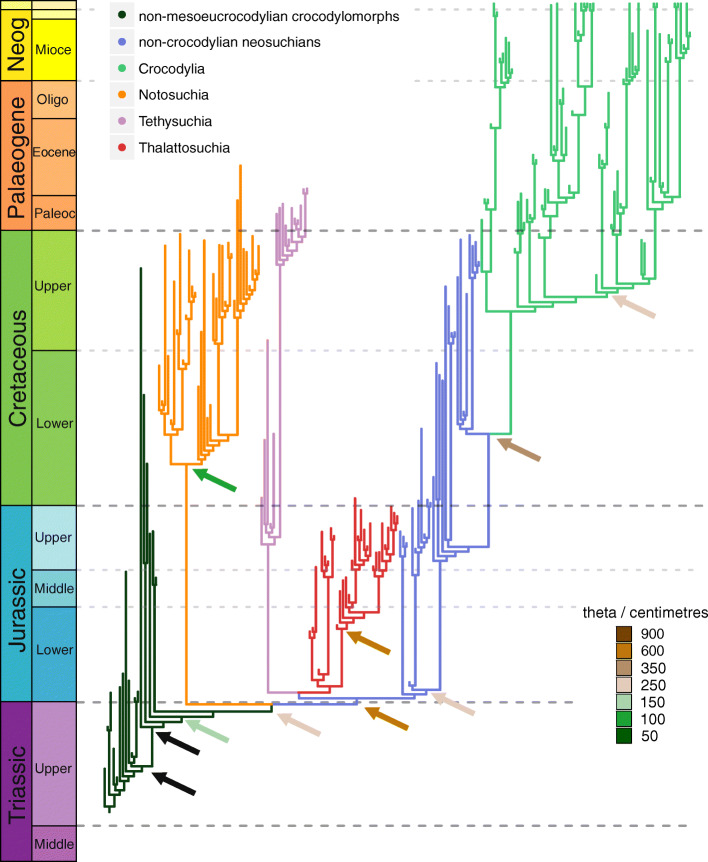
Summary of our SURFACE results combined with the crocodylomorph diversification shifts found by Bronzati et al. [37]. Nodes with diversification shifts are indicated by arrows, the colours of which represent distinct trait optima values (total body length in centimetres, after applying formula from [96]), of different body size regimes. Black arrows indicate nodes for which diversification shifts were identified, but no body size regime shift was found by any of our SURFACE model fits

Bronzati et al. [37] examined variation in rates of species diversification among clades using methods based on tree asymmetry. They found that most of crocodyliform diversity was achieved by a small number of significant diversification events that were mostly linked to the origin of some subclades, rather than via a continuous process through time. Some of the diversification shifts from Bronzati et al. [37] coincide with body size regime shifts found in many of our SURFACE model fits (such as at the base of Notosuchia, Eusuchia and Alligatoroidea; Fig. 9). However, many of the shifts in body size regimes detected by our analyses are found in less-inclusive groups (as in the case of “singleton” regimes, that contain only a single taxon).

### Ecological diversification and its implications for crocodylomorph body size distribution

**Fig. 10:**
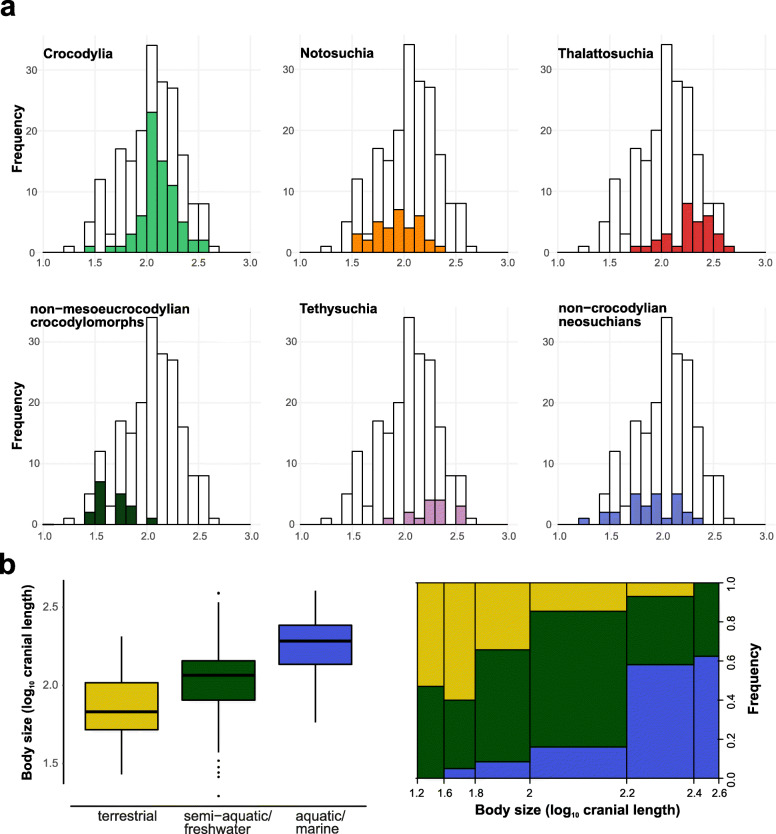
**a** Body size frequency distributions of different crocodylomorph groups (mono- or paraphyletic), constructed using the full set of 240 specimens in the ODCL dataset. Underlying unfilled bars represent values for all crocodylomorphs. Filled bars represent values for Crocodylia, Notosuchia, Thalattosuchia, non-mesoeucrocodylian crocodylomorphs (excluding thalattosuchians), Tethysuchia and non-crocodylian neosuchians (excluding tethysuchians and thalattosuchians). **b** Body size distributions of different crocodylomorph lifestyles, shown with box-and-whisker plots (on the left) and a mosaic plot (on the right). The 195 species from the ODCL dataset were subdivided into terrestrial, semi-aquatic/freshwater and aquatic/marine categories (*N* = 45, 100 and 50, respectively) based on the literature. Body size is represented by log_10_ cranial length (ODCL, orbito-cranial length, in millimetres)

Ecological factors seem to be important for the large-scale patterns of body size in crocodylomorphs. Many of the regime shifts to larger sizes detected by our SURFACE analyses occur at the base of predominantly aquatic or semi-aquatic clades, such as Thalattosuchia, Tethysuchia and Crocodylia (Figs. 3, 4, and 5), although small-bodied aquatic/semi-aquatic clades also occur, such as Atoposauridae. Some terrestrial clades also display relatively large sizes (such as sebecosuchians and peirosaurids, within Notosuchia). However, most terrestrial species are small-bodied (Fig. 10b), including many of the earliest crocodylomorphs (such as *Litargosuchus leptorhynchus* and *Hesperosuchus agilis* [55, 56]; Fig. 10a), and are within body size regimes of lower values of θ (< 150 cm; Figs. 3, 4, and 5). In contrast, the regimes with the highest values of θ (> 800 cm) are almost always associated with aquatic or semi-aquatic crocodylomorphs (e.g., the tethysuchians *Sarcosuchus imperator* and *Chalawan thailandicus* [57, 163], the thalattosuchians *Machimosaurus* and *Steneosaurus* [157, 164], and the crocodylians *Purussaurus* and *Mourasuchus* [165, 166]).

Previous studies have investigated a possible link between an aquatic/marine lifestyle and larger body sizes in other animals, particularly in mammals (e.g., [17, 21, 24]). For instance, it has been previously shown that aquatic life in mammals imposes a limit to minimum body size [24, 167] and relaxes constraints on maximum size [168]. Therefore, aquatic mammals (especially marine ones) have larger body sizes than their terrestrial relatives [21, 169]. We document a similar pattern in crocodylomorphs (Table 3), although the phylogenetic ANOVA results revealed that changes in size are not abrupt after environmental invasions (as also suggested by the diminutive size of some semiaquatic lineages, such as atoposaurids and some crocodylians). Animals lose heat faster in water than in air (given the different rates of convective heat loss in these two environments), and it has been demonstrated that thermoregulation plays an important role in determining the larger sizes of aquatic mammals [24, 167, 170]. Although mammals have distinct thermal physiology to crocodylomorphs (which are ectothermic poikilotherms), it has been reported that American alligators (*Alligator mississippiensis*) heat up more rapidly than cool down, and that larger individuals are able to maintain their inner temperature for longer than smaller ones [171]. Thus, given that both heating and cooling rates are higher in water than in air [171], larger aquatic/semi-aquatic animals could have advantages in terms of physiological thermoregulation. If extinct crocodylomorphs had similar physiologies, this could provide a plausible explanation for the larger sizes of non-terrestrial species.

### Cope’s rule cannot explain the evolution of larger sizes in Crocodylomorpha

Previous interpretations of the fossil record suggest a dominance of small sizes during the early evolution of crocodylomorphs [49, 122], inferred from the small body sizes of most early crocodylomorphs. Consistent with this, our SURFACE results revealed a small-bodied ancestral regime for Crocodylomorpha (Z_0_ between 66 and 100 cm), which was inherited virtually by all non-crocodyliform crocodylomorphs. Larger non-crocodyliform crocodylomorphs have also been reported for the Late Triassic (e.g., *Carnufex carolinensis* and *Redondavenator quayensis*, with estimated body lengths of approximately 3 m [172]), but the fragmentary nature of their specimens prevented us from including them in our macroevolutionary analysis. Nevertheless, given the larger numbers of small-bodied early crocodylomorphs, taxa like *Carnufex* and *Redondavenator* probably represent derived origins of large body size and their inclusion would likely result in similar values of ancestral trait optima (=Z_0_).

The small ancestral body size inferred for crocodylomorphs, combined with the much larger sizes seen in most extant crocodylians and in some other crocodylomorph subclades (such as thalattosuchians and tethysuchians), suggests a pattern of increasing average body size during crocodylomorph evolutionary history. This idea is reinforced by the overall increase in crocodylomorph mean body size through time, particularly after the Early Cretaceous (Fig. 8a). The same pattern also occurs within Crocodylia during the past 70 million years (green solid line in Fig. 8a), as some of the earliest taxa (such as *Tsoabichi*, *Wannaganosuchus* and *Diplocynodon deponiae*) were smaller-bodied (< 2 m) [100, 159, 173] than more recent species, such as most extant crocodylians (usually > 3 m). Cope’s rule is most frequently conceived as the occurrence of multi-lineage trends of directional evolution towards larger body sizes [7, 8, 11], and this can be evaluated using BM-based models that incorporate a directional trend (parameter μ [81]; see e.g., [33, 67]).

**Fig. 11:**
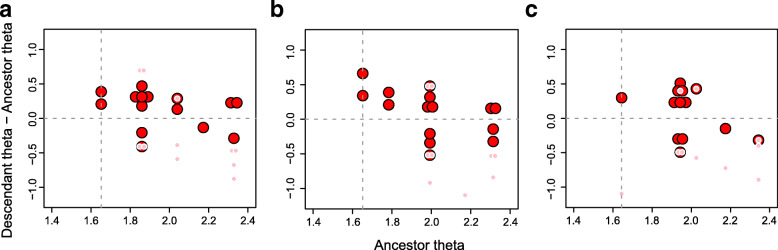
Distribution of regime shifts represented by the difference between descendant and ancestral regimes trait optima values (θ) plotted against the θ of the ancestral regime. Large red circles represent shifts that led to clades containing multiple taxa, while smaller pink circles represent “singleton” regimes, containing only a single taxon. Vertical dashed line indicates the ancestral regime for all crocodylomorphs (Z_0_), while horizontal dashed line can be used as a reference to identify regime shifts giving rise to larger (circles above the line) or smaller-bodied (circles below the line) descendants. Circles at the exact same position (i.e., shifts with the same θ values for both ancestral and descendant regimes) were slightly displaced in relation to one another to enable visualization. This plot was constructed using the θ values from trees with different positions of Thalattosuchia: **a** Tree number 2, with Thalattosuchia within Neosuchia; **b** Tree number 17, with Thalattosuchia as the sister group of Crocodyliformes; **c** Tree number 18, with Thalattosuchia as the sister group of Mesoeucrocodylia. θ values in log_10_ mm, relative to the cranial measurement ODCL (orbito-cranial dorsal length)

We find little support for trend-like models as a description of crocodylomorph or crocodylian body size evolution. Therefore, we reject the applicability of Cope’s rule to crocodylomorph evolution. This reinforces previous works suggesting that multi-lineage trends of directional body-size evolution are rare over macroevolutionary time scales [33, 72, 174, 175] (but see [19]). Furthermore, our SURFACE model fits indicate that regime shifts towards smaller-bodied descendent regimes occurred approximately as frequently (12–13 times) as shifts to regimes of larger body sizes (10–14 times; Fig. 11), when considering shifts that led to both clades containing multiple and clades containing a single taxon. Together, these results indicate that long-term increases in the average body size of crocodylomorphs also cannot be explained either by multi-lineage trends of directional evolution towards larger size, or by a biased frequency of transitions to large-bodied descendent regimes.

Instead, the apparent trend towards larger body sizes can be explained by extinctions among small-bodied regimes. Crocodylomorph body size disparity decreased gradually through the Cretaceous (Fig. 8b). This occurred due to the decreasing abundance of small-bodied species. Despite this, our SURFACE model fits mostly indicate the survival of clades exhibiting small-bodied regimes (θ < 200 cm) until approximately the end of the Mesozoic, (e.g., gobiosuchids, uruguaysuchids, sphagesaurids, hylaeochampsids and some allodaposuchids; Figs. 3, 4, and 5). Many of these small-bodied clades became extinct at least by the Cretaceous/Palaeogene (K/Pg) boundary, resulting in a substantial reduction of small-bodied species. Further reductions among the crown-group (Crocodylia) occurred by the Neogene, from which small-bodied species are absent altogether (Figs. 3, 4, and 5).

This predominance of regimes of large sizes today results from the occurrence of large body sizes in the crown-group, Crocodylia. Our SURFACE analyses focusing on Crocodylia indicate ancestral body size regimes with relatively high values of θ (Z_0_ between 220 and 350 cm). The shift to a larger-sized regime (when compared to smaller-bodied eusuchian regimes) probably occurred at the Late Cretaceous (Figs. 3, 4, and 5), and this same regime was inherited by many members of the clade (predominantly semi-aquatic species). During the Palaeogene, however, shifts to regimes of smaller sizes also occurred (such as in *Tsoabichi greenriverensis*, *Diplocynodon deponiae* and planocraniids), increasing total body size disparity (Fig. 8b). The crocodylian body size distribution shifted upwards mainly during the latter part of the Cenozoic (from the Miocene; Fig. 8b), when even larger-bodied animals occurred (e.g., *Purussaurus* and *Mourasuchus* [165, 166]), combined with the disappearance of lineages of smallest species.

### Correlation of crocodylian body size with global cooling

Our time series regressions demonstrate a moderate to strong correlation between crocodylian size and palaeotemperature (from the Late Cretaceous until the Recent; Table 2). This results from the upward-shift of the crocodylian body size distribution, coinciding with cooling global climates in the second half of the Cenozoic [137, 176]. This is an apparently counter-intuitive relationship, and we do not interpret it as a result of direct causation. Previous studies have shown that crocodylian species richness decreased with declining global temperatures of the Cenozoic [38, 39]. Furthermore, the palaeolatitudinal ranges of both marine and continental crocodylomorphs have contracted as temperatures decreased (Fig. 7b; see also [38, 39]). Therefore, the temperatures experienced by evolving lineages of crocodylians are not equivalent to global average temperatures. We propose that the association between global cooling and increasing crocodylian body size results from a systematic reduction of available habitats/niches (due to a more restricted geographical distribution), with differential extinction of smaller-bodied species. The hypothesis of selective extinction is also consistent with the decreasing in crocodylian body size disparity during the Cenozoic (Fig. 8b).

### Body size selectivity and diversification across Mesozoic boundaries

Numerous comparative studies have investigated a possible link between extinction risk and animal body size (e.g., [177–181]). For example, larger body sizes, in association with dietary specializations, might increase susceptibility to extinction in some animal groups, such as hypercarnivorous canids [182, 183]. On the other hand, the recovery of some animal clades after extinction events can also be associated with a subsequent increase in diversity and morphological disparity (e.g., Palaeogene mammals [14]), potentially leading to the exploration of new regions of body size space (i.e., invasions of new body size regimes). Thus, although for some groups (and for some extinctions) body size might play an important role, this is evidently not a generalised pattern across all animals.

For crocodylomorphs, little is known about possible influence of body size on differential extinction. Among the few studies to quantitatively investigate this, Turner & Nesbitt [49], using femoral length as a proxy for total body size, recognized a drop in mean body size of crocodylomorphs across the Triassic-Jurassic (T–J) boundary. Our SURFACE results, however, indicate otherwise: all Triassic crocodylomorphs are within a macroevolutionary regime of smaller sizes (θ < 100 cm) when Thalattosuchia is placed within Neosuchia (Figs. 3 and 4). In the other two phylogenetic scenarios, the origin of thalattosuchians (which are predominantly large-bodied animals) is placed either at the middle of the Late Triassic or closer to the T–J boundary (Fig. 5). However, as the first records of thalattosuchians only occur in the Early Jurassic, mean body size increases immediately after the boundary (Fig. 8a). The differences between our results and those found by Turner & Nesbitt [49] might be related to the distinct body size proxies used or to the different taxon sample used, as those authors also included non-crocodylomorph pseudosuchians in their analysis. In this context, we acknowledge that the inclusion in our analyses of larger non-crocodyliform crocodylomorphs, such as *Carnufex carolinensis* (~ 3 m [172]), might change our results. Apart from these differences, both Turner & Nesbitt [49] and a more a more recent study [50] found no significant influence of crocodylomorph body size on extinction at the T–J boundary, which is consistent with our analyses. Thus, at the moment we do not have empirical or statistical evidence to demonstrate selectivity of body sizes in crocodylomorphs during the end-Triassic extinction.

The Early Jurassic was characterized by key events of crocodylomorph diversification [37] and an increase in morphological disparity [45], following the end-Triassic extinction. Similarly, our body size data suggests an increase in body size disparity after the T–J boundary (Fig. 8b). Although a decrease in disparity is observed subsequently, this is probably due to the relatively few crocodylomorphs known for the latest Early Jurassic and the Middle Jurassic (Sinemurian–Aalenian [38]). Following that, the diversification of thalattosuchians during the Late Jurassic, together with the occurrence of smaller- to intermediate-bodied neosuchians (such as atoposaurids and goniopholidids), created the greatest observed disparity of crocodylomorph body sizes during their evolutionary history (Fig. 8b).

Recent studies [184–186] suggested that a combination of environmental perturbations occurred during the Jurassic-Cretaceous (J/K) transition, which might have led to the extinction of some tetrapod lineages. The boundary is characterised by a decrease in marine crocodylomorph diversity [38, 185, 186], highlighted by declines in thalattosuchian diversity, especially among teleosaurids, which suffered widespread extinction (except, apparently, at lower palaeolatitudes [187]). Nevertheless, Wilberg [46] did not find evidence for a substantial decrease in crocodylomorph cranial disparity across the J/K boundary. Similarly, our SURFACE results do not suggest dramatic changes in body size space exploration immediately before or after the J/K boundary (Figs. 3, 4, and 5), and there seems to be no defined body size selectivity across this boundary, as the multiple survivor crocodylomorph lineages were within regimes of very disparate optima values. Furthermore, the decrease in disparity observed in the middle of the Early Cretaceous (i.e., Valanginian–Barremian) is likely due to poor sampling [188], resulting in the scarcity of more completely preserved crocodylomorphs during these stages.

The Late Cretaceous is marked by a remarkable fossil richness of notosuchians, in Gondwana [189, 190], and the diversification of eusuchian crocodylians [191]. Notosuchia exhibits a wide range of body sizes (Fig. 10a), to some extent reflecting its remarkable diversity [38, 190] and morphological disparity [46, 47]. Our model-fitting analyses using only notosuchian data suggest more relaxed modes of body size evolution in Notosuchia (Fig. 6), which is consistent with their high species richness and morphological disparity. This could be explained by a combination of intrinsic (i.e., innovations and/or adaptations, such as a highly modified feeding apparatus [192, 193]) and extrinsic factors (i.e., specific environmental conditions, such as the predominantly hot and arid climate of the Gondwanan landmasses occupied by notosuchians [38, 189]).

Even though our body size data show no specific pattern at the K/Pg boundary, a decline in body size disparity is present through the Late Cretaceous, combined with an increase in mean body size (Fig. 8), a pattern that generally continued through the Cenozoic (although with some short-term fluctuations). This supports the hypothesis that the K/Pg extinction had only minor impacts on crocodylomorphs [37–39, 46, 194]. Although subsampled estimates of genus richness suggest a decline in terrestrial crocodylomorph diversity during the Late Cretaceous, this occurred prior to the K/Pg boundary, between the Campanian into the Maastrichtian, in both Europe and North America [38]. Indeed, several crocodylomorph subclades lost several species prior to the end of the Cretaceous (in particular notosuchians and non-crocodylian eusuchians [37, 38]; Figs. 3, 4, and 5), and multiple lineages within other groups, such as dyrosaurid tethysuchians and crocodylians, crossed the boundary with little change [39, 194, 195] (Figs. 3, 4, and 5). Our data suggest a long-term pattern of selective extinctions of small-bodied crocodylomorphs, starting from the Late Cretaceous and continuing to the Recent. This may have resulted from a longstanding trend of global cooling [137, 176], resulting in more restricted geographical distributions, and reducing niche availability for crocodylomorphs. This is consistent with our SURFACE results (Figs. 3, 4, and 5), that show very few smaller-bodied regimes (θ < 150 cm) during the Palaeogene and a complete absence after the Neogene. This pattern strikingly contrasts with that proposed for mammals, which may have experienced selectivity against larger bodied taxa across the K/Pg boundary [196], although an increase in body size occurred subsequently, during the Palaeogene [14, 15]. The pattern of survival in crocodylomorphs also differs from that suggested for squamates (lizards and snakes), in which small-bodied taxa show evidence of preferential survival [197].

## Conclusions

After an early increase (with the highest peak in the Late Jurassic), crocodylomorph body size disparity experienced sustained decline during virtually its entire evolutionary history. This disparity decrease is combined with an increase of average body size through time, with highest peaks in the Middle Jurassic and today. In particular, the increase in mean body size seen during the Cenozoic (mostly related to crocodylians) co-occurs with an overall decrease in global temperatures.

To further characterise these patterns, we used comparative model-fitting analyses for assessing crocodylomorph body size evolution. Our results show extremely strong support for a multi-peak Ornstein-Uhlenbeck model (SURFACE), rejecting the hypothesis of evolution based on Brownian motion dynamics (including those representing the concept of Cope’s rule). This suggests that crocodylomorph body size evolution can be described within the concept of a macroevolutionary adaptive landscape, with a significant amount of crocodylomorph body size variance evolving from pulses of body size changes, represented by shifts between macroevolutionary regimes (similar to adaptive zones or “maximum adaptive zones” of Stanley [11]). This is reflected in the regime shifts frequently detected at the base of well-recognised and diverse crocodylomorph subclades such as Notosuchia, Thalattosuchia, and Crocodylia. We find evidence for possibly more relaxed/diffusive modes of body size evolution in only one group, Notosuchia.

Overall, we did not find strong correlations between our body size data and abiotic factors, indicating that shifts between macroevolutionary regimes are more important for determining large-scale patterns of crocodylomorph body size than isolated climatic factors. However, at more refined temporal and phylogenetic scales, body size variation may track changes in climate. In the case of Crocodylia, a global cooling event might explain the long-term increases in body size, as a result of systematic reduction of available habits/niches (due to a more latitudinally-restricted geographical distribution during cooler global climates), with preferential extinction of smaller-bodied species.

Shifts towards larger sizes are often associated with aquatic/marine or semi-aquatic subclades, indicating that ecological diversification may also be relevant, and suggesting a possible link between aquatic adaptations and larger body sizes in crocodylomorphs. These shifts to larger sizes, which occurred throughout crocodylomorph evolutionary history, combined with the extinction of smaller-sized regimes (particularly during the Late Cretaceous and Cenozoic) can explain the overall increase in mean body size, as well as the large-bodied distribution of extant crocodylians (all of which are aquatic or semi-aquatic) compared to smaller-bodied early taxa.

## Additional files

Additional file 1: Supplementary methods and results. Includes additional information on: (1) proxies for total body size; (2) supertree construction; (3) alternative time-scaling methods; (4) time bins. Additional results include: (1) consensus FBD trees; (2) results of model-fitting analyses with alternative time-scaling methods; (3) correlation results (with abiotic factors). (DOCX 1572 kb)

Additional file 2: Datasets used for macroevolutionary analyses. Includes DCL and ODCL datasets, with body size data and information about taxonomy, specimen number, palaeolatitude, and lifestyle. (XLSX 59 kb)

Additional file 3: AICc scores from all model-fitting analyses. (XLSX 110 kb)

Additional file 4: ZIP-archive containing plots of all SURFACE model fits. (ZIP 1189 kb)

Additional file 5: ZIP-archive containing the (unscaled) phylogenetic trees used for our macroevolutionary analyses (in .tre format), ages data of terminal taxa (for time-scaling methods), and MrBayes command for FBD tip-dating method. (ZIP 27 kb)

Additional file 6: ZIP-archive containing R scripts and functions for running the macroevolutionary analyses in this study. (ZIP 19 kb)

## Data Availability

The data generated and/or analysed during the current study, as well as R codes used for macroevolutionary analyses and supplementary results, are included within the article and its additional files.

## Cranial measurements

DCL: Dorsal cranial length
ODCL: Orbito-cranial length

## Evolutionary models

BM: Brownian motion
BMS: Multi-regime BM model that allows parameter σ2 to vary
EB: Early burst
OU: Ornstein-Uhlenbeck
OUMA: Multi-regime OU model in which θ and α can vary
OUMV: Multi-regime OU model that allows θ and σ2 to vary
OUMVA: OU model in which all three parameters (θ, α and σ2) can vary

## Model parameters

Z_0_: Estimated trait value at the root of the tree of OU-based models
α: Attraction parameter of OU-based models
θ: Trait optimum of OU-based models
μ: Evolutionary trend parameter of BM-based models
σ^2^: Brownian variance or rate parameter of BM or OU-based models

## Optimality criteria

AIC: Akaike’s information criterion
AICc: Akaike’s information criterion for finite sample sizes
BIC: Bayesian information criterion
pBIC: Phylogenetic Bayesian information criterion
