# Supplementary material for: The multi-peak adaptive landscape of crocodylomorph body size evolution
Source: BMC Evol Biol. 2019 Aug 7;19:167. doi: 10.1186/s12862-019-1466-4 (PMC6686447; doi:10.1186/s12862-019-1466-4)
Supplement: Supplementary file 1 — Supplementary methods and results. Includes additional information on: (1) proxies for total body size; (2) supertree construction; (3) alternative time-scaling methods; (4) time bins. Additional results include: (1) consensus FBD trees; (2) results of model-fitting analyses with alternative time-scaling methods; (3) correlation results (with abiotic factors). (DOCX 1572 kb) [file 12862_2019_1466_MOESM1_ESM.docx]

**The multi-peak adaptive landscape of crocodylomorph body size evolution**

Pedro L. Godoy, Roger B. J. Benson, Mario Bronzati & Richard J. Butler

**Additional file 1**

This additional file contains:

[**Supplementary methods 1**](#_Toc6658375)

[***Proxy for total body length* 1**](#_Toc6658376)

[***Supertree construction and alternative topologies* 4**](#_Toc6658377)

[***Additional time-scaling methods* 7**](#_Toc6658378)

[***Time bins used for time series correlations and disparity calculation* 9**](#_Toc6658379)

[**Supplementary results 11**](#_Toc6658380)

[***FBD consensus trees* 11**](#_Toc6658381)

[***Initial model comparison using APT time-scaling methods* 14**](#_Toc6658382)

[***Correlations with abiotic factors* 15**](#_Toc6658383)

[***Palaeotemperature* (regression results tables) 16**](#_Toc6658384)

[***Palaeolatitude* (regression results tables) 28**](#_Toc6658385)

[**Supplementary references 33**](#_Toc6658386)

# Supplementary methods

## *Proxy for total body length*

Equations based on modern species, using either cranial (e.g., Webb & Messel, 1978; Hall & Portier, 1994; Sereno *et al*., 2001, Hurlburt *et al*., 2003; Platt *et al*., 2009; 2011) or postcranial measurements (e.g., Bustard & Singh, 1977; Farlow *et al*., 2005), have predominantly been used for estimating total body size of extinct crocodylomorph species. Although some of these approaches have been claimed to work well when applied to extinct taxa (e.g., Farlow *et al*., 2005), they are expected to be less accurate for extinct species that have different body proportions to those of extant species (e.g., Pol *et al*., 2012; Young *et al*., 2011; 2016; Godoy *et al*., 2016; but see Figure S1). An alternative approach that has been suggested is to use clade-specific equations that are derived from regressions using fossil specimens with complete skeletons preserved, such as the recently proposed equations for estimating body length in the highly specialised marine clade Thalattosuchia (Young *et al*., 2011; 2016). Nevertheless, using this approach for the entire Crocodylomorpha would require numerous different equations and, consequently, complete specimens for all desired subclades.

Furthermore, Campione & Evans (2012) demonstrated a universal scaling relationship between proximal (stylopodial) limb bone circumferences and the body masses of terrestrial tetrapods. For instance, their equations, using both femur and humerus circumference, have been applied to estimate body mass of fossil dinosaurs (e.g., Benson *et al*. 2014; 2018; Carballido *et al*., 2017). However, due to a historical neglect of crocodylomorph postcranial anatomy, especially for Mesozoic taxa (Godoy *et al*., 2016), relatively less information is available on this part of the skeleton. Based on data collected for the present study, total or partial skull lengths (i.e., complete skulls or lacking only the snouts) can be measured in fossil specimens of approximately 50% of crocodylomorph species, whereas femoral and humeral shaft circumferences or lengths can only be measured in 35% of species. This greatly reduces the number of taxa that can be sampled and limits the utility of using postcranial elements as a proxy for body size. Similar problems exist for other methods, such as the “Orthometric Linear Unit” proposed by Romer & Price (1940) that uses dorsal centrum cross section (Currie, 1978), as well as volumetric reconstructions (e.g., Colbert, 1962; Hurlburt, 1999; Motani, 2001; Bates *et al*., 2009; Sellers *et al*., 2012), since relatively complete postcranial specimens are required.

Thus, aiming for a proxy (or proxies) for total body size that could maximised sample size (for a study encompassing the entire evolutionary history of Crocodylomorpha), we decided to use two cranial measurements: total dorsal cranial length (DCL) and dorsal orbito-cranial length (ODCL), which is measured from the anterior margin of the orbit to the posterior margin of the skull. By using actual cranial measurements, rather than estimated total body length, we avoid the addition of possible errors to our model-fitting analyses (Figure S1). Furthermore, the range of body sizes among living and extinct crocodylomorphs is considerably greater than variation among size estimates for single species. Therefore, we expect to recover the most important macroevolutionary body size changes in our analyses even when using only cranial measurements.


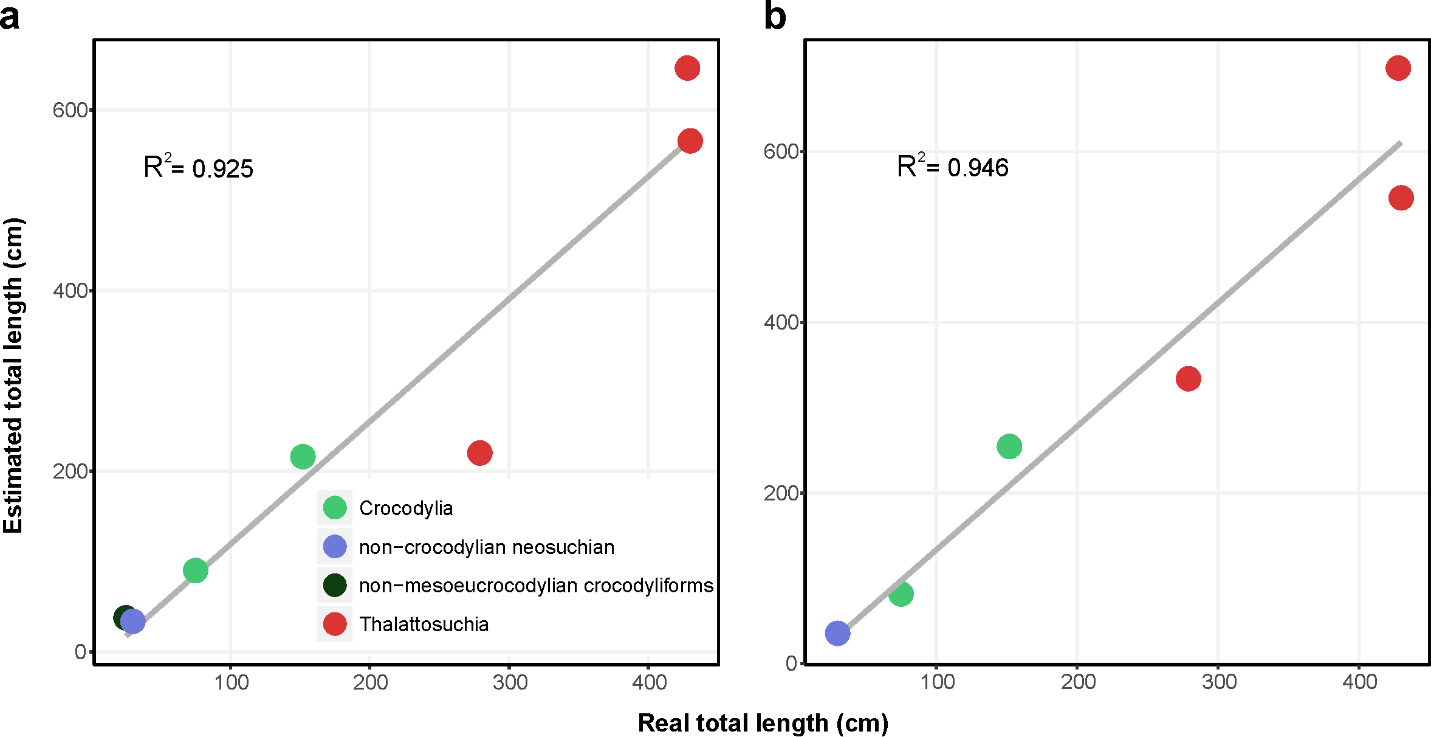


Figure S1. Expected error for total body length estimated from cranial measurements. Real total body length, measured from some complete fossil crocodylomorph specimens, is plotted against total length estimated from the cranial measurements DCL (**a**) and ODCL (**b**) (equations from Hurlburt *et al.*, 2003), exemplifying the amount of error expected when using cranial measurements to estimate total body length of crocodylomorphs. R^2^ value illustrates the strength of the correlation between real and estimated total lengths. Colours represent different mono- or paraphyletic crocodylomorph groups. See Table S1 for information on the specimens used for the construction of these plots.

Table S1. List of fossil specimens with complete skeleton preserved which data was used for creating Figure S1 of the manuscript. “DCL” is the total length estimated using the cranial measurement dorsal cranial length, “ODCL” is the total length estimated when using dorsal orbito-cranial length, and “Real TL” is the real total body length measured from the specimen. All measurements in centimetres.

| Species (specimen) | DCL | ODCL | Real TL | Source of information |
| --- | --- | --- | --- | --- |
| *Shantungosuchus chuhsienensis* (IVPP V2484) | 37.66 | N/A | 25 | First-hand observation |
| *Alligatorellus beaumonti* (BSPG 1937 I 26) | 33.77 | 35.72 | 30 | First-hand observation |
| *Diplocynodon ratelii* (MNHN.F SG 13728ab) | 216.15 | 254.74 | 152 | First-hand observation |
| *Diplocynodon darwini* (HLMD-Me 10262) | 90.02 | 81.86 | 75 | First-hand observation |
| *Platysuchus multiscrobiculatus* (SMNS 9930) | 220.22 | 334.08 | 279 | Young *et al*. (2016) |
| *Steneosaurus bollensis* (SMNS 54063) | 565.97 | 545.90 | 430 | Young *et al*. (2016) |
| *Steneosaurus leedsi* (NHMUK R 3806) | 646.58 | 698.01 | 428 | Young *et al*. (2016) |

## *Supertree construction and alternative topologies*

The supertree used as the phylogenetic framework for the macroevolutionary analyses was constructed using an informal approach. For such, we started with the MRP (matrix representations with parsimony) supertree of Bronzati *et al.* (2015), and then used some recently published phylogenetic hypotheses to create and updated version, by manually modifying the tree using the software Mesquite (Version 3.51; Maddison & Maddison, 2018). For this updated version, we added some taxa, removed others, and also changed the position of a few more, always aiming to include as many species as possible (especially the ones for which we had body size data available), but also to incorporate more well-resolved relationships from recent studies.

The supertree presented by Bronzati *et al.* (2015) is restricted to Crocodyliformes, which is less inclusive than Crocodylomorpha. Thus, we added non-crocodyliform crocodylomorphs taxa following the phylogenetic hypotheses presented by Pol *et al.* (2013) and Leardi *et al*. (2017). Within Crocodyliformes, as in Bronzati *et al.* (2015) and other recent studies (e.g., Andrade *et al.,* 2011; Montefeltro *et al.,* 2013; Pol *et al.,* 2014; Turner & Pritchard, 2015; Buscalioni, 2017), taxa classically associated to “Protosuchia” are paraphyletic arranged in relation to Mesoeucrocodylia, with smaller subgroups displayed following Bronzati *et al.* (2015) (but see below for differences in this region of the tree in the alternative topologies). Accordingly, *Hsisosuchus* is the sister-group of Mesoeucrocodylia (as in Clark, 2011, Pol *et al.,* 2014; Buscalioni, 2017) and the following groups represent taxa successively more distant to Mesoeucrocodylia: Shartegosuchidae (following Clark 2011); an unnamed clade composed by taxa such as *Sichuanosuchus* and *Shantungosuchus;* an unnamed clade composed by *Zaraasuchus* and *Gobiosuchus* (following Pol *et al.,* 2014)*;* Protosuchidae (following Clark 2011; Pol *et al.,* 2014; Turner & Pritchard, 2015).

Within Mesoeucrocodylia, Notosuchia corresponds to the sister group of all the other mesoeucrocodylians (= Neosuchia in our topology), similar to what is presented by Andrade *et al.* (2011), Pol *et al.* (2014), and Turner & Pritchard (2015). Yet, Notosuchia comprises forms such as baurusuchids, sebecosuchians, peirosaurids, sphagesaurids, uruguaysuchids, and *Araripesuchus.* The relationships among taxa within Notosuchia follow the general arrangement presented by Pol *et al.* (2014).

One of the branches at the basal split of Neosuchia leads to a clade composed by longirostrine forms, which includes Thalattosuchia and Tethysuchia (i.e. Dyrosauridae and “pholidosaurids”). Arrangement between these groups (i.e. sister-group relationship between Thalattosuchia and Tethysuchia) follows that recovered in the supertree of Bronzati *et al.* (2015). Within Tethysuchia, “pholidosaurids” are paraphyletic in relation to Dyrosauridae (also found in Pol *et al.,* 2014; Young *et al.*, 2017 and Meunier & Larsson, 2017). Relationships among Dyrosauridae follow Hastings *et al.* (2015). Relationships among thalattosuchians follow Young (2014) and Herrera *et al*. (2015).

The sister-group of the longirostrine clade mentioned above contains Eusuchia and its closest relatives such as Atoposauridae and Goniopholididae. The latter is depicted as the sister group of Eusuchia, whereas the former corresponds to the sister group of Eusuchia + Goniopholididae. This arrangement follows that recovered in Pol *et al.* (2014) and Bronzati *et al.* (2015). Regarding the internal relationships of Goniopholididae, we follow the hypotheses of Martin *et al.* (2016) and Ristevski et al. (2018). For Atoposauridae, we follow the arrangements presented by Tennant *et al*. (2016) and Schwarz *et al*. (2017). For Paralligatoridae and Susisuchidae, we followed the phylogenetic hypotheses of Turner (2015) and Turner & Pritchard (2015).

In relation to non-crocodylian eusuchians, we mainly follow the topology of Bronzati *et al.* (2015), with modifications to accommodate the arrangements proposed by Turner (2015) and Turner & Pritchard (2015) within Paralligatoridae and Susisuchidae. Regarding the interrelationships of the crown-group, as well as the position of Hylaeochampsidae + Allodaposuchidae as the sister group of Crocodylia, we follow the topology of Narváez *et al.* (2015). For the relationships within the crown-group, we follow Brochu (2012), Brochu *et al*. (2012), Scheyer *et al*. (2013) and Narváez *et al*. (2015).

Additionally, two alternative topologies were also manually constructed, for testing the impact of alternative positions of Thalattosuchia. The “longirostrine problem”, which mostly concerns the position of Thalattosuchia, has been largely debated in phylogenetic studies of Crocodylomorpha (e.g., Clark, 1994; Pol & Gasparini, 2009; Wilberg, 2015). Because of the possible impact that a group like Thalattosuchia (i.e. of relatively old origin and many species within it) can inflict in our model-fitting analyses, we built two alternative trees to test the effects related to this phylogenetic uncertainty. Apart from the position of Thalattosuchia described above (within Neosuchia), two main alternative scenarios for the position of the group within Crocodylomorpha were proposed (see Wilberg, 2015). The first places Thalattosuchia as the sister group of all other mesoeucrocodylians (= Notosuchia + Neosuchia) (e.g., Larsson & Sues, 2007; Montefeltro *et al.,* 2013), and was depicted in one of our alternative topologies. The other alternative topology places Thalattosuchia as the sister group of Crocodyliformes (following Wilberg, 2015). Only the position of Thalattosuchia has been altered in these alternative topologies. Relationships among other taxa, including the relationship among thalattosuchians, were kept as in the first topology, described above.

## *Additional time-scaling methods*

For time-calibrating our trees, apart from the Bayesian tip-dating approach, we also used three different *a posteriori* time-scaling (APT) methods: the minimum branch length (*mbl*), the *cal3* and the *extended Hedman* methods. These methods were used only for the initial model comparison.

For these methods, ages (first and last occurrence dates) were initially obtained from the Paleobiology Database, but were then checked using primary sources in the literature (see Additional file 6 for ages of all taxa). To accommodate uncertainties related to the ages of terminal taxa (i.e., most taxon ages are based on single occurrences, known only within rather imprecise bounds), we treated these first and last occurrences dates as maximum and minimum possible ages and drew terminal dates for time-calibration from a uniform distribution between these.

First, the *mbl* method (Laurin, 2004), which requires a minimum branch duration to be set *a priori*, to avoid the presence of undesirable and unrealistic zero-length branches (Bapst, 2014*a*, *b*). For our analyses, the minimum of 1 Myr was set.

Second, the *cal3* method, which is a stochastic calibration method that requires estimates of sampling and diversification (branching and extinction) rates to draw likely divergence dates under a birth–death-sampling model (Bapst, 2013; Lloyd *et al*., 2016). The fact that most crocodylomorph taxa are singletons (i.e., very few genera or species have multiple occurrences in different time intervals) prevented us from directly calculating speciation, extinction and sampling rates needed as inputs to the *cal3* method. Thus, when using this time-scaling method for our analyses, we adopted the same rates estimated for dinosaurs in Lloyd *et al*. (2016) (i.e., extinction and speciation rates = 0.935; sampling rate = 0.018), which used the apparent range-frequency distribution of dinosaurs in the Paleobiology Database for these estimates. Although essentially different from that of dinosaurs, the crocodylomorph fossil record is arguably comparable enough to result in similar rates (i.e., in both groups, many species are based on only single occurrences, having therefore no meaningful range data; Benson *et al*., 2018), and *a posteriori* comparison to other time-scaling methods demonstrated that results were qualitatively reasonable.

Finally, the *extended Hedman* method was proposed by Lloyd *et al*. (2016), and is expansion of the approach presented by Hedman (2010). It is a probabilistic that uses the ages of successive outgroup taxa relative to the age of the node of interest to date this node by sampling from uniform distributions (Lloyd *et al*. 2016, Brocklehurst, 2017).

Since the input phylogenies (i.e., the three alternatives topologies of the supertree, see above) were not completely resolved, we randomly resolved the polytomies, generating 20 completely resolved trees (the same number of trees was time-scaled with the FBD method) for each alternative phylogenetic scenario (i.e., with different positions of Thalattosuchia). These trees were then time-scaled using the three time-calibration methods. Time-scaling with the *mbl* and *cal3* methods were performed using the package *paleotree* (Bapst, 2012) in R version 3.5.1 (R Core Team, 2018), whilst the *Hedman* method was implemented also in R, using the protocol published by Lloyd *et al*. (2016).

## *Time bins used for time series correlations and disparity calculation*

| Lower limit (in Myr) | Upper limit (in Myr) |
| --- | --- |
| 7.246 | 0 |
| 15.97 | 7.246 |
| 23.03 | 15.97 |
| 33.9 | 23.03 |
| 40.4 | 33.9 |
| 48.6 | 40.4 |
| 55.8 | 48.6 |
| 61.7 | 55.8 |
| 66.043 | 61.7 |
| 70.6 | 66.043 |
| 84.9 | 70.6 |
| 94.3 | 84.9 |
| 99.7 | 94.3 |
| 112.6 | 99.7 |
| 125.45 | 112.6 |
| 136.4 | 125.45 |
| 145.5 | 136.4 |
| 155.7 | 145.5 |
| 164.7 | 155.7 |
| 171.6 | 164.7 |
| 183 | 171.6 |
| 189.6 | 183 |
| 201.6 | 189.6 |
| 205.6 | 201.6 |
| 221.5 | 205.6 |
| 235 | 221.5 |
| 242 | 235 |
| 252.3 | 242 |

# Supplementary results

## *FBD consensus trees*


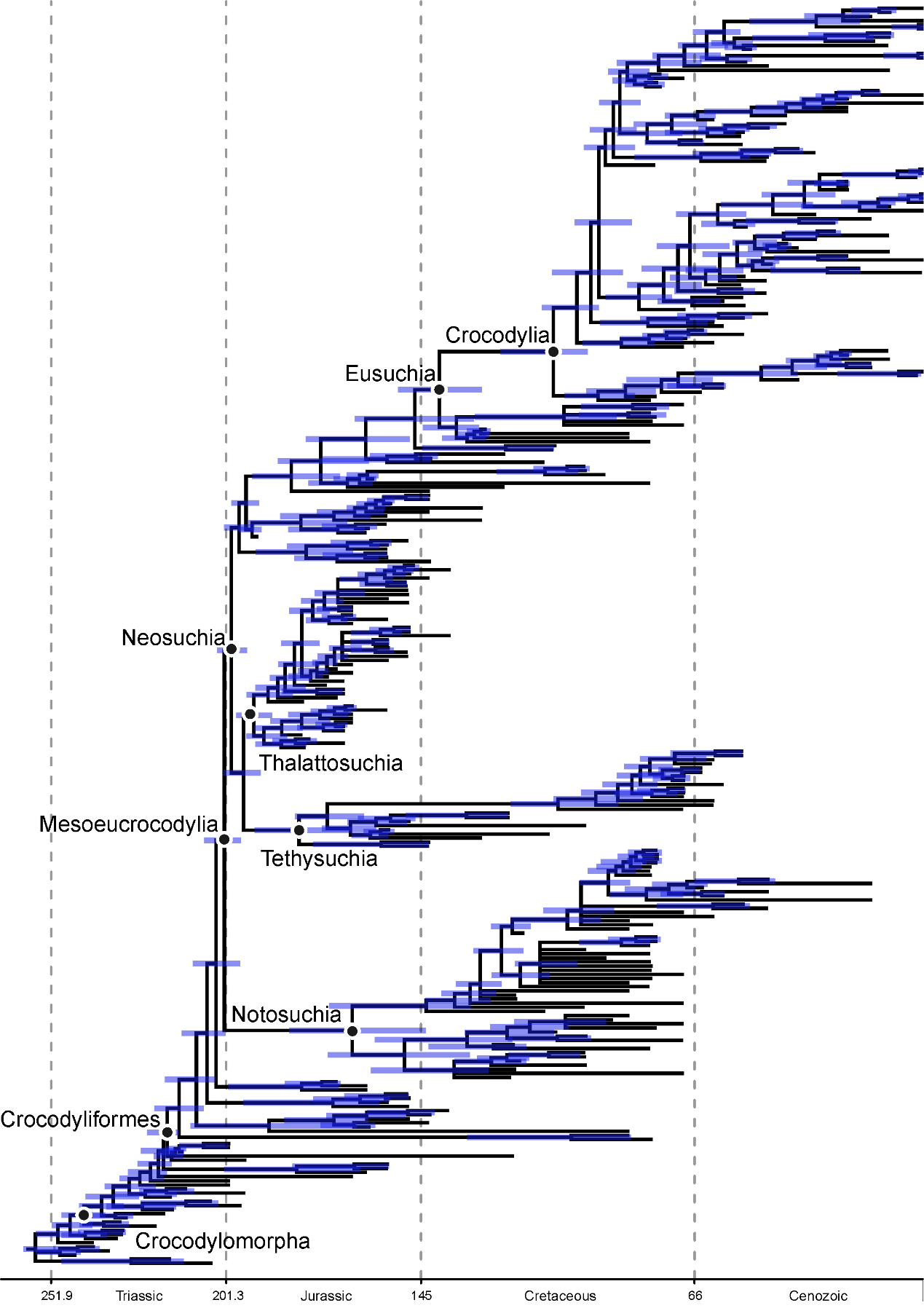


Figure S2. Consensus tree (50% majority rule tree) of Crocodylomorpha, with Thalattosuchia within Neosuchia. Node ages were inferred under a fossilized birth-death process, performing 10,000,000 generations of MCMC analyses. Blue bars indicate 95% Highest Posterior Density (HPD) time intervals.


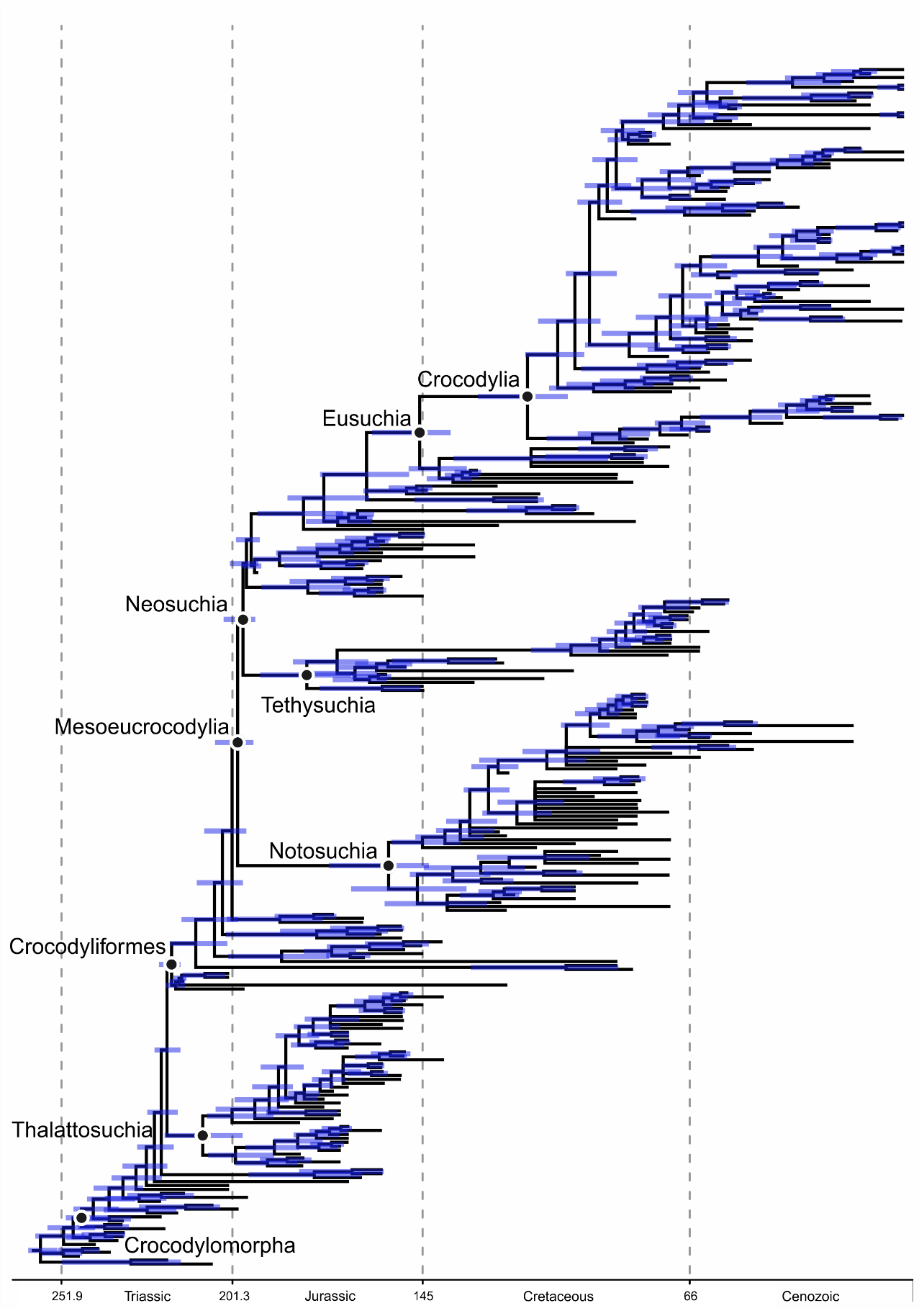


Figure S3. Consensus tree (50% majority rule tree) of Crocodylomorpha, with Thalattosuchia as the sister group of Crocodyliformes. Node ages were inferred under a fossilized birth-death process, performing 10,000,000 generations of MCMC analyses. Blue bars indicate 95% Highest Posterior Density (HPD) time intervals.


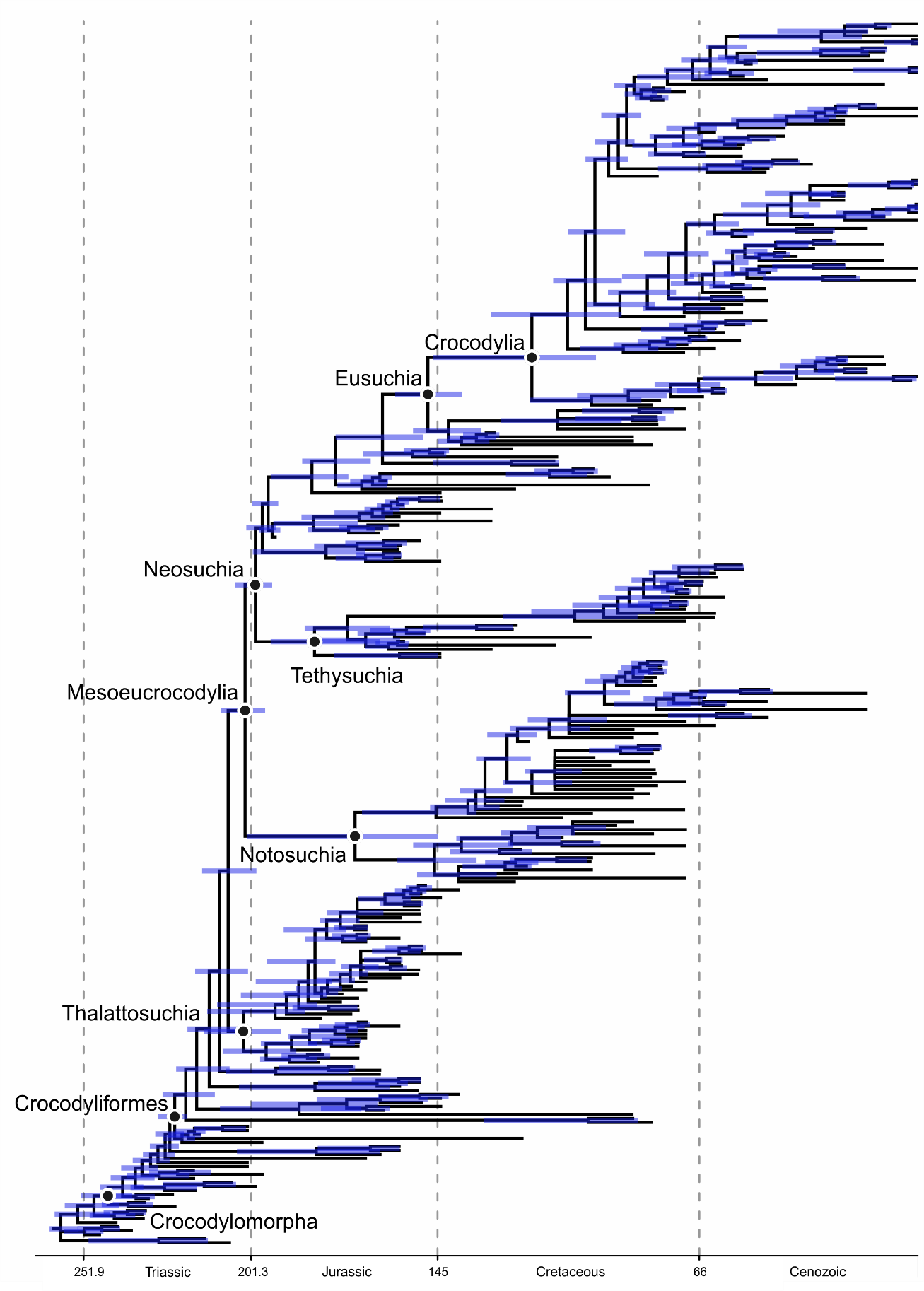


Figure S4. Consensus tree (50% majority rule tree) of Crocodylomorpha, with Thalattosuchia as the sister group of Mesoeucrocodylia. Node ages were inferred under a fossilized birth-death process, performing 10,000,000 generations of MCMC analyses. Blue bars indicate 95% Highest Posterior Density (HPD) time intervals.

## *Initial model comparison using APT time-scaling methods*


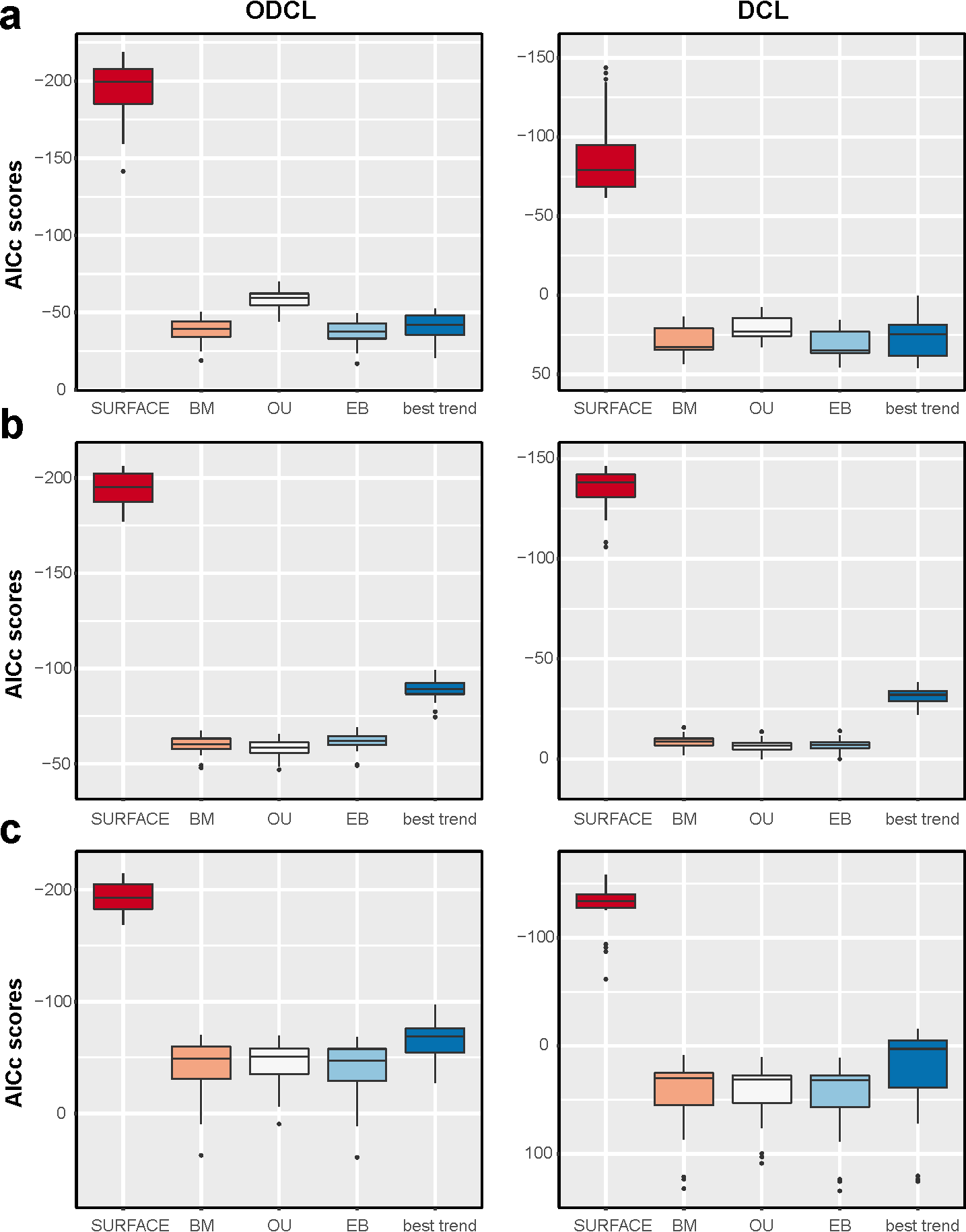


Figure S5. AICc scores of the evolutionary models fitted to crocodylomorph phylogeny and body size data. Results shown for two cranial measurements datasets (ODCL in the left column and DCL in the right one), as well as using three different APT time-scaling (i.e., *a posteriori*) methods to time-calibrate 20 randomly resolved phylogenies of Crocodylomorpha: (a) mbl, (b) Hedman, and (c) cal3 methods. For the trend-like models, only the AICc of the best model (“best trend”) is shown.

## *Correlations with abiotic factors*

Most of the regression analyses with body size and palaeotemperature data (Tables S2– S15) revealed very weak or non-significant correlations. In some cases, we did find significant correlations, but they were frequently inconsistent (i.e., correlations did not persist in both ODCL and DCL datasets or were absent when accounting for serial autocorrelation using GLS). The only conspicuous exception was found between mean body size values and palaeotemperatures from the Late Cretaceous (Maastrichtian) to the Recent (and, in particular, when using only taxa of the crown-group Crocodylia [Tables S8 and S9]).

A similar scenario was found for the correlation test between body size and paleolatitude (Tables S16– S29), with very weak or non-significant correlations. Our phylogenetic regressions of found some significant correlations, but in all cases the coefficient of determination (R^2^) was very low (always smaller than 0.1), indicating that the correlation is very weak and only a small proportion (less than 10%) of the body size variation observed can be explained by the palaeolatitudinal data.

### *Palaeotemperature*

Table S2. Results of regressions of body size proxy (maximum and mean log-transformed ODCL, using all species in the dataset) on the palaeotemperature proxies (δ^18^O data for tropical and temperate regions from Prokoph *et al*. [2008], and global δ^18^O data from Zachos *et al*. [2008]). Possible correlation was analysed using generalised least squares (GLS) regressions, incorporating a first-order autoregressive model, as well as ordinary least squares (OLS) regressions using untransformed data (assuming no serial correlation). *Significant at alpha = 0.05.

|  | **Prokoph (Early Triassic -recent): tropical palaeotemperatures** | | | | | | | | | | | | | | | |  |  |
| --- | --- | --- | --- | --- | --- | --- | --- | --- | --- | --- | --- | --- | --- | --- | --- | --- | --- | --- |
| **N** | **Maximum size** | | | | | | | | **Mean size** | | | | | | | | |  |
|  | **GLS** | | | | **OLS (untransformed)** | | | | **GLS** | | | | **OLS (untransformed)** | | | | |  |
| 26 | **Phi** | **Int.** | **Slope** | **AIC** | **R^2^** | **Int.** | **Slope** | **AIC** | **Phi** | **Int.** | **Slope** | **AIC** | **R^2^** | **Int.** | **Slope** | **AIC** | |  |
|  | 0.643 | 2.363 | 0.019 (0.75) | 2.565 | -0.004 | 2.438 | 0.063  (0.359) | 11.94 | 0.741 | 1.973 | -0.015 (0.685) | -20.353 | 0.004 | 2.073 | 0.049  (0.299) | -7.032 | |  |
|  | **Prokoph (Early Triassic -recent): temperate palaeotemperatures** | | | | | | | | | | | | | | | | | |
| **N** | **Maximum size** | | | | | | | | **Mean size** | | | | | | | | |  |
|  | **GLS** | | | | **OLS (untransformed)** | | | | **GLS** | | | | **OLS (untransformed)** | | | | |  |
| 23 | **Phi** | **Int.** | **Slope** | **AIC** | **R^2^** | **Int.** | **Slope** | **AIC** | **Phi** | **Int.** | **Slope** | **AIC** | **R^2^** | **Int.** | **Slope** | **AIC** | |  |
|  | 0.241 | 2.428 | -0.015 (0.729) | -15.324 | -0.038 | 2.426 | -0.017 (0.671) | -16.034 | 0.412 | 2.067 | 0.011 (0.755) | -22.54 | -0.027 | 2.079 | 0.022 (0.529) | -20.973 | |  |
|  | **Zachos (Late Cretaceous - recent): global palaeotemperatures** | | | | | | | | | | | | | | | | | |
| **N** | **Maximum size** | | | | | | | | **Mean size** | | | | | | | | |  |
|  | **GLS** | | | | **OLS (untransformed)** | | | | **GLS** | | | | **OLS (untransformed)** | | | | |  |
| 10 | **Phi** | **Int.** | **Slope** | **AIC** | **R^2^** | **Int.** | **Slope** | **AIC** | **Phi** | **Int.** | **Slope** | **AIC** | **R^2^** | **Int.** | **Slope** | **AIC** | |  |
|  | 0.347 | 2.34 | 0.045 (0.397) | -9.539 | -0.016 | 2.346 | 0.039 (0.383) | -10.306 | -0.046 | 2.022 | 0.055* (0.002) | -31.576 | 0.635 | 2.023 | 0.054*  (0.003) | -33.557 | |  |

Table S3. Results of regressions of body size proxy (maximum and mean log-transformed DCL, using all species in the dataset) on the palaeotemperature proxies (δ^18^O data for tropical and temperate regions from Prokoph *et al*. [2008], and global δ^18^O data from Zachos *et al*. [2008]). Possible correlation was analysed using generalised least squares (GLS) regressions, incorporating a first-order autoregressive model, as well as ordinary least squares (OLS) regressions using untransformed data (assuming no serial correlation). *Significant at alpha = 0.05.

|  | **Prokoph (Early Triassic -recent): tropical palaeotemperatures** | | | | | | | | | | | | | | | |  |  |
| --- | --- | --- | --- | --- | --- | --- | --- | --- | --- | --- | --- | --- | --- | --- | --- | --- | --- | --- |
| **N** | **Maximum size** | | | | | | | | **Mean size** | | | | | | | | |  |
|  | **GLS** | | | | **OLS (untransformed)** | | | | **GLS** | | | | **OLS (untransformed)** | | | | |  |
| 26 | **Phi** | **Int.** | **Slope** | **AIC** | **R^2^** | **Int.** | **Slope** | **AIC** | **Phi** | **Int.** | **Slope** | **AIC** | **R^2^** | **Int.** | **Slope** | **AIC** | |  |
|  | 0.634 | 2.909 | 0.048 (0.508) | 11.415 | 0.031 | 3.01 | 0.106 (0.19) | 20.16 | 0.723 | 2.367 | -0.029 (0.565) | -6.284 | -0.027 | 2.47 | 0.035 (0.564) | 7.111 | |  |
|  | **Prokoph (Early Triassic -recent): temperate palaeotemperatures** | | | | | | | | | | | | | | | | | |
| **N** | **Maximum size** | | | | | | | | **Mean size** | | | | | | | | |  |
|  | **GLS** | | | | **OLS (untransformed)** | | | | **GLS** | | | | **OLS (untransformed)** | | | | |  |
| 23 | **Phi** | **Int.** | **Slope** | **AIC** | **R^2^** | **Int.** | **Slope** | **AIC** | **Phi** | **Int.** | **Slope** | **AIC** | **R^2^** | **Int.** | **Slope** | **AIC** | |  |
|  | 0.108 | 2.956 | -0.025 (0.57) | -11.036 | -0.033 | 2.958 | -0.022 (0.602) | -12.782 | 0.505 | 2.468 | -0.007 (0.888) | -6.265 | -0.041 | 2.496 | 0.019 (0.725) | -2.48 | |  |
|  | **Zachos (Late Cretaceous - recent): global palaeotemperatures** | | | | | | | | | | | | | | | | | |
| **N** | **Maximum size** | | | | | | | | **Mean size** | | | | | | | | |  |
|  | **GLS** | | | | **OLS (untransformed)** | | | | **GLS** | | | | **OLS (untransformed)** | | | | |  |
| 10 | **Phi** | **Int.** | **Slope** | **AIC** | **R^2^** | **Int.** | **Slope** | **AIC** | **Phi** | **Int.** | **Slope** | **AIC** | **R^2^** | **Int.** | **Slope** | **AIC** | |  |
|  | 0.265 | 2.9 | 0.049 (0.126) | -19.517 | 0.27 | 2.898 | 0.052 (0.07) | -20.96 | 0.014 | 2.433 | 0.081* (0.011) | -19.577 | 0.527 | 2.433 | 0.081* (0.01) | -21.575 | |  |

Table S4. Results of regressions of body size proxy (maximum and mean log-transformed ODCL, using only marine species in the dataset) on the palaeotemperature proxies (δ^18^O data for tropical and temperate regions from Prokoph *et al*. [2008], and global δ^18^O data from Zachos *et al*. [2008]). Possible correlation was analysed using generalised least squares (GLS) regressions, incorporating a first-order autoregressive model, as well as ordinary least squares (OLS) regressions using untransformed data (assuming no serial correlation). *Significant at alpha = 0.05.

|  | **Prokoph (Early Triassic -recent): tropical palaeotemperatures** | | | | | | | | | | | | | | | |  |  |
| --- | --- | --- | --- | --- | --- | --- | --- | --- | --- | --- | --- | --- | --- | --- | --- | --- | --- | --- |
| **N** | **Maximum size** | | | | | | | | **Mean size** | | | | | | | | |  |
|  | **GLS** | | | | **OLS (untransformed)** | | | | **GLS** | | | | **OLS (untransformed)** | | | | |  |
| 18 | **Phi** | **Int.** | **Slope** | **AIC** | **R^2^** | **Int.** | **Slope** | **AIC** | **Phi** | **Int.** | **Slope** | **AIC** | **R^2^** | **Int.** | **Slope** | **AIC** | |  |
|  | 0.56 | 2.358 | -0.025 (0.536) | -25.542 | 0.201 | 2.276 | -0.11* (0.035) | -24.167 | 0.014 | 2.239 | -0.017 (0.451) | -49.171 | -0.023 | 2.239 | -0.017 (0.448) | -51.167 | |  |
|  | **Prokoph (Early Triassic -recent): temperate palaeotemperatures** | | | | | | | | | | | | | | | | | |
| **N** | **Maximum size** | | | | | | | | **Mean size** | | | | | | | | |  |
|  | **GLS** | | | | **OLS (untransformed)** | | | | **GLS** | | | | **OLS (untransformed)** | | | | |  |
| 17 | **Phi** | **Int.** | **Slope** | **AIC** | **R^2^** | **Int.** | **Slope** | **AIC** | **Phi** | **Int.** | **Slope** | **AIC** | **R^2^** | **Int.** | **Slope** | **AIC** | |  |
|  | 0.708 | 2.423 | 0.079 (0.059) | -23.953 | -0.066 | 2.398 | -0.002 (0.955) | -16.916 | 0.758 | 2.273 | 0.022 (0.058) | -66.294 | -0.027 | 2.273 | 0.011 (0.463) | -56.901 | |  |
|  | **Zachos (Late Cretaceous - recent): global palaeotemperatures** | | | | | | | | | | | | | | | | | |
| **N** | **Maximum size** | | | | | | | | **Mean size** | | | | | | | | |  |
|  | **GLS** | | | | **OLS (untransformed)** | | | | **GLS** | | | | **OLS (untransformed)** | | | | |  |
| 10 | **Phi** | **Int.** | **Slope** | **AIC** | **R^2^** | **Int.** | **Slope** | **AIC** | **Phi** | **Int.** | **Slope** | **AIC** | **R^2^** | **Int.** | **Slope** | **AIC** | |  |
|  | -0.143 | 2.422 | -0.045 (0.054) | -21.432 | 0.22 | 2.417 | -0.042 (0.096) | -23.261 | 0.627 | 2.252 | -0.006 (0.654) | -39.327 | -0.088 | 2.241 | 0.005 (0.617) | -38.084 | |  |

Table S5. Results of regressions of body size proxy (maximum and mean log-transformed DCL, using only marine species in the dataset) on the palaeotemperature proxies (δ^18^O data for tropical and temperate regions from Prokoph *et al*. [2008], and global δ^18^O data from Zachos *et al*. [2008]). Possible correlation was analysed using generalised least squares (GLS) regressions, incorporating a first-order autoregressive model, as well as ordinary least squares (OLS) regressions using untransformed data (assuming no serial correlation). *Significant at alpha = 0.05.

|  | **Prokoph (Early Triassic -recent): tropical palaeotemperatures** | | | | | | | | | | | | | | | |  |  |  |
| --- | --- | --- | --- | --- | --- | --- | --- | --- | --- | --- | --- | --- | --- | --- | --- | --- | --- | --- | --- |
| **N** | **Maximum size** | | | | | | | | **Mean size** | | | | | | | | | |  |
|  | **GLS** | | | | **OLS (untransformed)** | | | | **GLS** | | | | **OLS (untransformed)** | | | | | |  |
| 18 | **Phi** | **Int.** | **Slope** | **AIC** | **R^2^** | **Int.** | **Slope** | **AIC** | **Phi** | **Int.** | **Slope** | **AIC** | **R^2^** | **Int.** | **Slope** | **AIC** | |  |  |
|  | 0.601 | 2.936 | -0.01 (0.714) | -35.977 | -0.052 | 2.932 | -0.015 (0.705) | -31.507 | 0.752 | 2.82 | -0.015 (0.545) | -39.362 | -0.004 | 2.862 | 0.04 (0.35) | -29.321 | |  |  |
|  | **Prokoph (Early Triassic -recent): temperate palaeotemperatures** | | | | | | | | | | | | | | | | | | |
| **N** | **Maximum size** | | | | | | | | **Mean size** | | | | | | | | | |  |
|  | **GLS** | | | | **OLS (untransformed)** | | | | **GLS** | | | | **OLS (untransformed)** | | | | | |  |
| 18 | **Phi** | **Int.** | **Slope** | **AIC** | **R^2^** | **Int.** | **Slope** | **AIC** | **Phi** | **Int.** | **Slope** | **AIC** | **R^2^** | **Int.** | **Slope** | **AIC** | |  |  |
|  | 0.449 | 2.984 | 0.055* (0.028) | -40.881 | 0.335 | 2.996 | 0.071* (0.006) | -39.789 | 0.657 | 2.865 | 0.052* (0.016) | -45.475 | 0.471 | 2.878 | 0.09* (0.0009) | -40.862 | |  |  |
|  | **Zachos (Late Cretaceous - recent): global palaeotemperatures** | | | | | | | | | | | | | | | | | | |
| **N** | **Maximum size** | | | | | | | | **Mean size** | | | | | | | | | |  |
|  | **GLS** | | | | **OLS (untransformed)** | | | | **GLS** | | | | **OLS (untransformed)** | | | | | |  |
| 10 | **Phi** | **Int.** | **Slope** | **AIC** | **R^2^** | **Int.** | **Slope** | **AIC** | **Phi** | **Int.** | **Slope** | **AIC** | **R^2^** | **Int.** | **Slope** | **AIC** | |  |  |
|  | 0.208 | 2.906 | 0.042 (0.148) | -20.859 | 0.215 | 2.906 | 0.043 (0.099) | -22.527 | 0.824 | 2.781 | 0.036 (0.256) | -24.953 | 0.692 | 2.715 | 0.092* (0.001) | -25.525 | |  |  |

Table S6. Results of regressions of body size proxy (maximum and mean log-transformed ODCL, using only non-marine species in the dataset) on the palaeotemperature proxies (δ^18^O data for tropical and temperate regions from Prokoph *et al*. [2008], and global δ^18^O data from Zachos *et al*. [2008]). Possible correlation was analysed using generalised least squares (GLS) regressions, incorporating a first-order autoregressive model, as well as ordinary least squares (OLS) regressions using untransformed data (assuming no serial correlation). *Significant at alpha = 0.05.

|  | **Prokoph (Early Triassic -recent): tropical palaeotemperatures** | | | | | | | | | | | | | | | |  |  |
| --- | --- | --- | --- | --- | --- | --- | --- | --- | --- | --- | --- | --- | --- | --- | --- | --- | --- | --- |
| **N** | **Maximum size** | | | | | | | | **Mean size** | | | | | | | | |  |
|  | **GLS** | | | | **OLS (untransformed)** | | | | **GLS** | | | | **OLS (untransformed)** | | | | |  |
| 26 | **Phi** | **Int.** | **Slope** | **AIC** | **R^2^** | **Int.** | **Slope** | **AIC** | **Phi** | **Int.** | **Slope** | **AIC** | **R^2^** | **Int.** | **Slope** | **AIC** | |  |
|  | 0.553 | 2.32 | 0.043 (0.504) | 4.843 | 0.011 | 2.366 | 0.075 (0.264) | 11.094 | 0.64 | 1.978 | 0.029 (0.453) | -21.012 | 0.049 | 2.023 | 0.065 (0.142) | -11.564 | |  |
|  | **Prokoph (Early Triassic -recent): temperate palaeotemperatures** | | | | | | | | | | | | | | | | | |
| **N** | **Maximum size** | | | | | | | | **Mean size** | | | | | | | | |  |
|  | **GLS** | | | | **OLS (untransformed)** | | | | **GLS** | | | | **OLS (untransformed)** | | | | |  |
| 23 | **Phi** | **Int.** | **Slope** | **AIC** | **R^2^** | **Int.** | **Slope** | **AIC** | **Phi** | **Int.** | **Slope** | **AIC** | **R^2^** | **Int.** | **Slope** | **AIC** | |  |
|  | 0.354 | 2.291 | -0.065 (0.232) | -6.825 | 0.022 | 2.291 | -0.06 (0.232) | -6.129 | 0.523 | 1.947 | -0.042 (0.299) | -21.071 | -0.037 | 1.967 | -0.017 (0.65) | -17.942 | |  |
|  | **Zachos (Late Cretaceous - recent): global palaeotemperatures** | | | | | | | | | | | | | | | | | |
| **N** | **Maximum size** | | | | | | | | **Mean size** | | | | | | | | |  |
|  | **GLS** | | | | **OLS (untransformed)** | | | | **GLS** | | | | **OLS (untransformed)** | | | | |  |
| 10 | **Phi** | **Int.** | **Slope** | **AIC** | **R^2^** | **Int.** | **Slope** | **AIC** | **Phi** | **Int.** | **Slope** | **AIC** | **R^2^** | **Int.** | **Slope** | **AIC** | |  |
|  | 0.209 | 2.228 | 0.068 (0.366) | -0.829 | -0.011 | 2.236 | 0.06 (0.371) | -2.397 | -0.157 | 1.964 | 0.06* (0.007) | -24.96 | 0.502 | 1.965 | 0.06* (0.013) | -26.706 | |  |

Table S7. Results of regressions of body size proxy (maximum and mean log-transformed DCL, using only non-marine species in the dataset) on the palaeotemperature proxies (δ^18^O data for tropical and temperate regions from Prokoph *et al*. [2008], and global δ^18^O data from Zachos *et al*. [2008]). Possible correlation was analysed using generalised least squares (GLS) regressions, incorporating a first-order autoregressive model, as well as ordinary least squares (OLS) regressions using untransformed data (assuming no serial correlation). *Significant at alpha = 0.05.

|  | **Prokoph (Early Triassic -recent): tropical palaeotemperatures** | | | | | | | | | | | | | | | |  |  |
| --- | --- | --- | --- | --- | --- | --- | --- | --- | --- | --- | --- | --- | --- | --- | --- | --- | --- | --- |
| **N** | **Maximum size** | | | | | | | | **Mean size** | | | | | | | | |  |
|  | **GLS** | | | | **OLS (untransformed)** | | | | **GLS** | | | | **OLS (untransformed)** | | | | |  |
| 26 | **Phi** | **Int.** | **Slope** | **AIC** | **R^2^** | **Int.** | **Slope** | **AIC** | **Phi** | **Int.** | **Slope** | **AIC** | **R^2^** | **Int.** | **Slope** | **AIC** | |  |
|  | 0.563 | 2.753 | 0.024 (0.763) | 15.858 | -0.011 | 2.82 | 0.069 (0.406) | 22.285 | 0.624 | 2.339 | 0.017 (0.725) | -8.74 | -0.018 | 2.366 | 0.04 (0.466) | 0.623 | |  |
|  | **Prokoph (Early Triassic -recent): temperate palaeotemperatures** | | | | | | | | | | | | | | | | | |
| **N** | **Maximum size** | | | | | | | | **Mean size** | | | | | | | | |  |
|  | **GLS** | | | | **OLS (untransformed)** | | | | **GLS** | | | | **OLS (untransformed)** | | | | |  |
| 23 | **Phi** | **Int.** | **Slope** | **AIC** | **R^2^** | **Int.** | **Slope** | **AIC** | **Phi** | **Int.** | **Slope** | **AIC** | **R^2^** | **Int.** | **Slope** | **AIC** | |  |
|  | 0.317 | 2.76 | -0.071 (0.312) | 5.801 | 0.003 | 2.762 | -0.066 (0.309) | 5.997 | 0.518 | 2.32 | -0.046 (0.387) | -7.779 | -0.033 | 2.335 | -0.027 (0.6) | -4.075 | |  |
|  | **Zachos (Late Cretaceous - recent): global palaeotemperatures** | | | | | | | | | | | | | | | | | |
| **N** | **Maximum size** | | | | | | | | **Mean size** | | | | | | | | |  |
|  | **GLS** | | | | **OLS (untransformed)** | | | | **GLS** | | | | **OLS (untransformed)** | | | | |  |
| 10 | **Phi** | **Int.** | **Slope** | **AIC** | **R^2^** | **Int.** | **Slope** | **AIC** | **Phi** | **Int.** | **Slope** | **AIC** | **R^2^** | **Int.** | **Slope** | **AIC** | |  |
|  | -0.083 | 2.633 | 0.095 (0.172) | 0.504 | 0.104 | 2.633 | 0.096 (0.189) | -1.426 | -0.089 | 2.345 | 0.07* (0.027) | -16.045 | 0.376 | 2.346 | 0.07* (0.034) | -18.272 | |  |

Table S8. Results of regressions of body size proxy (maximum and mean log-transformed ODCL, using only crocodylian species in the dataset) on the palaeotemperature proxies (global δ^18^O data from Zachos *et al*. [2008], from the Late Cretaceous to Recent). Possible correlation was analysed using generalised least squares (GLS) regressions, incorporating a first-order autoregressive model, as well as ordinary least squares (OLS) regressions using untransformed data (assuming no serial correlation). *Significant at alpha = 0.05.

|  | **Zachos (Late Cretaceous - recent): global palaeotemperatures** | | | | | | | | | | | | | | | | |
| --- | --- | --- | --- | --- | --- | --- | --- | --- | --- | --- | --- | --- | --- | --- | --- | --- | --- |
| **N** | **Maximum size** | | | | | | | | **Mean size** | | | | | | | |  |
|  | **GLS** | | | | **OLS (untransformed)** | | | | **GLS** | | | | **OLS (untransformed)** | | | |  |
| 10 | **Phi** | **Int.** | **Slope** | **AIC** | **R^2^** | **Int.** | **Slope** | **AIC** | **Phi** | **Int.** | **Slope** | **AIC** | **R^2^** | **Int.** | **Slope** | **AIC** |  |
|  | 0.19 | 2.133 | 0.121* (0.017) | -11.989 | 0.554 | 2.124 | 0.127* (0.008) | -13.662 | -0.297 | 1.98 | 0.075* (0.0003) | -29.953 | 0.698 | 1.987 | 0.07* (0.001) | -31.137 |  |

Table S9. Results of regressions of body size proxy (maximum and mean log-transformed DCL, using only crocodylian species in the dataset) on the palaeotemperature proxies (global δ^18^O data from Zachos *et al*. [2008], from the Late Cretaceous to Recent). Possible correlation was analysed using generalised least squares (GLS) regressions, incorporating a first-order autoregressive model, as well as ordinary least squares (OLS) regressions using untransformed data (assuming no serial correlation). *Significant at alpha = 0.05.

|  | **Zachos (Late Cretaceous - recent): global palaeotemperatures** | | | | | | | | | | | | | | | | |
| --- | --- | --- | --- | --- | --- | --- | --- | --- | --- | --- | --- | --- | --- | --- | --- | --- | --- |
| **N** | **Maximum size** | | | | | | | | **Mean size** | | | | | | | |  |
|  | **GLS** | | | | **OLS (untransformed)** | | | | **GLS** | | | | **OLS (untransformed)** | | | |  |
| 10 | **Phi** | **Int.** | **Slope** | **AIC** | **R^2^** | **Int.** | **Slope** | **AIC** | **Phi** | **Int.** | **Slope** | **AIC** | **R^2^** | **Int.** | **Slope** | **AIC** |  |
|  | -0.215 | 2.618 | 0.165* (0.001) | -10.724 | 0.632 | 2.627 | 0.157* (0.003) | -12.355 | -0.235 | 2.386 | 0.105* (0.0007) | -20.748 | 0.647 | 2.395 | 0.098* (0.003) | -22.325 |  |

Table S10. Results of regressions of body size proxy (maximum and mean log-transformed ODCL, using only notosuchian species in the dataset) on the palaeotemperature proxies (tropical δ^18^O data from Prokoph *et al*. [2008], from the Aptian to the Eocene). Possible correlation was analysed using generalised least squares (GLS) regressions, incorporating a first-order autoregressive model, as well as ordinary least squares (OLS) regressions using untransformed data (assuming no serial correlation). *Significant at alpha = 0.05.

|  | **Prokoph (Aptian - Eocene): tropical palaeotemperatures** | | | | | | | | | | | | | | | | |
| --- | --- | --- | --- | --- | --- | --- | --- | --- | --- | --- | --- | --- | --- | --- | --- | --- | --- |
| **N** | **Maximum size** | | | | | | | | **Mean size** | | | | | | | |  |
|  | **GLS** | | | | **OLS (untransformed)** | | | | **GLS** | | | | **OLS (untransformed)** | | | |  |
| 10 | **Phi** | **Int.** | **Slope** | **AIC** | **R^2^** | **Int.** | **Slope** | **AIC** | **Phi** | **Int.** | **Slope** | **AIC** | **R^2^** | **Int.** | **Slope** | **AIC** |  |
|  | 0.272 | 2.114 | -0.013 (0.812) | -5.557 | -0.115 | 2.118 | -0.014 (0.798) | -6.786 | 0.702 | 1.925 | -0.029 (0.472) | -11.724 | -0.122 | 1.957 | -0.005 (0.904) | -10.071 |  |

Table S11. Results of regressions of body size proxy (maximum and mean log-transformed DCL, using only notosuchian species in the dataset) on the palaeotemperature proxies (tropical δ^18^O data from Prokoph *et al*. [2008], from the Aptian to the Eocene). Possible correlation was analysed using generalised least squares (GLS) regressions, incorporating a first-order autoregressive model, as well as ordinary least squares (OLS) regressions using untransformed data (assuming no serial correlation). *Significant at alpha = 0.05.

|  | **Prokoph (Aptian - Eocene): tropical palaeotemperatures** | | | | | | | | | | | | | | | | |
| --- | --- | --- | --- | --- | --- | --- | --- | --- | --- | --- | --- | --- | --- | --- | --- | --- | --- |
| **N** | **Maximum size** | | | | | | | | **Mean size** | | | | | | | |  |
|  | **GLS** | | | | **OLS (untransformed)** | | | | **GLS** | | | | **OLS (untransformed)** | | | |  |
| 10 | **Phi** | **Int.** | **Slope** | **AIC** | **R^2^** | **Int.** | **Slope** | **AIC** | **Phi** | **Int.** | **Slope** | **AIC** | **R^2^** | **Int.** | **Slope** | **AIC** |  |
|  | 0.06 | 2.622 | -0.014 (0.699) | -12.63 | -0.092 | 2.618 | -0.017 (0.64) | -14.601 | 0.758 | 2.313 | -0.055 (0.3) | -6.073 | -0.123 | 2.355 | -0.005 (0.928) | -3.54 |  |

Table S12. Results of regressions of body size proxy (maximum and mean log-transformed ODCL, using only thalattosuchian species in the dataset) on the palaeotemperature proxies (tropical δ^18^O data from Prokoph *et al*. [2008], for the Jurassic). Possible correlation was analysed using generalised least squares (GLS) regressions, incorporating a first-order autoregressive model, as well as ordinary least squares (OLS) regressions using untransformed data (assuming no serial correlation). *Significant at alpha = 0.05.

|  | **Prokoph (Jurassic): tropical palaeotemperatures** | | | | | | | | | | | | | | | |  |  |
| --- | --- | --- | --- | --- | --- | --- | --- | --- | --- | --- | --- | --- | --- | --- | --- | --- | --- | --- |
| **N** | **Maximum size** | | | | | | | | **Mean size** | | | | | | | | |  |
|  | **GLS** | | | | **OLS (untransformed)** | | | | **GLS** | | | | **OLS (untransformed)** | | | | |  |
| 7 | **Phi** | **Int.** | **Slope** | **AIC** | **R^2^** | **Int.** | **Slope** | **AIC** | **Phi** | **Int.** | **Slope** | **AIC** | **R^2^** | **Int.** | **Slope** | **AIC** | |  |
|  | 0.809 | 2.396 | -0.051 (0.308) | -5.062 | 0.059 | 2.322 | -0.11 (0.292) | -2.309 | -0.184 | 2.224 | -0.038 (0.455) | -10.311 | -0.09 | 2.232 | -0.033 (0.509) | -12.067 | |  |
|  | **Prokoph (Jurassic): temperate palaeotemperatures** | | | | | | | | | | | | | | | | | |
| **N** | **Maximum size** | | | | | | | | **Mean size** | | | | | | | | |  |
|  | **GLS** | | | | **OLS (untransformed)** | | | | **GLS** | | | | **OLS (untransformed)** | | | | |  |
| 7 | **Phi** | **Int.** | **Slope** | **AIC** | **R^2^** | **Int.** | **Slope** | **AIC** | **Phi** | **Int.** | **Slope** | **AIC** | **R^2^** | **Int.** | **Slope** | **AIC** | |  |
|  | 0.808 | 2.526 | 0.074 (0.098) | -7.658 | 0.452 | 2.633 | 0.152 (0.058) | -6.096 | -0.369 | 2.366 | 0.082* (0.003) | -22.184 | 0.778 | 2.369 | 0.086* (0.005) | -23.214 | |  |

Table S13. Results of regressions of body size proxy (maximum and mean log-transformed DCL, using only thalattosuchian species in the dataset) on the palaeotemperature proxies (tropical δ^18^O data from Prokoph *et al*. [2008], for the Jurassic). Possible correlation was analysed using generalised least squares (GLS) regressions, incorporating a first-order autoregressive model, as well as ordinary least squares (OLS) regressions using untransformed data (assuming no serial correlation). *Significant at alpha = 0.05.

|  | **Prokoph (Jurassic): tropical palaeotemperatures** | | | | | | | | | | | | | | | |  |  |
| --- | --- | --- | --- | --- | --- | --- | --- | --- | --- | --- | --- | --- | --- | --- | --- | --- | --- | --- |
| **N** | **Maximum size** | | | | | | | | **Mean size** | | | | | | | | |  |
|  | **GLS** | | | | **OLS (untransformed)** | | | | **GLS** | | | | **OLS (untransformed)** | | | | |  |
| 7 | **Phi** | **Int.** | **Slope** | **AIC** | **R^2^** | **Int.** | **Slope** | **AIC** | **Phi** | **Int.** | **Slope** | **AIC** | **R^2^** | **Int.** | **Slope** | **AIC** | |  |
|  | 0.661 | 2.856 | -0.054 (0.176) | -10.26 | 0.192 | 2.814 | -0.088 (0.179) | -9.432 | -0.124 | 2.727 | -0.042 (0.391) | -10.851 | -0.022 | 2.728 | -0.041 (0.394) | -12.753 | |  |
|  | **Prokoph (Jurassic): temperate palaeotemperatures** | | | | | | | | | | | | | | | | | |
| **N** | **Maximum size** | | | | | | | | **Mean size** | | | | | | | | |  |
|  | **GLS** | | | | **OLS (untransformed)** | | | | **GLS** | | | | **OLS (untransformed)** | | | | |  |
| 7 | **Phi** | **Int.** | **Slope** | **AIC** | **R^2^** | **Int.** | **Slope** | **AIC** | **Phi** | **Int.** | **Slope** | **AIC** | **R^2^** | **Int.** | **Slope** | **AIC** | |  |
|  | 0.553 | 2.995 | 0.07 (0.069) | -12.556 | 0.563 | 3.046 | 0.107* (0.031) | -13.734 | 0.582 | 2.852 | 0.072 (0.056) | -12.788 | 0.162 | 2.839 | 0.051 (0.201) | -14.155 | |  |

Table S14. Results of regressions of body size proxy (maximum and mean log-transformed ODCL, using only tethysuchian species in the dataset) on the palaeotemperature proxies (tropical δ^18^O data from Prokoph *et al*. [2008], from the Late Jurassic to the Eocene). Possible correlation was analysed using generalised least squares (GLS) regressions, incorporating a first-order autoregressive model, as well as ordinary least squares (OLS) regressions using untransformed data (assuming no serial correlation). *Significant at alpha = 0.05.

|  | **Prokoph (Late Jurassic – Eocene): tropical palaeotemperatures** | | | | | | | | | | | | | | | |  |  |
| --- | --- | --- | --- | --- | --- | --- | --- | --- | --- | --- | --- | --- | --- | --- | --- | --- | --- | --- |
| **N** | **Maximum size** | | | | | | | | **Mean size** | | | | | | | | |  |
|  | **GLS** | | | | **OLS (untransformed)** | | | | **GLS** | | | | **OLS (untransformed)** | | | | |  |
| 13 | **Phi** | **Int.** | **Slope** | **AIC** | **R^2^** | **Int.** | **Slope** | **AIC** | **Phi** | **Int.** | **Slope** | **AIC** | **R^2^** | **Int.** | **Slope** | **AIC** | |  |
|  | -0.554 | 2.243 | -0.145* (0.004) | -5.113 | 0.138 | 2.288 | -0.108 (0.115) | -2.267 | -0.448 | 2.096 | -0.154* (0.0002) | -15.18 | 0.493 | 2.116 | -0.142* (0.004) | -14.409 | |  |
|  | **Prokoph (Late Jurassic – Eocene): temperate palaeotemperatures** | | | | | | | | | | | | | | | | | |
| **N** | **Maximum size** | | | | | | | | **Mean size** | | | | | | | | |  |
|  | **GLS** | | | | **OLS (untransformed)** | | | | **GLS** | | | | **OLS (untransformed)** | | | | |  |
| 13 | **Phi** | **Int.** | **Slope** | **AIC** | **R^2^** | **Int.** | **Slope** | **AIC** | **Phi** | **Int.** | **Slope** | **AIC** | **R^2^** | **Int.** | **Slope** | **AIC** | |  |
|  | -0.223 | 2.276 | -0.142 (0.051) | -1.969 | 0.202 | 2.27 | -0.15 (0.069) | -3.281 | 0.113 | 2.165 | -0.129 (0.063) | -7.028 | 0.226 | 2.163 | -0.127 (0.057) | -8.891 | |  |

Table S15. Results of regressions of body size proxy (maximum and mean log-transformed DCL, using only tethysuchian species in the dataset) on the palaeotemperature proxies (tropical δ^18^O data from Prokoph *et al*. [2008], from the Late Jurassic to the Eocene). Possible correlation was analysed using generalised least squares (GLS) regressions, incorporating a first-order autoregressive model, as well as ordinary least squares (OLS) regressions using untransformed data (assuming no serial correlation). *Significant at alpha = 0.05.

|  | **Prokoph (Late Jurassic – Eocene): tropical palaeotemperatures** | | | | | | | | | | | | | | | |  |  |
| --- | --- | --- | --- | --- | --- | --- | --- | --- | --- | --- | --- | --- | --- | --- | --- | --- | --- | --- |
| **N** | **Maximum size** | | | | | | | | **Mean size** | | | | | | | | |  |
|  | **GLS** | | | | **OLS (untransformed)** | | | | **GLS** | | | | **OLS (untransformed)** | | | | |  |
| 12 | **Phi** | **Int.** | **Slope** | **AIC** | **R^2^** | **Int.** | **Slope** | **AIC** | **Phi** | **Int.** | **Slope** | **AIC** | **R^2^** | **Int.** | **Slope** | **AIC** | |  |
|  | 0.53 | 3.02 | -0.004 (0.914) | -11.772 | -0.061 | 2.993 | -0.03 (0.559) | -10.039 | 0.483 | 2.848 | -0.045 (0.327) | -10.28 | 0.075 | 2.814 | -0.07 (0.198) | -9.441 | |  |
|  | **Prokoph (Late Jurassic – Eocene): temperate palaeotemperatures** | | | | | | | | | | | | | | | | | |
| **N** | **Maximum size** | | | | | | | | **Mean size** | | | | | | | | |  |
|  | **GLS** | | | | **OLS (untransformed)** | | | | **GLS** | | | | **OLS (untransformed)** | | | | |  |
| 12 | **Phi** | **Int.** | **Slope** | **AIC** | **R^2^** | **Int.** | **Slope** | **AIC** | **Phi** | **Int.** | **Slope** | **AIC** | **R^2^** | **Int.** | **Slope** | **AIC** | |  |
|  | 0.528 | 2.941 | -0.08 (0.15) | -14.367 | 0.105 | 2.941 | -0.083 (0.16) | -12.088 | 0.503 | 2.799 | -0.105 (0.081) | -12.934 | 0.221 | 2.782 | -0.114 (0.069) | -11.507 | |  |

### *Palaeolatitude*

Table S16. Results of regressions of log-transformed body length proxy (using all species in the ODCL cranial measurement dataset) on the palaeolatitudinal data. Possible correlation was analysed using ordinary least squares (OLS) and phylogenetic generalised least squares (PGLS) regressions. *Significant at alpha = 0.05.

| **N** | **OLS** | | | | **PGLS** | | | |
| --- | --- | --- | --- | --- | --- | --- | --- | --- |
| 195 | **R^2^** | **Intercept** | **Slope** | **AIC** | **R^2^** | **Intercept** | **Slope** | **AIC** |
|  | 0.013 | 2.13 | -0.002 (0.059) | 43.284 | 0.003 | 1.77 | -0.001 (0.194) | -39.972 |

Table S17. Results of regressions of log-transformed body length proxy (using all species in the DCL cranial measurement dataset) on the palaeolatitudinal data. Possible correlation was analysed using ordinary least squares (OLS) and phylogenetic generalised least squares (PGLS) regressions. *Significant at alpha = 0.05.

| **N** | **OLS** | | | | **PGLS** | | | |
| --- | --- | --- | --- | --- | --- | --- | --- | --- |
| 178 | **R^2^** | **Intercept** | **Slope** | **AIC** | **R^2^** | **Intercept** | **Slope** | **AIC** |
|  | 0.022 | 2.595 | -0.004* (0.024) | 150.74 | 0.019 | 2.195 | -0.002* (0.034) | -19.379 |

Table S18. Results of regressions of log-transformed body length proxy (using only marine species in the ODCL cranial measurement dataset) on the palaeolatitudinal data. Possible correlation was analysed using ordinary least squares (OLS) and phylogenetic generalised least squares (PGLS) regressions. *Significant at alpha = 0.05.

| **N** | **OLS** | | | | **PGLS** | | | |
| --- | --- | --- | --- | --- | --- | --- | --- | --- |
| 48 | **R^2^** | **Intercept** | **Slope** | **AIC** | **R^2^** | **Intercept** | **Slope** | **AIC** |
|  | -0.019 | 2.289 | -0.0008 (0.739) | -21.925 | 0.035 | 2.289 | -0.003 (0.105) | -36.771 |

Table S19. Results of regressions of log-transformed body length proxy (using only marine species in the DCL cranial measurement dataset) on the palaeolatitudinal data. Possible correlation was analysed using ordinary least squares (OLS) and phylogenetic generalised least squares (PGLS) regressions. *Significant at alpha = 0.05.

| **N** | **OLS** | | | | **PGLS** | | | |
| --- | --- | --- | --- | --- | --- | --- | --- | --- |
| 43 | **R^2^** | **Intercept** | **Slope** | **AIC** | **R^2^** | **Intercept** | **Slope** | **AIC** |
|  | 0.014 | 2.873 | -0.002 (0.211) | -28.625 | -0.014 | 2.662 | 0.001 (0.53) | -45.625 |

Table S20. Results of regressions of log-transformed body length proxy (using only non-marine species in the ODCL cranial measurement dataset) on the palaeolatitudinal data. Possible correlation was analysed using ordinary least squares (OLS) and phylogenetic generalised least squares (PGLS) regressions. *Significant at alpha = 0.05.

| **N** | **OLS** | | | | **PGLS** | | | |
| --- | --- | --- | --- | --- | --- | --- | --- | --- |
| 147 | **R^2^** | **Intercept** | **Slope** | **AIC** | **R^2^** | **Intercept** | **Slope** | **AIC** |
|  | 0.037 | 2.09 | -0.003* (0.01) | 14.567 | 0.028 | 1.836 | -0.002* (0.023) | -48.394 |

Table S21. Results of regressions of log-transformed body length proxy (using only non-marine species in the DCL cranial measurement dataset) on the palaeolatitudinal data. Possible correlation was analysed using ordinary least squares (OLS) and phylogenetic generalised least squares (PGLS) regressions. *Significant at alpha = 0.05.

| **N** | **OLS** | | | | **PGLS** | | | |
| --- | --- | --- | --- | --- | --- | --- | --- | --- |
| 135 | **R^2^** | **Intercept** | **Slope** | **AIC** | **R^2^** | **Intercept** | **Slope** | **AIC** |
|  | 0.036 | 2.508 | -0.005* (0.014) | 102.424 | 0.06 | 2.259 | -0.004* (0.002) | 13.242 |

Table S22. Results of regressions of log-transformed body length proxy (using only crocodylian species in the ODCL cranial measurement dataset) on the palaeolatitudinal data. Possible correlation was analysed using ordinary least squares (OLS) and phylogenetic generalised least squares (PGLS) regressions. *Significant at alpha = 0.05.

| **N** | **OLS** | | | | **PGLS** | | | |
| --- | --- | --- | --- | --- | --- | --- | --- | --- |
| 70 | **R^2^** | **Intercept** | **Slope** | **AIC** | **R^2^** | **Intercept** | **Slope** | **AIC** |
|  | 0.175 | 2.265 | -0.004* (0.0001) | -49.408 | 0.034 | 2.194 | -0.002 (0.066) | -46.782 |

Table S23. Results of regressions of log-transformed body length proxy (using only crocodylian species in the DCL cranial measurement dataset) on the palaeolatitudinal data. Possible correlation was analysed using ordinary least squares (OLS) and phylogenetic generalised least squares (PGLS) regressions. *Significant at alpha = 0.05.

| **N** | **OLS** | | | | **PGLS** | | | |
| --- | --- | --- | --- | --- | --- | --- | --- | --- |
| 64 | **R^2^** | **Intercept** | **Slope** | **AIC** | **R^2^** | **Intercept** | **Slope** | **AIC** |
|  | 0.178 | 2.81 | -0.007* (0.0003) | 8.976 | 0.045 | 2.744 | -0.004 (0.05) | -6.629 |

Table S4. Results of regressions of log-transformed body length proxy (using only notosuchian species in the ODCL cranial measurement dataset) on the palaeolatitudinal data. Possible correlation was analysed using ordinary least squares (OLS) and phylogenetic generalised least squares (PGLS) regressions. *Significant at alpha = 0.05.

| **N** | **OLS** | | | | **PGLS** | | | |
| --- | --- | --- | --- | --- | --- | --- | --- | --- |
| 34 | **R^2^** | **Intercept** | **Slope** | **AIC** | **R^2^** | **Intercept** | **Slope** | **AIC** |
|  | 0.012 | 1.849 | 0.003 (0.245) | -8.644 | -0.031 | 1.821 | -0.0001 (0.951) | -20.185 |

Table S25. Results of regressions of log-transformed body length proxy (using only notosuchian species in the DCL cranial measurement dataset) on the palaeolatitudinal data. Possible correlation was analysed using ordinary least squares (OLS) and phylogenetic generalised least squares (PGLS) regressions. *Significant at alpha = 0.05.

| **N** | **OLS** | | | | **PGLS** | | | |
| --- | --- | --- | --- | --- | --- | --- | --- | --- |
| 30 | **R^2^** | **Intercept** | **Slope** | **AIC** | **R^2^** | **Intercept** | **Slope** | **AIC** |
|  | -0.035 | 2.26 | 0.0002 (0.945) | 14.931 | 0.035 | 2.274 | -0.005 (0.162) | -4.677 |

Table S26. Results of regressions of log-transformed body length proxy (using only thalattosuchian species in the ODCL cranial measurement dataset) on the palaeolatitudinal data. Possible correlation was analysed using ordinary least squares (OLS) and phylogenetic generalised least squares (PGLS) regressions. *Significant at alpha = 0.05.

| **N** | **OLS** | | | | **PGLS** | | | |
| --- | --- | --- | --- | --- | --- | --- | --- | --- |
| 30 | **R^2^** | **Intercept** | **Slope** | **AIC** | **R^2^** | **Intercept** | **Slope** | **AIC** |
|  | -0.019 | 2.115 | 0.004 (0.509) | -4.607 | -0.035 | 2.156 | -0.0001 (0.977) | -19.849 |

Table S27. Results of regressions of log-transformed body length proxy (using only thalattosuchian species in the DCL cranial measurement dataset) on the palaeolatitudinal data. Possible correlation was analysed using ordinary least squares (OLS) and phylogenetic generalised least squares (PGLS) regressions. *Significant at alpha = 0.05.

| **N** | **OLS** | | | | **PGLS** | | | |
| --- | --- | --- | --- | --- | --- | --- | --- | --- |
| 26 | **R^2^** | **Intercept** | **Slope** | **AIC** | **R^2^** | **Intercept** | **Slope** | **AIC** |
|  | -0.004 | 2.579 | 0.004 (0.357) | -14.111 | 0.01 | 2.601 | 0.003 (0.273) | -28.503 |

Table S28. Results of regressions of log-transformed body length proxy (using only tethysuchian species in the ODCL cranial measurement dataset) on the palaeolatitudinal data. Possible correlation was analysed using ordinary least squares (OLS) and phylogenetic generalised least squares (PGLS) regressions. *Significant at alpha = 0.05.

| **N** | **OLS** | | | | **PGLS** | | | |
| --- | --- | --- | --- | --- | --- | --- | --- | --- |
| 16 | **R^2^** | **Intercept** | **Slope** | **AIC** | **R^2^** | **Intercept** | **Slope** | **AIC** |
|  | 0.251 | 2.468 | -0.009* (0.027) | -5.781 | 0.444 | 2.54 | -0.012* (0.002) | -6.853 |

Table S29. Results of regressions of log-transformed body length proxy (using only tethysuchian species in the DCL cranial measurement dataset) on the palaeolatitudinal data. Possible correlation was analysed using ordinary least squares (OLS) and phylogenetic generalised least squares (PGLS) regressions. *Significant at alpha = 0.05.

| **N** | **OLS** | | | | **PGLS** | | | |
| --- | --- | --- | --- | --- | --- | --- | --- | --- |
| 14 | **R^2^** | **Intercept** | **Slope** | **AIC** | **R^2^** | **Intercept** | **Slope** | **AIC** |
|  | -0.002 | 2.898 | -0.004 (0.345) | -1.387 | -0.084 | 2.904 | 0.001 (0.729) | -2.537 |

# Supplementary references

Andrade MB, Edmonds R, Benton MJ, Schouten R. **2011.** A new Berriasian species of *Goniopholis* (Mesoeucrocodylia, Neosuchia) from England, and a review of the genus. *Zoological Journal of the Linnean Society,* 163S1, S66–S108.

Bapst DW. **2012.** paleotree: an R package for paleontological and phylogenetic analyses of evolution. *Methods in Ecology and Evolution*, 3(5), 803–07.

Bapst DW. **2013.** A stochastic rate-calibrated method for time-scaling phylogenies of fossil taxa. *Methods in Ecology and Evolution*, 4(8), 724–33.

Bapst DW. **2014.** Preparing paleontological datasets for phylogenetic comparative methods. In: Garamszegi LZ (ed.) *Modern phylogenetic comparative methods and their application in evolutionary biology*. Berlin: Springer. p. 515–44.

Bapst DW. **2014.** Assessing the effect of time-scaling methods on phylogeny-based analyses in the fossil record. *Paleobiology*, 40(3), 331–51.

Bates KT, Manning PL, Hodgetts D, Sellers WI. **2009.** Estimating mass properties of dinosaurs using laser imaging and 3D computer modelling. *PLoS One*, 4, e4532.

Benson RBJ, Campione NE, Carrano MT, Mannion PD, Sullivan C, Upchurch P, Evans DC. **2014.** Rates of dinosaur body mass evolution indicate 170 million years of sustained ecological innovation on the avian stem lineage. *PLoS Biology*, 12(5), e1001853.

Benson RBJ, Hunt G, Carrano MT, Campione N. **2018.** Cope's rule and the adaptive landscape of dinosaur body size evolution. *Palaeontology*, 61(1), 13–48.

Brochu CA. **2012.** Phylogenetic relationships of Palaeogene ziphodont eusuchians and the status of *Pristichampsus* Gervais, 1853. *Earth and Environmental Science Transactions of the Royal Society of Edinburgh*, 103(3-4), 521–550.

Brochu CA, Parris DC, Grandstaff BS, Denton Jr RK, Gallagher WB. **2012.** A new species of *Borealosuchus* (Crocodyliformes, Eusuchia) from the Late Cretaceous–early Paleogene of New Jersey. *Journal of Vertebrate Paleontology*, 32(1), 105–116.

Brocklehurst N. **2017.** Rates of morphological evolution in Captorhinidae: an adaptive radiation of Permian herbivores. *PeerJ*, 5, e3200.

Bronzati M, Montefeltro FC, Langer MC. **2015.** Diversification events and the effects of mass extinctions on Crocodyliformes evolutionary history. *Royal Society Open Science,* 2, 140385.

Buscalioni ÁD. **2017.** The Gobiosuchidae in the early evolution of Crocodyliformes. *Journal of Vertebrate Paleontology*, 37(3), e1324459.

Bustard HR, Singh LAK. **1977.** Studies on the Indian Gharial *Gavialis gangeticus* (Gmelin) (Reptilia, Crocodilia) – I: Estimation of body length from scute length. *Indian Forester*, 103(2), 140–149.

Campione NE, Evans DC. **2012.** A universal scaling relationship between body mass and proximal limb bone dimensions in quadrupedal terrestrial tetrapods. *BMC Biology*, 10(1), 60.

Carballido JL, Pol D, Otero A, Cerda IA, Salgado L, Garrido AC, Ramezani J, Cúneo NR, Krause JM. **2017.** A new giant titanosaur sheds light on body mass evolution among sauropod dinosaurs. *Proceedings of the Royal Society B: Biological Sciences*, 284(1860), 20171219.

Clark JM. **1994.** Patterns of evolution in Mesozoic Crocodyliformes. In: Fraser NC, Sues HD (eds.) *In the Shadow of Dinosaurs*. Cambridge: Cambridge University Press. p. 84–97.

Clark JM. **2011.** A new shartegosuchid crocodyliform from the Upper Jurassic Morrison Formation of western Colorado. *Zoological Journal of the Linnean Society,* 163S1, S152–S172.

Colbert EH. **1962.** The weights of dinosaurs. *American Museum Novitates*, (2076), 1–16.

Currie PJ. **1978.** The orthometric linear unit. *Journal of Paleontology*, 52, 964–971.

Farlow JO, Hurlburt GR, Elsey RM, Britton AR, Langston W. **2005.** Femoral dimensions and body size of *Alligator mississippiensis*: estimating the size of extinct mesoeucrocodylians. *Journal of Vertebrate Paleontology*, 25(2), 354–369.

Godoy PL, Bronzati M, Eltink E, Marsola JCA, Cidade GM, Langer MC, Montefeltro FC. **2016.** Postcranial anatomy of *Pissarrachampsa sera* (Crocodyliformes, Baurusuchidae) from the Late Cretaceous of Brazil: insights on lifestyle and phylogenetic significance. *PeerJ*, 4, e2075.

Hastings AK, Bloch JI, Jaramillo CA. **2015.** A new blunt-snouted dyrosaurid, *Anthracosuchus balrogus* gen. et sp. nov. (Crocodylomorpha, Mesoeucrocodylia), from the Palaeocene of Colombia. *Historical Biology*, 27(8), 998–1020.

Hedman MM. **2010.** Constraints on clade ages from fossil outgroups. *Paleobiology*, 36(1), 16–31.

Herrera Y, Gasparini Z, Fernández MS. **2015.** *Purranisaurus potens* Rusconi, an enigmatic metriorhynchid from the Late Jurassic–Early Cretaceous of the Neuquén Basin. *Journal of Vertebrate Paleontology*, 35(2), e904790.

Hall PM, Portier KM. **1994.** Cranial morphometry of New Guinea crocodiles (*Crocodylus novaeguineae*): ontogenetic variation in relative growth of the skull and an assessment of its utility as a predictor of the sex and size of individuals. *Herpetological Monographs*, 203–225.

Hurlburt G. **1999.** Comparison of body mass estimation techniques, using recent reptiles and the pelycosaur *Edaphosaurus boanerges*. *Journal of Vertebrate Paleontology*, 19(2), 338–350.

Hurlburt GR, Heckert AB, Farlow JO. **2003.** Body mass estimates of phytosaurs (Archosauria: Parasuchidae) from the Petrified Forest Formation (Chinle Group: Revueltian) based on skull and limb bone measurements. *New Mexico Museum of Natural History and Science Bulletins*, 24, 105–13.

Laurin M. **2004.** The evolution of body size, Cope's rule and the origin of amniotes. *Systematic Biology*, 53(4), 594–622.

Leardi JM, Pol D, Clark JM. **2017.** Detailed anatomy of the braincase of *Macelognathus vagans* Marsh, 1884 (Archosauria, Crocodylomorpha) using high resolution tomography and new insights on basal crocodylomorph phylogeny. *PeerJ*, 5, e2801.

Lloyd GT, Bapst DW, Friedman M, Davis KE. **2016.** Probabilistic divergence time estimation without branch lengths: dating the origins of dinosaurs, avian flight and crown birds. *Biology letters*, 12(11), 20160609.

Maddison WP, Maddison DR. **2018.** Mesquite: a modular system for evolutionary analysis. Version 3.40. http://mesquiteproject.org

Martin JE, De’lfino M, Smith T. **2016.** Osteology and affinities of Dollo's goniopholidid (Mesoeucrocodylia) from the Early Cretaceous of Bernissart, Belgium. *Journal of Vertebrate Paleontology*, 36(6), e1222534.

Meunier LMV, Larsson HCE. **2017.** Revision and phylogenetic affinities of *Elosuchus* (Crocodyliformes). *Zoological Journal of the Linnean Society,* 179, 169–200.

Montefeltro FC, Larsson HCE, França, MAG, Langer MC. **2013.** A new neosuchian with Asian affinities from the Jurassic of northeastern Brazil. *Naturwissenschaften*, 100(9), 835–841.

Motani R. **2001.** Estimating body mass from silhouettes: testing the assumption of elliptical body cross-sections. *Paleobiology*, 27, 735–50.

Narváez I, Brochu CA, Escaso F, Pérez-García A, Ortega F. **2015.** New Crocodyliforms from Southwestern Europe and Definition of a Diverse Clade of European Late Cretaceous Basal Eusuchians. *PLoS ONE,* 10(11), e0140679.

Platt SG, Rainwater TR, Thorbjarnarson JB, Finger AG, Anderson TA, McMurry ST. **2009.** Size estimation, morphometrics, sex ratio, sexual size dimorphism, and biomass of Morelet's crocodile in northern Belize. *Caribbean Journal of Science*, 45(1), 80–94.

Platt SG, Rainwater TR, Thorbjarnarson JB, Martin D. **2011.** Size estimation, morphometrics, sex ratio, sexual size dimorphism, and biomass of *Crocodylus acutus* in the coastal zone of Belize. *Salamandra*; 47, 179–92.

Pol D, Gasparini Z. **2009.** Skull anatomy of *Dakosaurus* *andiniensis* (Thalattosuchia: Crocodylomorpha) and the phylogenetic position of Thalattosuchia. *Journal of Systematic Palaeontology,* 7(2), 163–197.

Pol D, Leardi JM, Lecuona A, Krause M. **2012.** Postcranial anatomy of *Sebecus icaeorhinus* (Crocodyliformes, Sebecidae) from the Eocene of Patagonia. *Journal of Vertebrate Paleontology*, 32(2), 328–354.

Pol D, Rauhut OWM, Lecuona A, Leardi JM, Xu X, Clark JM. **2013.** A new fossil from the Jurassic of Patagonia reveals the early basicranial evolution and the origins of Crocodyliformes. *Biological Reviews*. 88, 862–872.

Pol D, Nascimento PM, Carvalho AB, Riccomini C, Pires-Domingues RA, Zaher H. **2014.** A New Notosuchian from the Late Cretaceous of Brazil and the Phylogeny of Advanced Notosuchians. *PLoS ONE,* 9(4), e93105.

R Core Team. **2018.** R: a language and environment for statistical computing. Vienna: R Foundation for Statistical Computing. https://www.R-project.org/.

Ristevski J, Young MT, Andrade MB, Hastings AK. **2018.** A new species of *Anteophthalmosuchus* (Crocodylomorpha, Goniopholididae) from the Lower Cretaceous of the Isle of Wight, United Kingdom, and a review of the genus. *Cretaceous Research*, 84, 340–383.

Romer AS, Price LW. **1940.** Review of the Pelycosauria. *Geological Society of America Special Papers*, 28, 1–534.

Scheyer TM, Aguilera OA, Delfino M, Fortier DC, Carlini AA, Sánchez R, Carrillo-Briceño JD, Quiroz L, Sánchez-Villagra MR. **2013.** Crocodylian diversity peak and extinction in the late Cenozoic of the northern Neotropics. *Nature communications*, 4, 1907.

Schwarz D, Raddatz M, Wings O. **2017.** *Knoetschkesuchus langenbergensis* gen. nov. sp. nov., a new atoposaurid crocodyliform from the Upper Jurassic Langenberg Quarry (Lower Saxony, northwestern Germany), and its relationships to Theriosuchus. *PLoS ONE*, 12(2), e0160617.

Sellers WI, Hepworth-Bell J, Falkingham PL, Bates KT, Brassey CA, Egerton VM, Manning PL. **2012.** Minimum convex hull mass estimations of complete mounted skeletons. *Biology Letters*, 8(5), 842–845.

Sereno PC, Larsson HC, Sidor CA, Gado B. **2001.** The giant crocodyliform *Sarcosuchus* from the Cretaceous of Africa. *Science*, 294(5546), 1516–1519.

Tennant JP, Mannion PD, Upchurch P. **2016.** Evolutionary relationships and systematics of Atoposauridae (Crocodylomorpha: Neosuchia): implications for the rise of Eusuchia. *Zoological Journal of the Linnean Society,* 177(4), 854–936.

Turner AH. **2015.** A review of *Shamosuchus* and *Paralligator* (Crocodyliformes, Neosuchia) from the Cretaceous of Asia. *PLoS ONE*, 10(2), e0118116.

Turner AH, Pritchard AC. **2015.** The monophyly of Susisuchidae (Crocodyliformes) and its phylogenetic placement in Neosuchia. *PeerJ,* 3, e759.

Webb GJW, Messel H. **1978.** Morphometric analysis of *Crocodylus porosus* from the north coast of Arnhem Land, northern Australia. *Australian Journal of Zoology*, 26(1), 1–27.

Wilberg E. **2015.** What's in an Outgroup? The Impact of Outgroup Choice on the Phylogenetic Position of Thalattosuchia (Crocodylomorpha) and the Origin of Crocodyliformes. *Systematic Biology,* 64(4), 621–37.

Young MT. **2014.** Filling the “Corallian Gap”: re-description of a metriorhynchid crocodylomorph from the Oxfordian (Late Jurassic) of Headington, England. *Historical Biology,* 26, 80–90.

Young MT, Bell MA, Andrade MB, Brusatte SL. **2011.** Body size estimation and evolution in metriorhynchid crocodylomorphs: implications for species diversification and niche partitioning. *Zoological Journal of the Linnean Society*, 163(4), 1199–1216.

Young MT, Rabi M, Bell MA, Foffa, D, Steel L, Sachs S, Peyer K. **2016.** Big-headed marine crocodyliforms and why we must be cautious when using extant species as body length proxies for long-extinct relatives. *Palaeontologia Electronica*, 19(3), 1–14.

Young MT, Hastings AK, Allain R, Smith TJ. **2017.** Revision of the enigmatic crocodyliform *Elosuchus felixi* de Lapparent de Broin, 2002 from the Lower-Upper Cretaceous boundary of Niger: potential evidence for an early origin of the clade Dyrosauridae. *Zoological Journal of the Linnean Society,* 179, 377–403.
