# Supplementary figures and images for: The multi-peak adaptive landscape of crocodylomorph body size evolution
Source: BMC Evol Biol. 2019 Aug 7;19:167. doi: 10.1186/s12862-019-1466-4 (PMC6686447; doi:10.1186/s12862-019-1466-4)

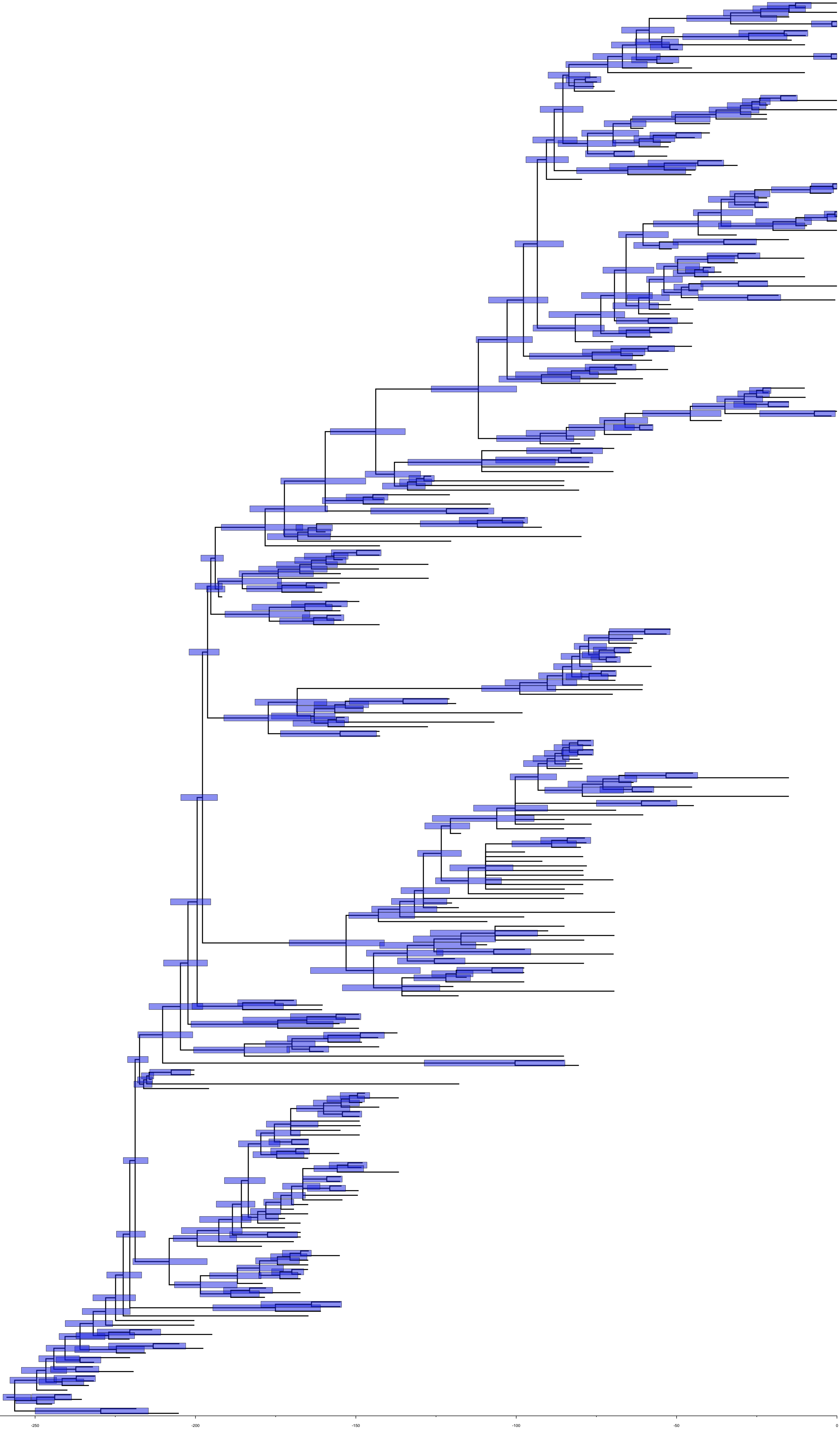

Supplement: Supplementary file 4 — ZIP-archive containing plots of all SURFACE model fits. (ZIP 1189 kb) [file 12862_2019_1466_MOESM4_ESM.zip › Supp Figures/alternative_tree.pdf]

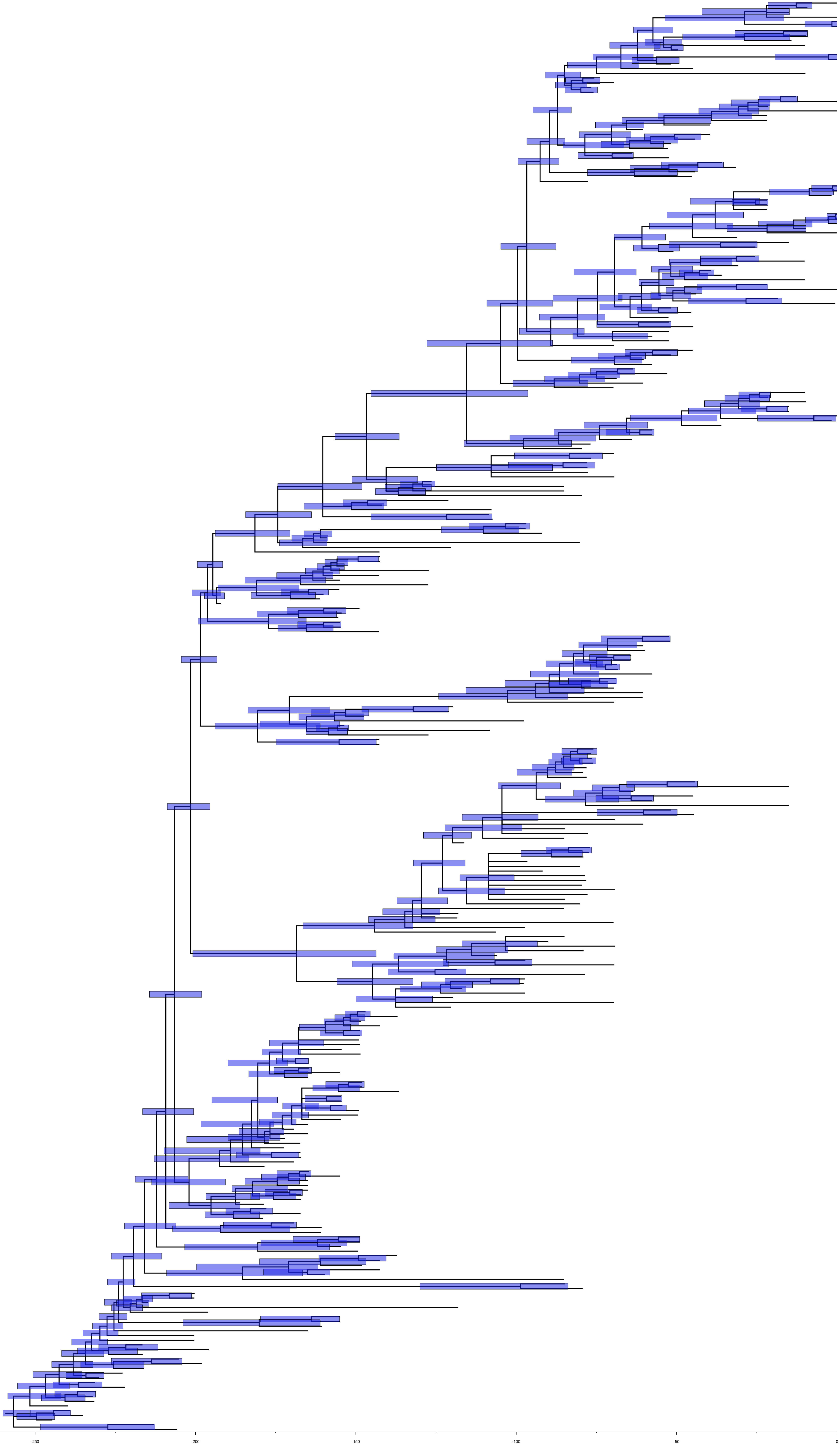

Supplement: Supplementary file 4 — ZIP-archive containing plots of all SURFACE model fits. (ZIP 1189 kb) [file 12862_2019_1466_MOESM4_ESM.zip › Supp Figures/alternative_tree_2.pdf]

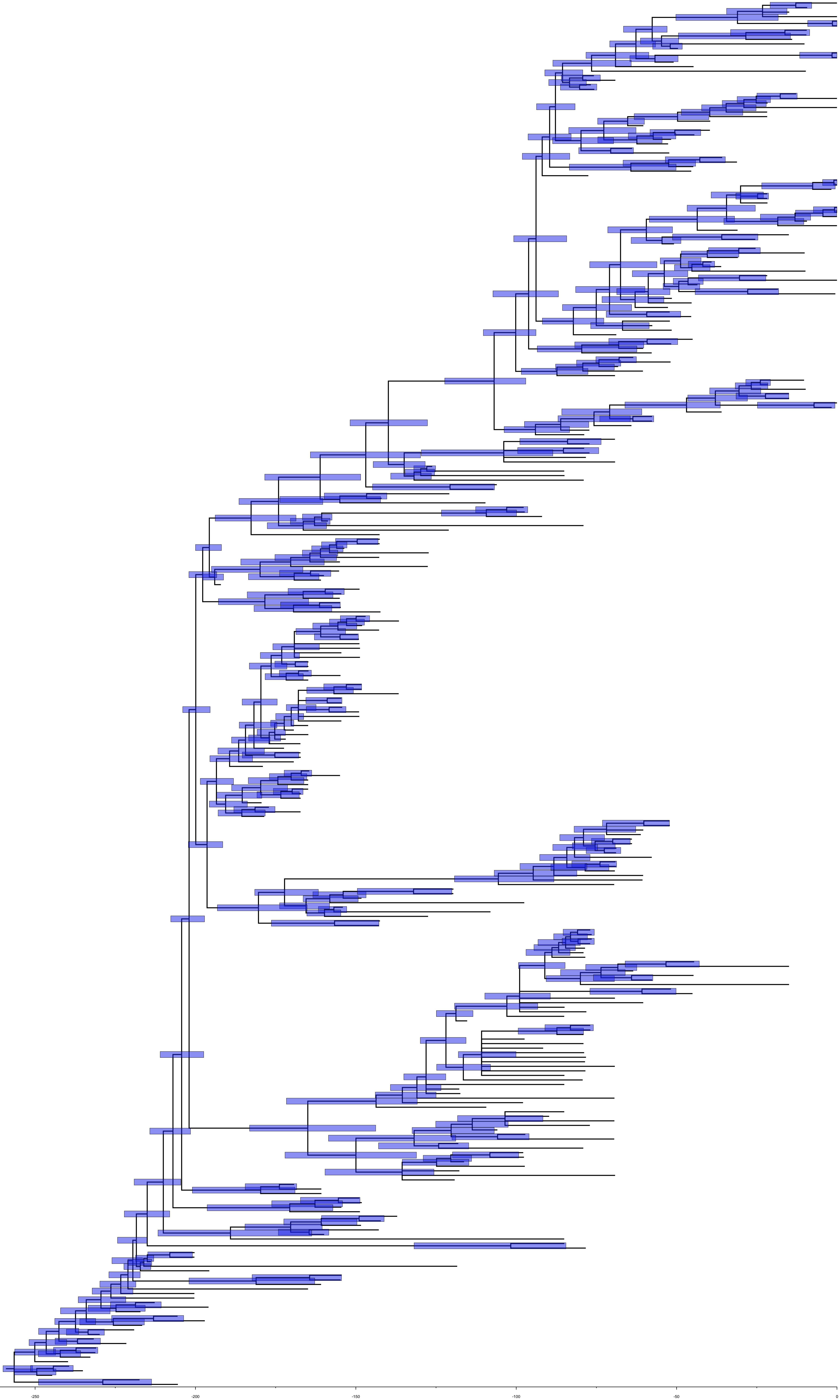

Supplement: Supplementary file 4 — ZIP-archive containing plots of all SURFACE model fits. (ZIP 1189 kb) [file 12862_2019_1466_MOESM4_ESM.zip › Supp Figures/croc_tree.pdf]

**a**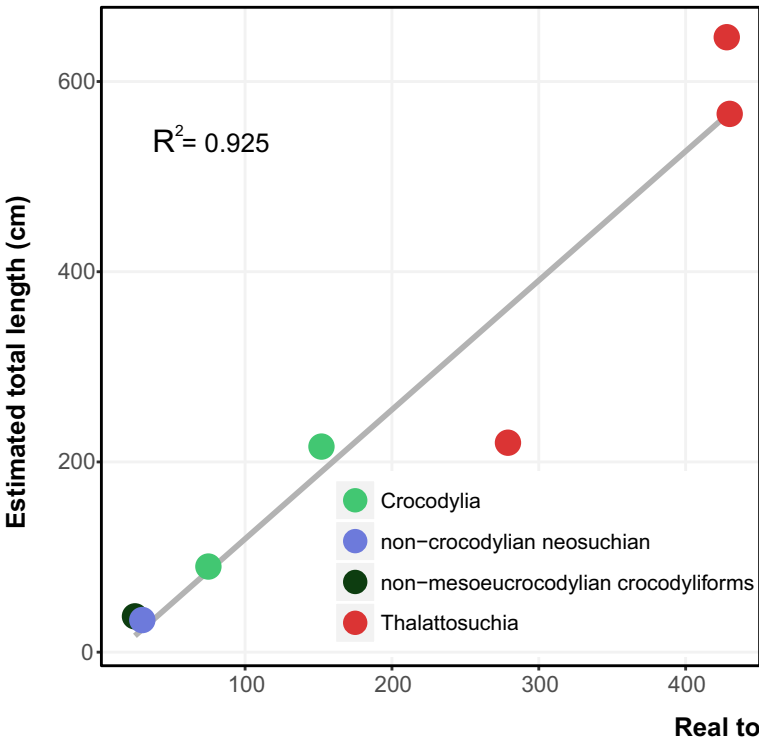**b**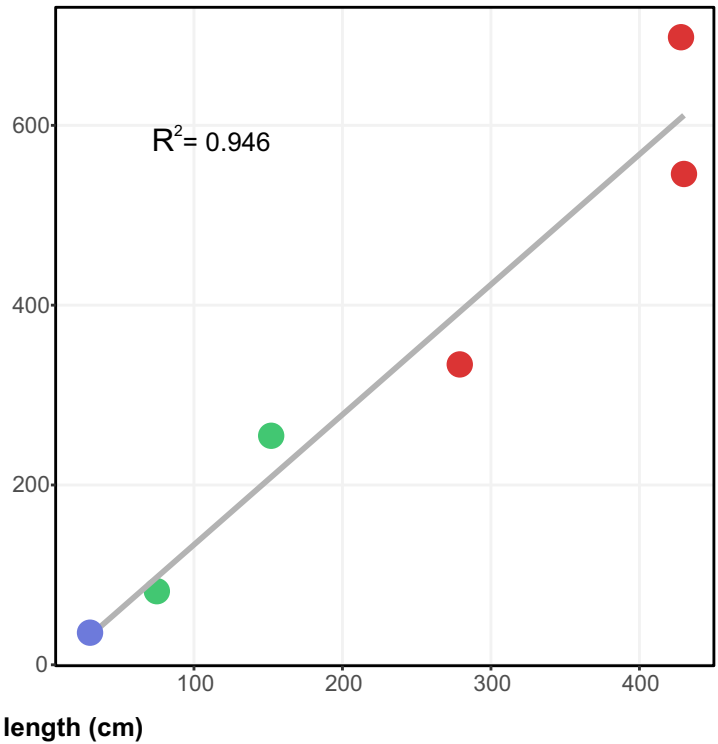

Supplement: Supplementary file 4 — ZIP-archive containing plots of all SURFACE model fits. (ZIP 1189 kb) [file 12862_2019_1466_MOESM4_ESM.zip › Supp Figures/Fig S1.pdf]

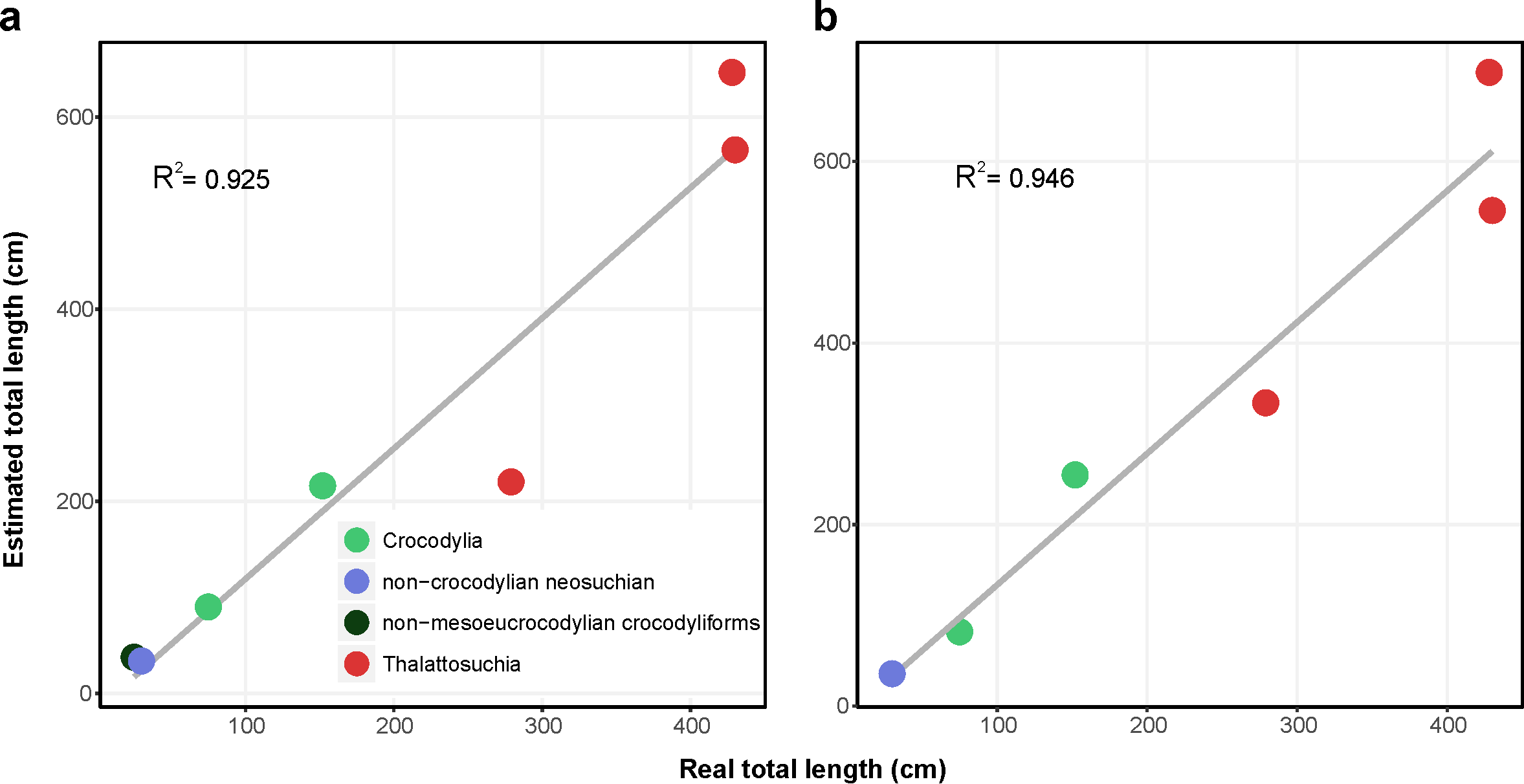

Supplement: Supplementary file 4 — ZIP-archive containing plots of all SURFACE model fits. (ZIP 1189 kb) [file 12862_2019_1466_MOESM4_ESM.zip › Supp Figures/Fig S1.tif]

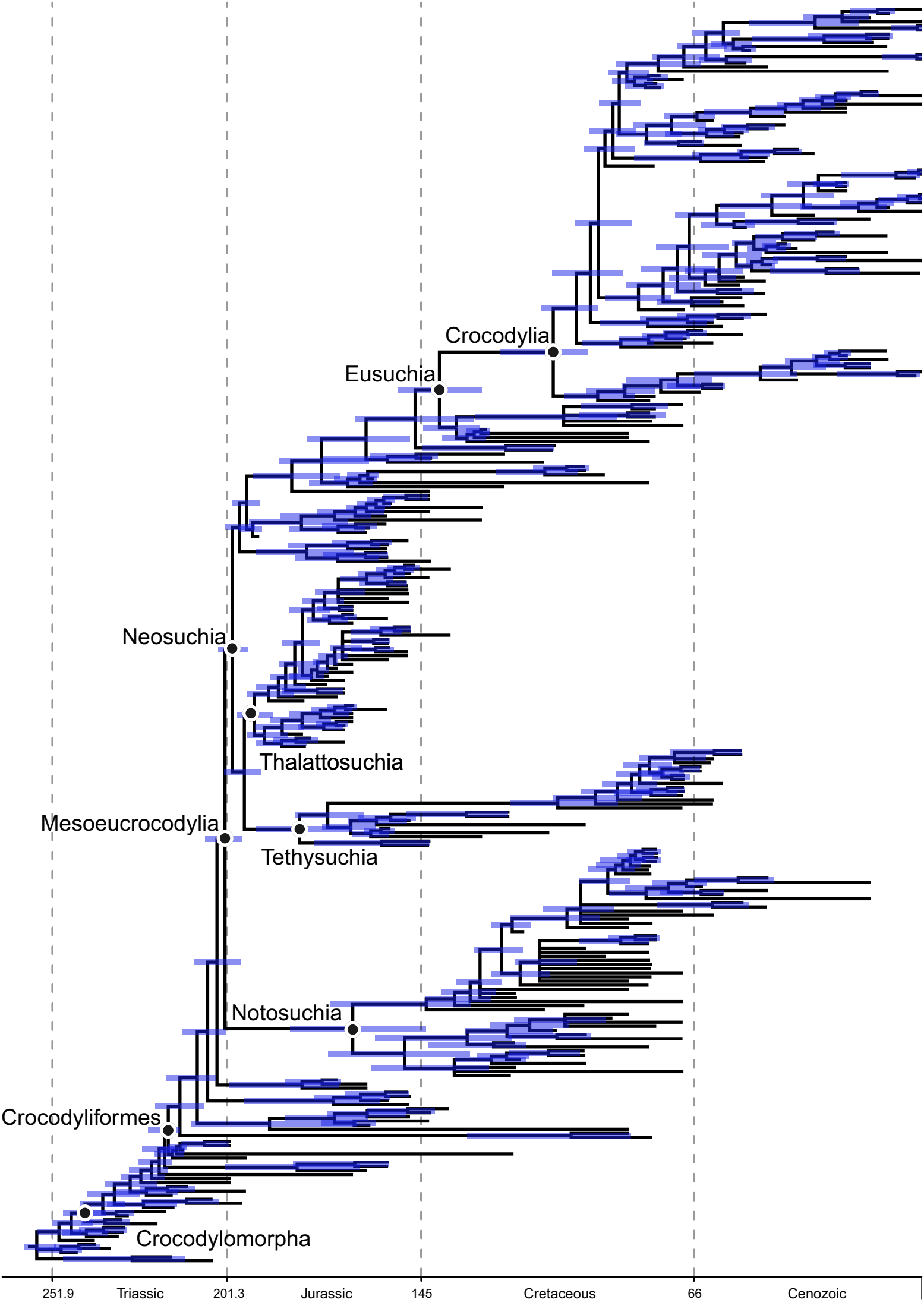

Supplement: Supplementary file 4 — ZIP-archive containing plots of all SURFACE model fits. (ZIP 1189 kb) [file 12862_2019_1466_MOESM4_ESM.zip › Supp Figures/Fig S2.pdf]

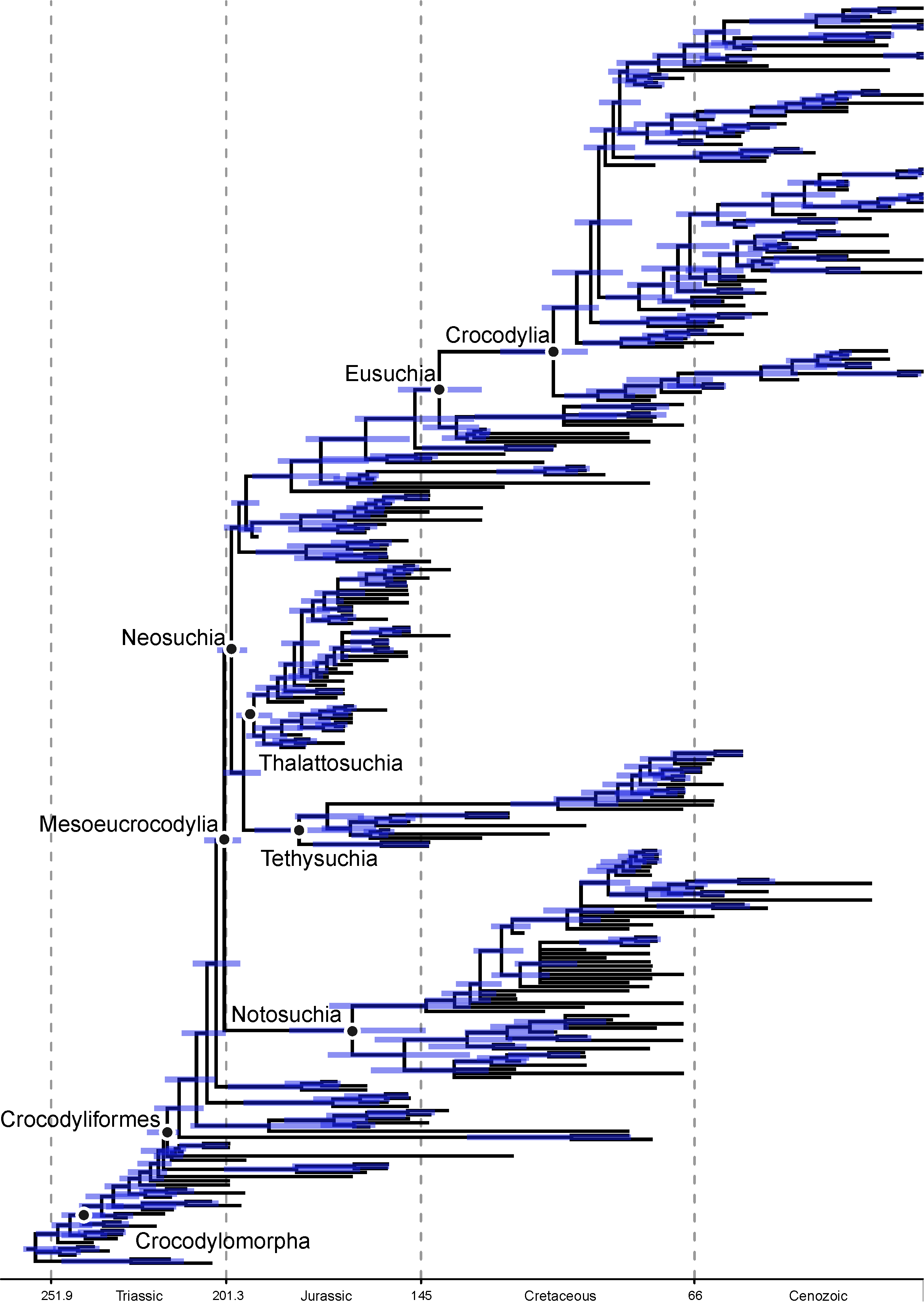

Supplement: Supplementary file 4 — ZIP-archive containing plots of all SURFACE model fits. (ZIP 1189 kb) [file 12862_2019_1466_MOESM4_ESM.zip › Supp Figures/Fig S2.tif]

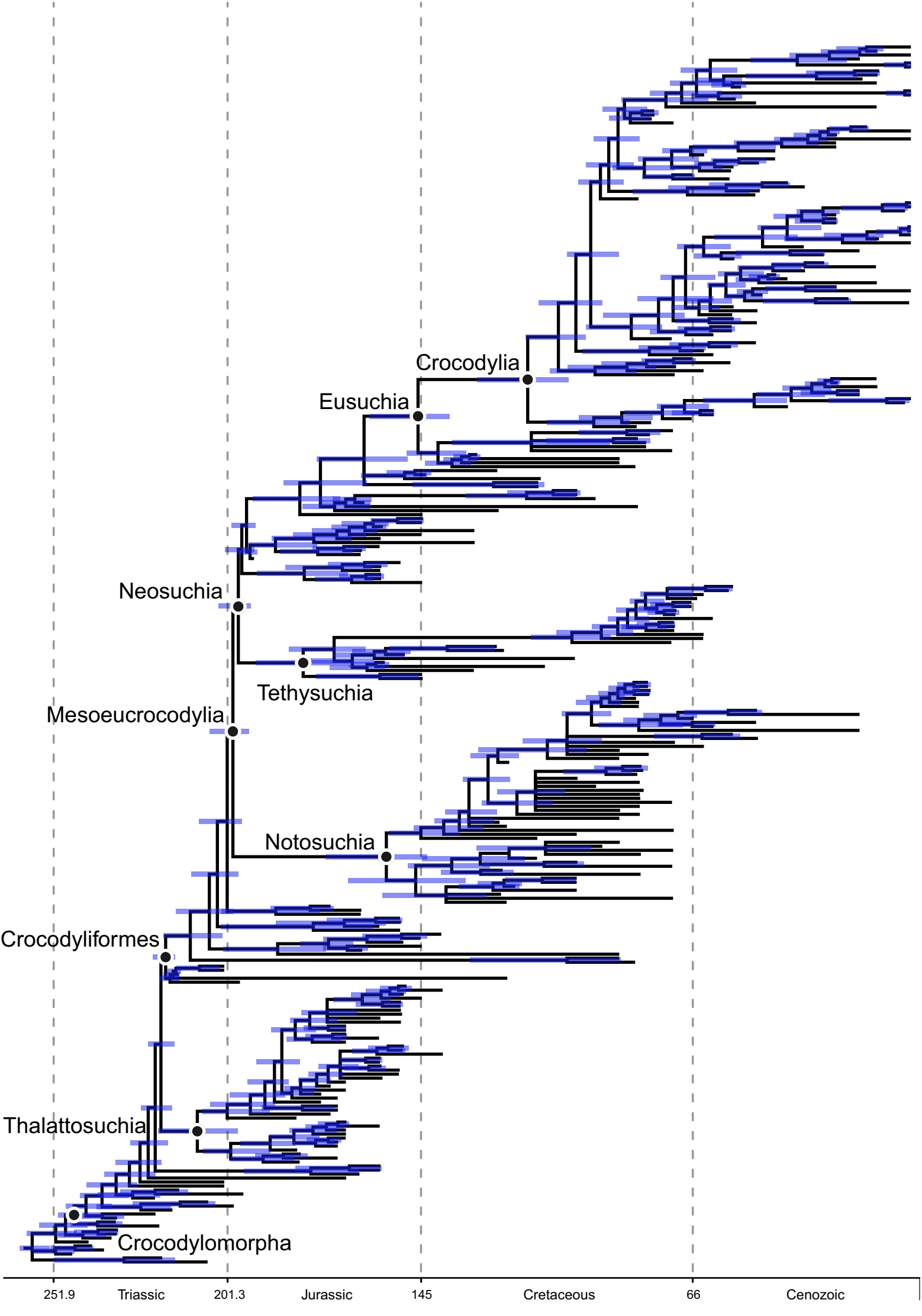

Supplement: Supplementary file 4 — ZIP-archive containing plots of all SURFACE model fits. (ZIP 1189 kb) [file 12862_2019_1466_MOESM4_ESM.zip › Supp Figures/Fig S3.pdf]

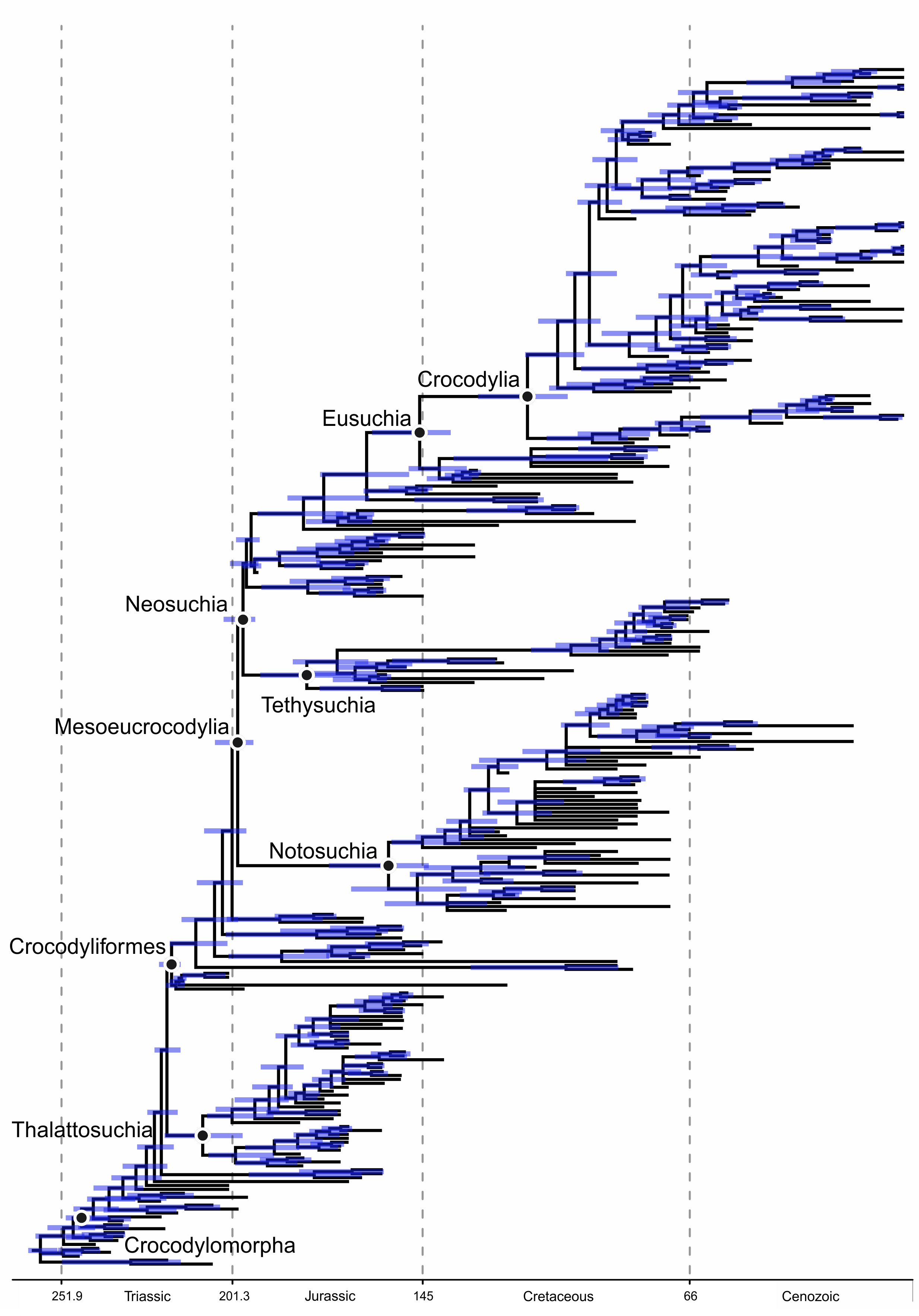

Supplement: Supplementary file 4 — ZIP-archive containing plots of all SURFACE model fits. (ZIP 1189 kb) [file 12862_2019_1466_MOESM4_ESM.zip › Supp Figures/Fig S3.tif]

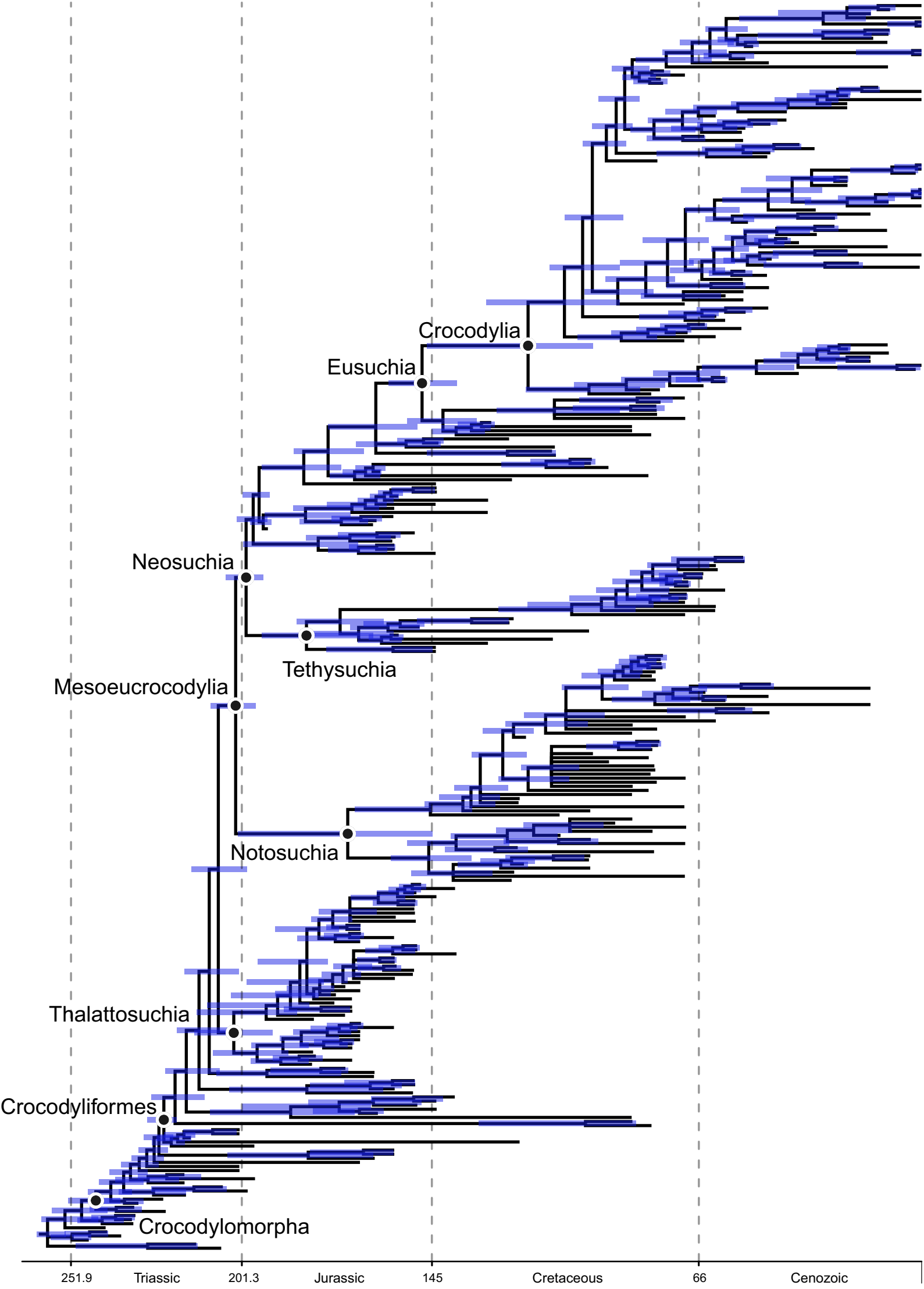

Supplement: Supplementary file 4 — ZIP-archive containing plots of all SURFACE model fits. (ZIP 1189 kb) [file 12862_2019_1466_MOESM4_ESM.zip › Supp Figures/Fig S4.pdf]

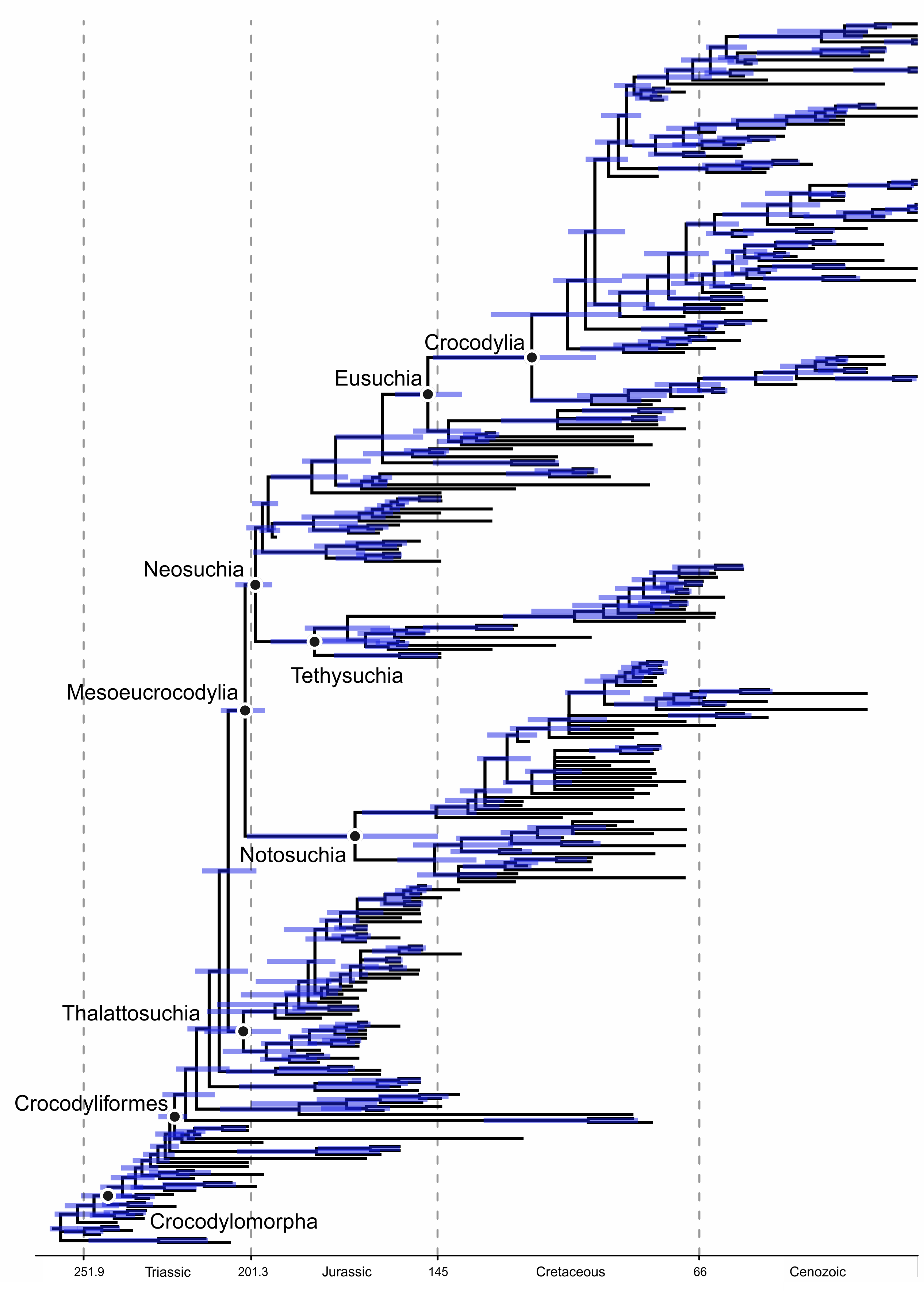

Supplement: Supplementary file 4 — ZIP-archive containing plots of all SURFACE model fits. (ZIP 1189 kb) [file 12862_2019_1466_MOESM4_ESM.zip › Supp Figures/Fig S4.tif]

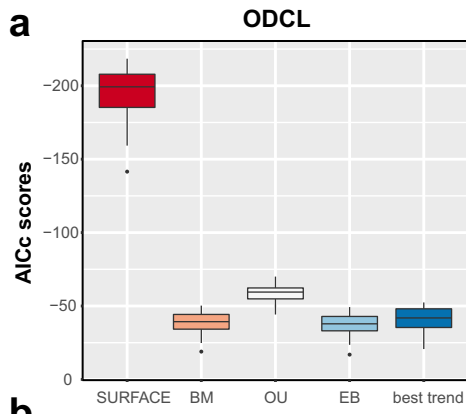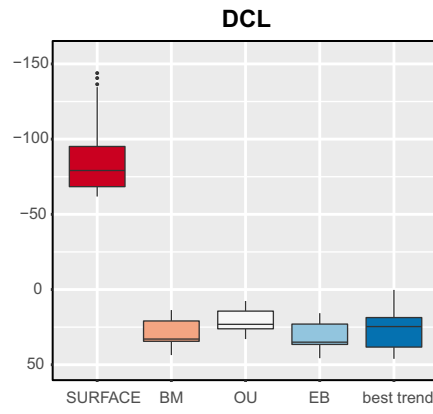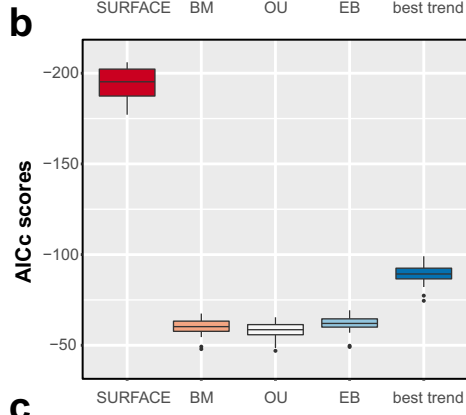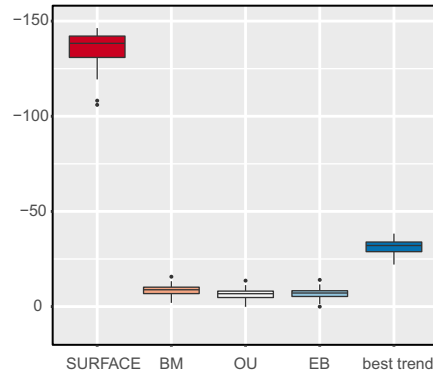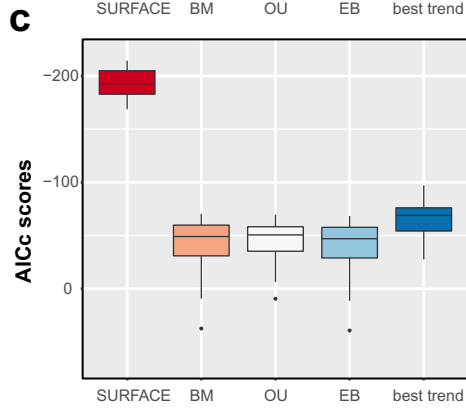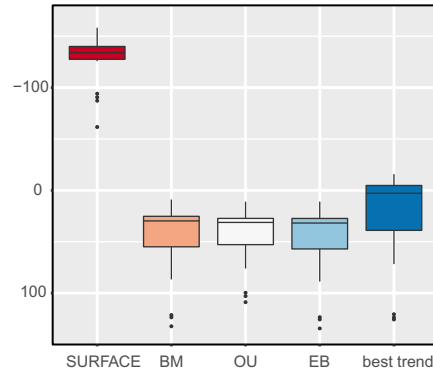

Supplement: Supplementary file 4 — ZIP-archive containing plots of all SURFACE model fits. (ZIP 1189 kb) [file 12862_2019_1466_MOESM4_ESM.zip › Supp Figures/Fig S5.pdf]

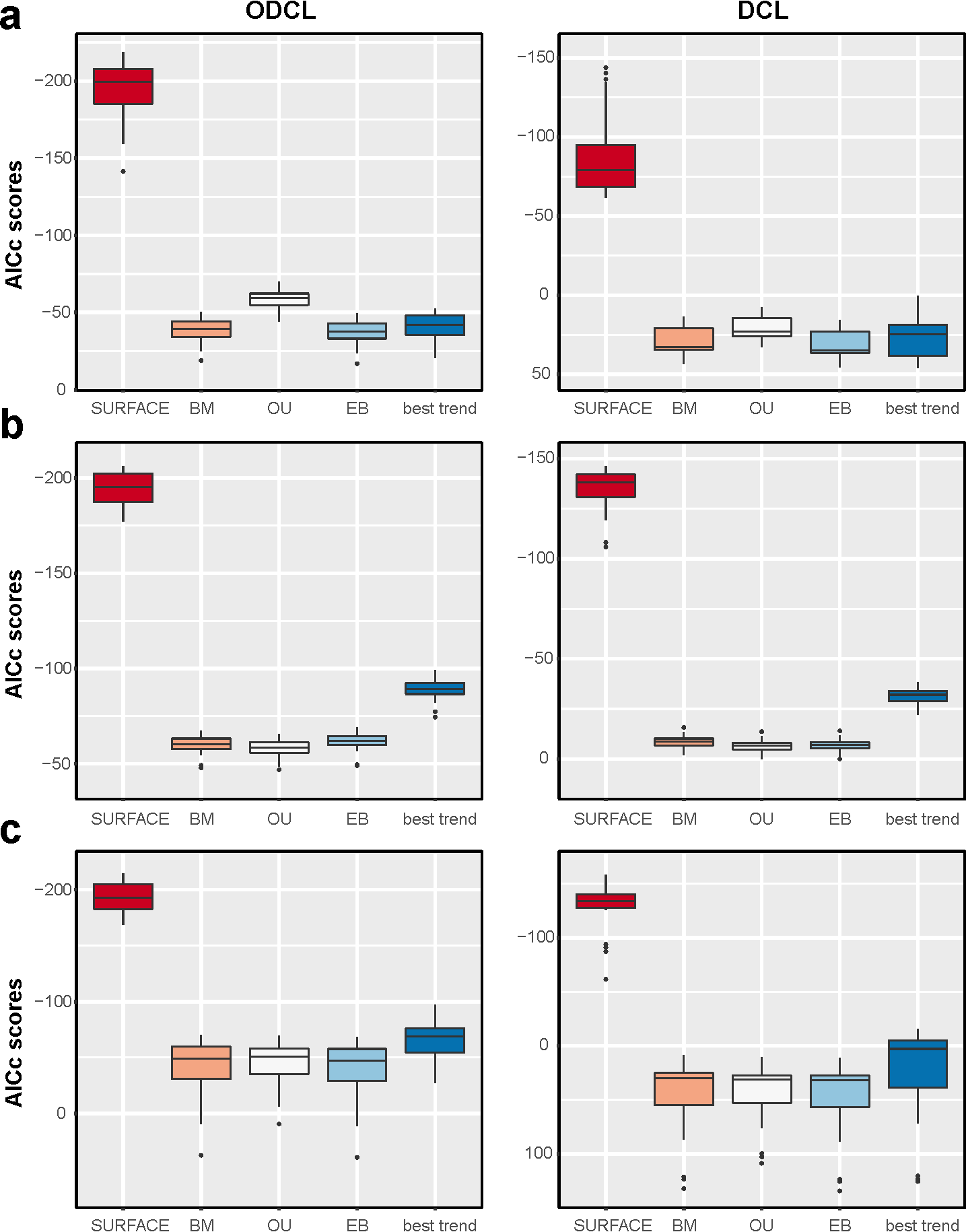

Supplement: Supplementary file 4 — ZIP-archive containing plots of all SURFACE model fits. (ZIP 1189 kb) [file 12862_2019_1466_MOESM4_ESM.zip › Supp Figures/Fig S5.tif]
